# Sodium tungstate promotes vascularization to support beta cell replacement in diabetes

**DOI:** 10.1038/s44321-026-00523-4

**Published:** 2026-09-23

**Authors:** Ainhoa García-Alamán, Marta Fontcuberta-PiSunyer, Jonna M Saarimäki-Vire, Nea Asumaa, Marta Perea-Atienzar, Rebeca Fernandez-Ruiz, Hugo Alves-Figueiredo, Christophe Broca, Joan-Marc Servitja, Ramon Gomis, Diego Balboa, Josep Vidal, Rosa Gasa

**Affiliations:** 1 Institut d’Investigacions Biomèdiques August Pi i Sunyer (IDIBAPS), Barcelona, Spain; 2 CIBER de Diabetes y Enfermedades Metabólicas Asociadas, Instituto de Salud Carlos III, Madrid, Spain; 3 Faculty of Medicine and Health Sciences, Universitat de Barcelona, Barcelona, Spain; 4 Stem Cells and Metabolism Research Program, Faculty of Medicine, University of Helsinki, Helsinki, Finland; 5 Tecnológico de Monterrey, Escuela de Medicina y Ciencias de la Salud, Monterrey, N.L. México; 6 CHU Montpellier, Laboratory of Cell Therapy for Diabetes (LTCD), Hospital St-Eloi, Montpellier, France; 7 Endocrinology and Nutrition Department, Hospital Clinic of Barcelona, Barcelona, Spain

**Keywords:** Metabolism, Stem Cells & Regenerative Medicine, Vascular Biology & Angiogenesis

## Abstract

Insufficient vascularization remains a major obstacle to the success of cell-based therapies for diabetes. Building on prior findings that loss of the phosphatase PTP1B enhances VEGFA production and improves graft vascularization, we investigated sodium tungstate (NaW), a pharmacological phosphatase inhibitor, as a strategy to improve transplantation outcomes. Using human fibroblast-derived insulin-producing cells and human stem cell-derived islets transplanted into the anterior chamber of the eye in immunodeficient mice, we show that NaW treatment significantly increases both vascularized area and insulin-positive tissue area, while reducing apoptosis within transplanted cells. Mechanistically, NaW upregulates VEGFA expression in transplanted cells and amplifies VEGFA-induced endothelial cell proliferation, migration, and tubulogenesis via MAPK/ERK signalling. These dual effects, which encompass stimulating both endocrine and endothelial compartments, lead to enhanced integration and function of transplanted cells. Importantly, the pro-angiogenic effects of NaW occur independently of exogenous endothelial cell supplementation, relying solely on the host’s endogenous endothelial cells. These findings position NaW, and potentially other phosphatase inhibitors, as promising adjuncts to improve vascularization, survival, and therapeutic efficacy in clinical cell-based transplantation protocols for diabetes.

## Synopsis

Building on prior evidence that Protein Tyrosine Phosphatase 1B (PTP1B) deficiency improves mouse islet graft vascularization, sodium tungstate (NaW), a pharmacological protein phosphatase inhibitor, is shown to enhance vascularization and survival of human beta cell grafts.

- NaW increased vascularization in both fibroblast-derived insulin-producing spheroids and stem cell-derived islets transplanted into the anterior chamber of the eye.
- Cell death was decreased and insulin-positive graft area was expanded in NaW-treated animals.
- VEGFA expression was upregulated in transplanted cells from NaW-treated animals.
- NaW potentiated VEGFA-induced endothelial cell proliferation, migration, and tubulogenesis in vitro, in association with activation of MAPK/ERK signalling.
- NaW promoted graft vascularization even in the absence of co-transplanted endothelial cells, indicating that new vessels within the graft were populated by host-derived endothelial cells.

## Abstract (web summary)

Building on prior evidence that Protein Tyrosine Phosphatase 1B (PTP1B) deficiency improves mouse islet graft vascularization, sodium tungstate (NaW), a pharmacological protein phosphatase inhibitor, is shown to enhance vascularization and survival of human beta cell grafts.

## Graphical abstract

**Figure:**
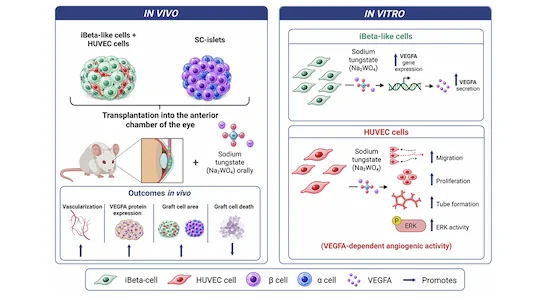

The paper explained **Problem**

Beta cell replacement is a promising cure for type 1 diabetes, but long-term graft survival remains a critical barrier. Delayed vascularization after transplantation creates a hypoxic, nutrient-deprived microenvironment that triggers early beta cell death, resulting in poor graft survival and function. As a consequence, large numbers of cells are required to achieve a therapeutic effect, and repeated transplant procedures may become necessary, a major drawback that limits the clinical viability of this approach.

**Results**

Sodium tungstate, a phosphatase inhibitor with a well-established safety profile in clinical trials, promotes early vascularization of both fibroblast-derived beta-like cells and stem cell-derived islets after transplantation, leading to enhanced graft survival. Mechanistically, sodium tungstate exerts a dual effect: it upregulates the production of the pro-angiogenic factor VEGFA in transplanted cells, while simultaneously potentiating VEGFA-driven prongiogenic activity on endothelial cells.

**Clinical impact**

This study demonstrates that small molecules such as sodium tungstate can be leveraged to enhance post-transplantation vascularization, addressing a key bottleneck in graft survival and opening a new avenue toward more durable beta cell replacement therapies.

## Introduction

The replacement of islet beta cells, whether sourced from donors or generated in vitro as islet/beta-like cells, presents promising prospects for curing type 1 diabetes (T1D) and other forms of severe insulin-dependent diabetes. Nonetheless, achieving the full therapeutic potential of these approaches requires improvements in transplantation methodologies. One major challenge is delayed and insufficient graft vascularization, which results in poor cell survival and function post-transplantation. The success of engraftment heavily depends on overcoming this challenge.

Vascularization is essential for islet survival and function as it enables rapid sensing of blood glucose and coordinated release of insulin and glucagon into the circulation (Peiris et al, 2014). For donor islets, the disruption of vascular connections during isolation, combined with the gradual depletion of intra-islet endothelial cells (iECs) during pre-transplantation islet culture (Xiong et al, 2020; Figueiredo et al, 2019), is a major challenge. Following transplantation, islets must rebuild a functional vascular connection with the host, a process that typically takes two to four weeks (Emamaullee and Shapiro, 2007). This delay in vascularization leads to a period of oxygen and nutrient deprivation for the newly transplanted islets, both of which can affect cell function and viability and lead to cell damage and/or death. Islets are especially sensitive to hypoxic conditions, with ~50–70% of transplanted cells being lost during the isolation, culture, and peri-transplantation stages of treatment (Plesner and Verchere, 2011). This early loss of islet mass ultimately jeopardizes the long-term success of the graft.

On the other hand, the shortage of donor islets for transplantation has prompted the development of in vitro strategies to generate insulin-producing cells, including stem cell-derived islets (SC-islets) from embryonic or induced pluripotent stem cells, as well as through direct reprogramming of somatic cells. These laboratory-generated islet/beta-like cells have no vasculature, which poses an even greater challenge for achieving effective graft vascularization in such therapies. Encapsulation devices, designed to shield transplanted cells from immune rejection and eliminate the need for immunosuppressive therapy, often block vascular integration, exacerbating ischemia and contributing to poor graft outcomes, factors that have caused some clinical trials to be discontinued (Hering et al, 2025). Additionally, trials using semipermeable devices have reported inadequate and uneven engraftment of transplanted cells within the subcutaneous space, an environment characterized by low vascular density (Shapiro et al, 2021; Keymeulen et al, 2024; de Klerk and Hebrok, 2021). To overcome these limitations, strategies such as co-transplantation with endothelial cells (ECs) and other supportive cell types (Takahashi et al, 2018; Song et al, 2019), or the use of microvascular fragments derived from adipose tissue (Nalbach et al, 2021; Aghazadeh et al, 2021), have been investigated with encouraging results. Importantly, enhanced vascularization not only improves graft survival but also aids in the in vivo maturation of lab-generated islets (Jun et al, 2025). Nonetheless, the development of stable, safe, and functional autologous vascular tissue remains a critical hurdle that must be overcome to enable successful clinical translation.

The establishment of new, functional vasculature within the graft requires the activation and recruitment of ECs from both the transplant recipient and iECs within the transplanted islets (Nyqvist et al, 2005; Brissova et al, 2004). The primary pro-angiogenic signal secreted by islet cells, including beta and alpha cells, is vascular endothelial growth factor A (VEGFA)(Peiris et al, 2014). As a result, VEGFA emerged as a key candidate factor for enhancing vascularization and improving islet transplantation outcomes. Nonetheless, optimizing vascularization through external VEGFA delivery remains challenging, as precise dosing is critical: excessive angiogenesis can impair islet architecture and contribute to beta cell loss (Brissova et al, 2014; Agudo et al, 2012; Cai et al, 2012). In a previous study, we demonstrated that islets from mice lacking protein tyrosine phosphatase 1B (PTP1B) showed improved graft revascularization, better survival, and enhanced function, leading to improved transplant outcomes (Figueiredo et al, 2019). We attributed this effect to the increased production and secretion of VEGFA by the transplanted PTP1B-deficient beta cells, highlighting the possibility of boosting endogenous pro-angiogenic pathways in cell implants as a means to accelerate their engraftment.

Building on this evidence, here we sought to determine whether a pharmacological approach could replicate these effects, providing a non-genetic more clinically translatable alternative. For this purpose, we selected tungstate, an oxyanion of tungsten known to function as a broad phosphatase inhibitor. Notably, tungstate effectively inhibits PTP1B with an IC₅₀ of 210 µM (Bellomo et al, 2016; Singh and Maret, 2017). Among its various formulations, sodium tungstate (Na_2_WO_4_, termed NaW henceforth) stands out due to its oral bioavailability, favorable pharmacokinetic properties, and a well-established safety profile (Frawley et al, 2016; National Toxicology Program TR-599, 2021). NaW has been extensively investigated for its anti-diabetic and anti-obesity properties in preclinical models, where it has consistently demonstrated efficacy without evidence of toxicity or adverse effects (Fernández-Alvarez et al, 2004; Claret et al, 2005; Barberà et al, 1994, 2001, 1997; Bertinat et al, 2015). Importantly, its favorable safety profile has also been confirmed following administration in human subjects (Hanzu et al, 2010; Arbat et al, 2025).

In this study, we evaluated the potential of NaW to improve graft vascularization in the context of in vitro-generated cell-based therapies for diabetes. For these experiments, we utilized induced (i) Beta-like cell spheroids generated via direct transcription factor-mediated reprogramming of human fibroblasts (Fontcuberta-PiSunyer et al, 2023), as well as a clinically advanced beta cell replacement platform employing a well-established model of SC-islets, produced through stepwise differentiation of human pluripotent stem cells (Balboa et al, 2022). Our results show that NaW significantly enhances early functional vascularization in both models, resulting in improved graft integration and overall transplantation outcomes. Importantly, this pro-vascular effect appears to be mediated through dual actions, targeting both the transplanted endocrine cells and the host endothelial cells, thereby supporting vascular remodeling and successful engraftment.

## Results

### Inclusion of HUVEC cells improves vascularization of human iBeta-like cell transplants

**Figure 1:**
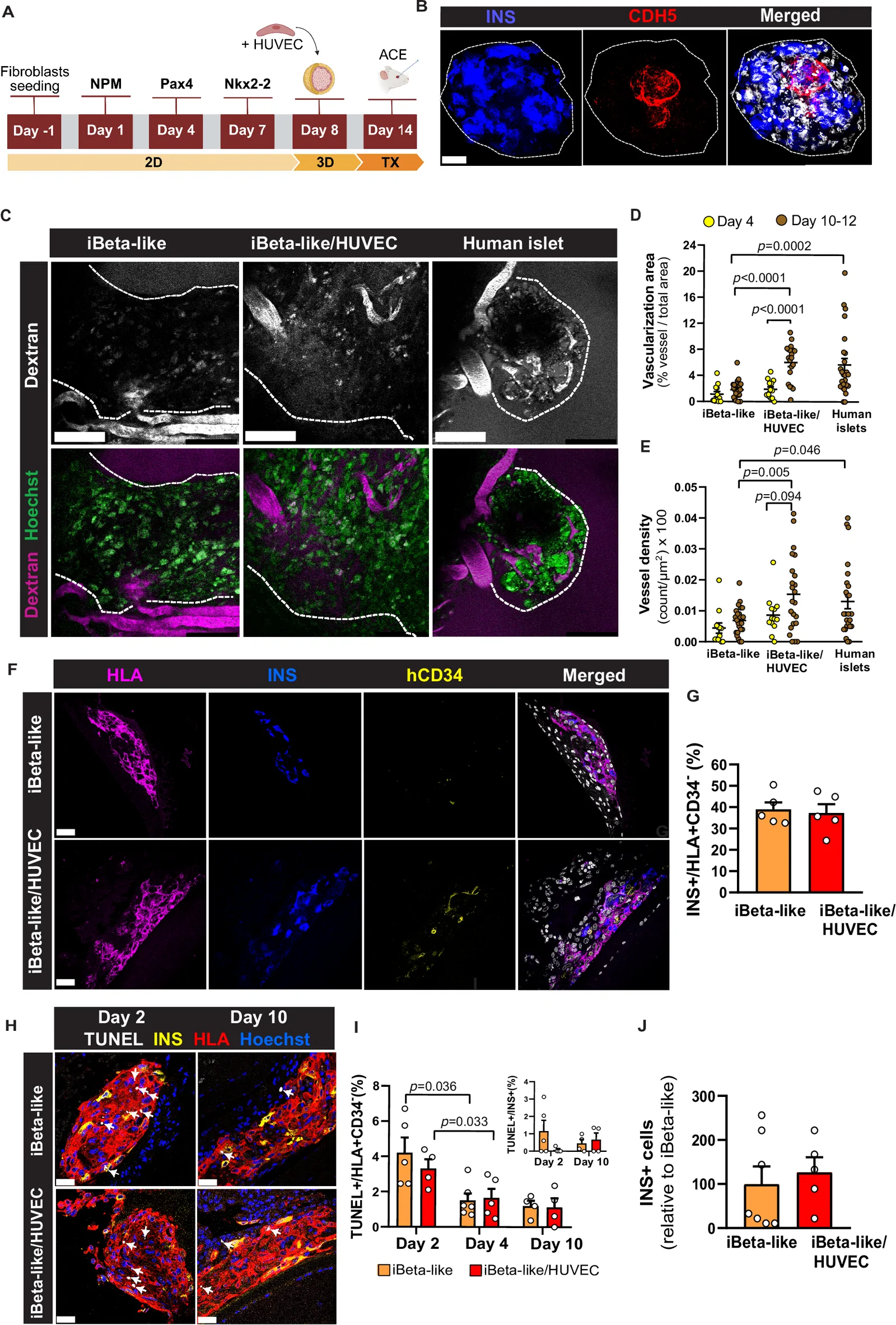
Vascularization and histological characterization of iBeta-like and iBeta-like/HUVEC grafts. (**A**) Schematic diagram of the direct reprogramming protocol used to generate iBeta-like/HUVEC cell spheroids. At day 14, spheroids (with or without HUVEC) were transplanted into the ACE of NSG mice. (**B**) Representative confocal images of an iBeta-like/HUVEC spheroid stained for insulin (INS, blue), VE-cadherin (CDH5, red) and Hoechst (white). The discontinued line represents the spheroid border. Scale bar: 25 μm. (**C**) Representative in vivo two-photon microscopy images illustrating functional vasculature labeled with RITC-dextran (white in top row, magenta in bottom row) and transplanted cells visualized via Hoechst-stained nuclei (green, bottom row) in iBeta-like and iBeta-like/HUVEC grafts at day 10 post-transplantation, and in human islet grafts at day 12 post-transplantation. Discontinued lines represent graft borders. Scale bars: 100 μm. (**D**,** E**) Quantitative analysis of vascularized area (**D**) and vessel density (**E**) in the indicated graft types and times after transplantation. (**F**) Representative immunofluorescence images of the indicated graft types stained for HLA (magenta; human cells), human insulin (INS, blue), human CD34 (hCD34, yellow) and Hoechst (white; nuclei) at day 10 post-transplantation. Scale bar: 25 μm. (**G**) Reprogramming rates at day 10 post-transplantation, expressed as the percentage of INS^+^ cells relative to the total number of human (HLA^+^) non-endothelial (CD34^-^) cells. (**H**) Immunofluorescence images of the indicated graft types stained for TUNEL (white), HLA (red), human insulin (INS, yellow) and Hoechst (blue; nuclei) at days 2 and 10 post-transplantation. White arrows indicate TUNEL^+^ cells. Scale bar: 25 μm. (**I**) Quantification of cell death using the TUNEL assay at the indicated days post-transplantation. Data in the main graph show the percentage of TUNEL^+^ cells relative to the total transplanted cell population, excluding HUVECs. The inset shows the percentage of TUNEL^+^ cells among INS^+^ cells. (**J**) Quantification of INS^+^ cells in the indicated graft types at day 30 post-transplantation. Data were expressed relative to iBeta-like cells, with values set to 100%. Data were shown as mean ± SEM, with the number of observations indicated by individual dots. In panels (**D**, **E**), each dot represents one measured area, with data collected from *n* = 4–6 grafts from each cell type. In panels (**G**, **H**, **I**), each dot represents the mean value for a single graft. Statistical significance was assessed using two-way ANOVA in panels  (**D**, **E**, **I**) for comparisons between iBeta-like and iBeta-like/HUVEC grafts across the different days analysed; one-way ANOVA for comparisons between graft types at days 10–12 (panels **D**, **E**); and unpaired Student’s *t*-tests for panels (**G**, **J**). Source data are available online for this figure.

**Figure EV1:**
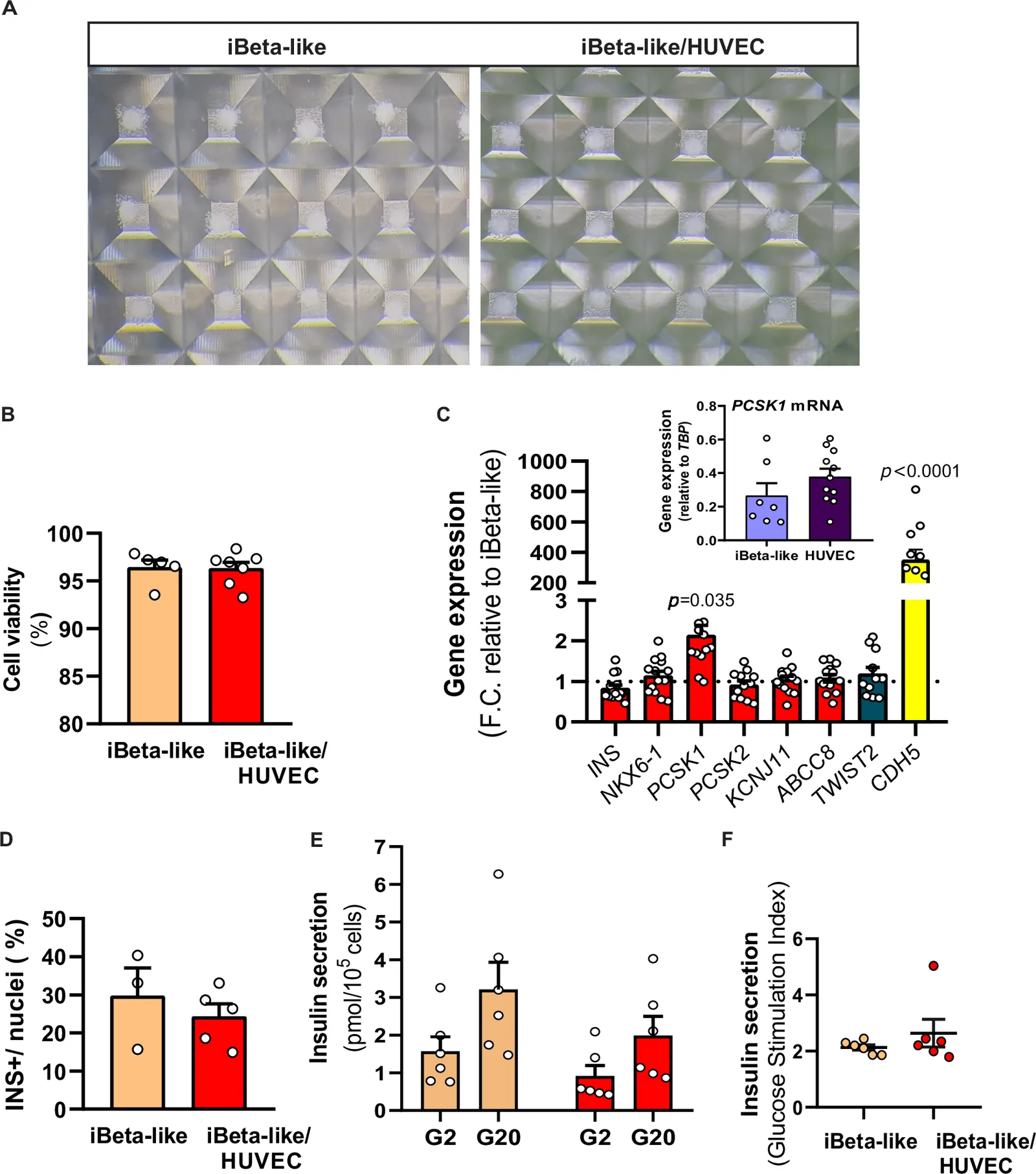
Characterization of iBeta-like and iBeta-like/HUVEC spheroids. (**A**) Photographs of AggreWell plates showing spheroids composed of iBeta-like or iBeta-like/HUVEC cells 5 days after seeding single cells into microwells. (**B**) Cell viability as assessed by CFDA and propidium iodide (PI) staining. Cell viability was calculated as the percentage of CFDA^+^PI^-^ cells relative to the total number of cells. Each dot represents an individual spheroid. (**C**) Gene expression analysis of the indicated genes by qPCR. Data in the main graph are presented as fold change relative to iBeta-like spheroids, which were set to 1. The inset compares *PCSK1* gene expression in iBeta-like cells and HUVECs; values were normalized to the housekeeping gene *TBP* within each sample. Each dot represents an independent spheroid preparation or HUVEC culture. (**D**) Percentage of INS^+^ cells in the indicated spheroid types at the end of the reprogramming protocol. For iBeta-like/HUVEC spheroids, CDH5^+^ cells (corresponding to HUVECs) were excluded from the total nuclei count. Each dot represents an independent reprogramming experiment. (**E**,** F**) Insulin secretion was measured in static incubation assays. Secreted insulin at 2 mM (G2) and 20 mM (G20) glucose is shown in Panel (**E**). The glucose stimulation index, representing the fold change between G20 and G2, is shown in Panel (**F**). Dots represent independent spheroid pools from at least three independent reprogramming experiments. Data were presented as mean ± SEM, with n indicated by individual dots. Statistical analysis was performed using unpaired Student’s *t*-test in (**B**, **D**, **F**); one-sample *t*-test in (**C**) and two-way ANOVA in (**E**).

To better recapitulate the microenvironment of donor human islets, human umbilical vein endothelial cells (HUVECs) were incorporated into iBeta-like cells during the aggregation phase of the reprogramming protocol at a 1:9 ratio (HUVEC:iBeta-like cells) (Fig. 1A). After 6 days of culture, the resulting HUVEC-containing iBeta-like spheroids (hereafter referred to as iBeta-like/HUVEC) exhibited viability and gross morphology comparable to spheroids composed exclusively of iBeta-like cells (Fig. EV1A,B).

Immunofluorescence analysis of iBeta-like/HUVEC spheroids revealed a distinct spatial organization, with HUVECs preferentially localizing to the spheroid core in a ring-like arrangement, while insulin-positive (INS^+^) cells were predominantly found at the periphery (Fig. 1B). Gene expression analysis demonstrated comparable levels of beta cell and fibroblast-associated markers between iBeta-like and iBeta-like/HUVEC spheroids, with the exception of a significant upregulation of the prohormone convertase gene *PCSK1* in iBeta-like/HUVEC spheroids (Fig. EV1C). Notably, *PCSK1* mRNA was readily detected in HUVECs, which may account for the observed increase in *PCSK1* expression in iBeta-like/HUVEC spheroids (Fig. EV1C). Quantification of INS^+^ cells revealed no significant differences between both spheroid types (Fig. EV1D). Moreover, functional assessment by glucose-stimulated insulin secretion showed comparable responses between the two groups (Fig. EV1E,F). Collectively, these data indicate that the inclusion of 10% HUVECs during the 3D reprogramming stage did not markedly affect the efficiency of in vitro fibroblast conversion into iBeta-like cells.

We next evaluated the extent of vascularization in iBeta-like and iBeta-like/HUVEC grafts in comparison with isolated human donor islets. Transplantations were performed into the anterior chamber of the eye (ACE) of normoglycemic NSG mice, a site that enables longitudinal in vivo imaging and direct assessment of functional vascular integration. Vascularization was analysed at 4 and 10 days post-transplantation for iBeta-like grafts and at day 12 for human islets using RITC-dextran, a fluorescent blood tracer that allows real-time visualization of perfused vessels within the grafts. Although no significant differences were detected at day 4, by day 10, grafts generated from iBeta-like/HUVEC spheroids displayed a significantly higher fractional vascularization and vessel density compared with grafts composed of iBeta-like spheroids alone (Fig. 1C–E). Notably, the extent of vascularization approached that observed in transplanted human donor islets (Fig. 1C–E). These findings indicate that the inclusion of HUVECs enhances in vivo vascularization of iBeta-like grafts.

Histological analysis of the grafts revealed a similar percentage of INS^+^ cells among HLA^+^CD34^-^ ( = human, non-endothelial) cells, revealing no changes in reprogramming rates between the two groups (Fig. 1F,G). To evaluate the impact of enhanced vascularization on post-transplantation cell survival, TUNEL staining was conducted on days 2, 4, and 10. Both iBeta-like and iBeta-like/HUVEC spheroids showed a comparable reduction in cell death from day 2 to day 4, which remained stable through day 10. This temporal reduction was not evident in INS^+^ cells, and no significant differences were detected between graft types (Fig. 1H,I). These results suggest that the presence of HUVECs does not markedly affect graft survival during the early post-transplantation period, nor at later stages when differences in vascularization become significant. Additionally, the proportion of proliferating INS^+^ cells remained low and varied across grafts, but did not differ significantly between groups at either day 4 or day 10 post-transplantation (Appendix Fig. S1). Consistent with prior findings in control grafts (Fontcuberta-PiSunyer et al, 2023), long-term evaluation up to 30 days post-engraftment showed a low number of remaining INS^+^ cells, with no significant differences observed between both types of grafts (Fig. 1J).

Overall, these results demonstrate that the addition of HUVECs positively impacts graft vascularization, as evidenced by an enhanced vascular network compared to controls. Despite this improved vascularization, there is no evident impact on the persistence or expansion of reprogrammed cells over time.

### Effects of NaW on human iBeta-like/HUVEC grafts

Given that the addition of endothelial cells alone did not markedly improve graft survival, we investigated whether combining this approach with the PTP1B inhibitor NaW could enhance outcomes, based on our previous findings showing beneficial effects of PTP1B deletion in transplanted islets (Figueiredo et al, 2019). To test this hypothesis, we conducted a new series of transplantation experiments using iBeta-like/HUVEC spheroids. Post-transplantation, recipient animals were divided into two groups: one received no treatment (control), while the other was given NaW at a concentration of 1 g/L in their drinking water. Consistent with previous studies, no significant differences in body weight or glycemia were observed over the 30-day follow-up period (Appendix Fig. S2).

**Figure 2:**
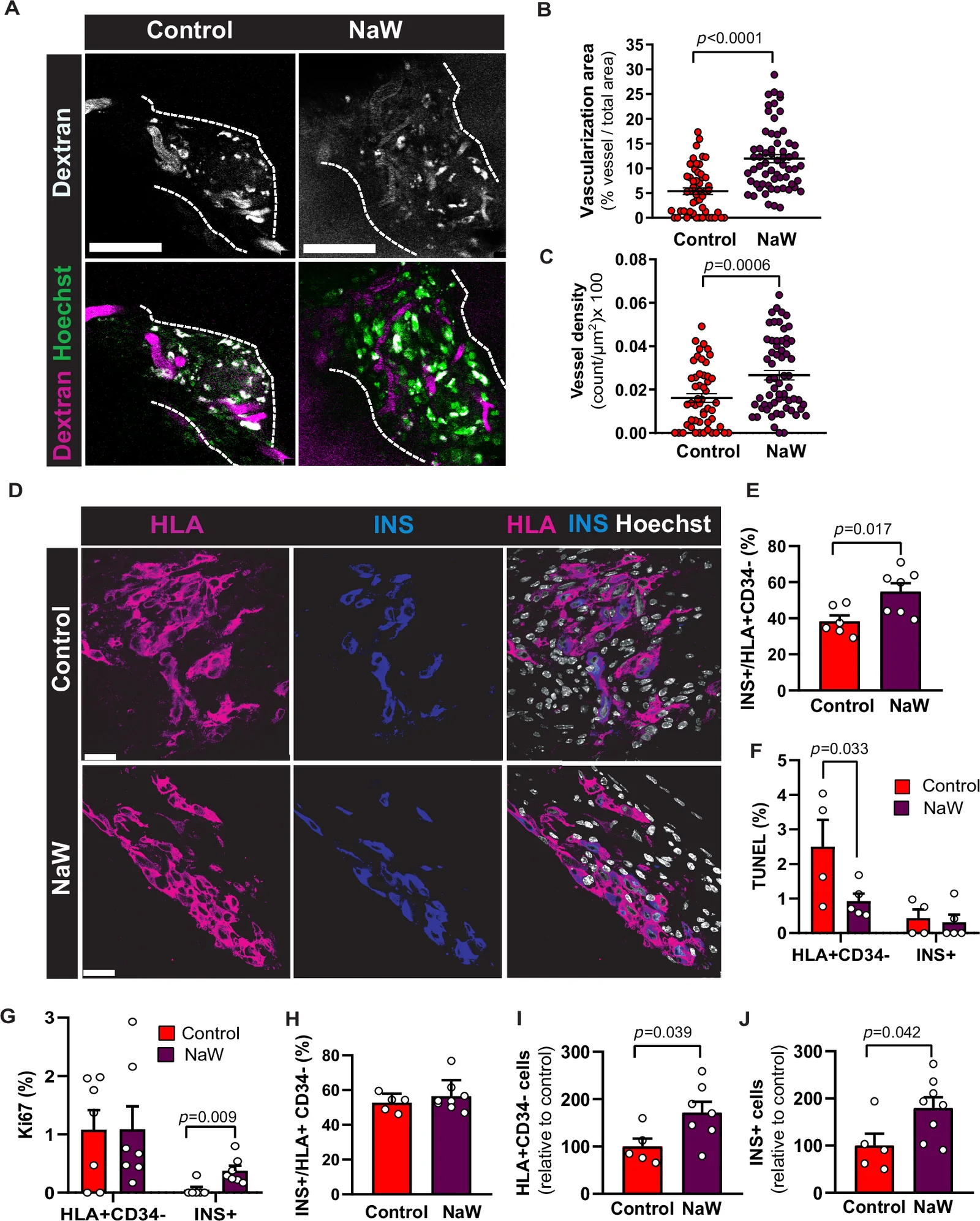
Effects of NaW treatment on iBeta-like/HUVEC grafts. (**A**) Representative in vivo two-photon microscopy images of iBeta-like/HUVEC grafts illustrating functional vasculature labeled with RITC-dextran (white in top row, magenta in bottom row) and transplanted cells visualized via Hoechst-stained nuclei (green, bottom row) in control and NaW-treated mice at day 10 post-transplantation. White fluorescence observed in some cells, unrelated to vascular structures, corresponds to residual Cherry^+^ expression from the reprogramming adenovirus. Discontinued lines represent graft borders. Scale bars: 100 μm. (**B**,** C**) Quantitative analysis of vascularized area (**B**) and vessel density (**C**) in grafts at day 10 post-transplantation. (**D**) Representative immunofluorescence images of iBeta-like/HUVEC grafts stained for HLA (magenta; human cells), human insulin (INS, blue) and Hoechst (white; nuclei) at day 10 post-transplantation in control and NaW-treated mice. Scale bar: 25 μm. (**E**) Reprogramming rates at day 10 post-transplantation, expressed as the percentage of INS^+^ cells relative to the total number of human (HLA^+^) non-endothelial (CD34^−^) cells. (**F**, **G**) Quantification of cell death by TUNEL (**F**) and cell proliferation by Ki67 immunostaining (**G**) in grafts at day 10. Percentages are shown for HLA^+^CD34^-^ cells and for INS^+^ cells, relative to total HLA^+^CD34^-^ and total INS^+^ cells, respectively. (**H**) Reprogramming rates at day 30 post-transplantation, expressed as the percentage of INS^+^ cells relative to the total number of human (HLA^+^) non-endothelial (CD34^-^) cells. (**I**,** J**) Quantification of HLA^+^CD34^-^ cells (**I**) and INS^+^ cells (**J**) in grafts from control and NaW-treated mice at day 30 post-transplantation. Values are expressed relative to control grafts, with values set to 100%. Data were presented as mean ± SEM, with the number of observations indicated by individual dots. Each dot represents the mean value from a single graft, except in panels (**B**, **C**), where each dot corresponds to one measured area from a total of 10–12 grafts. Statistical significance was determined using an unpaired Student’s *t*-test. Source data are available online for this figure.

**Figure EV2:**
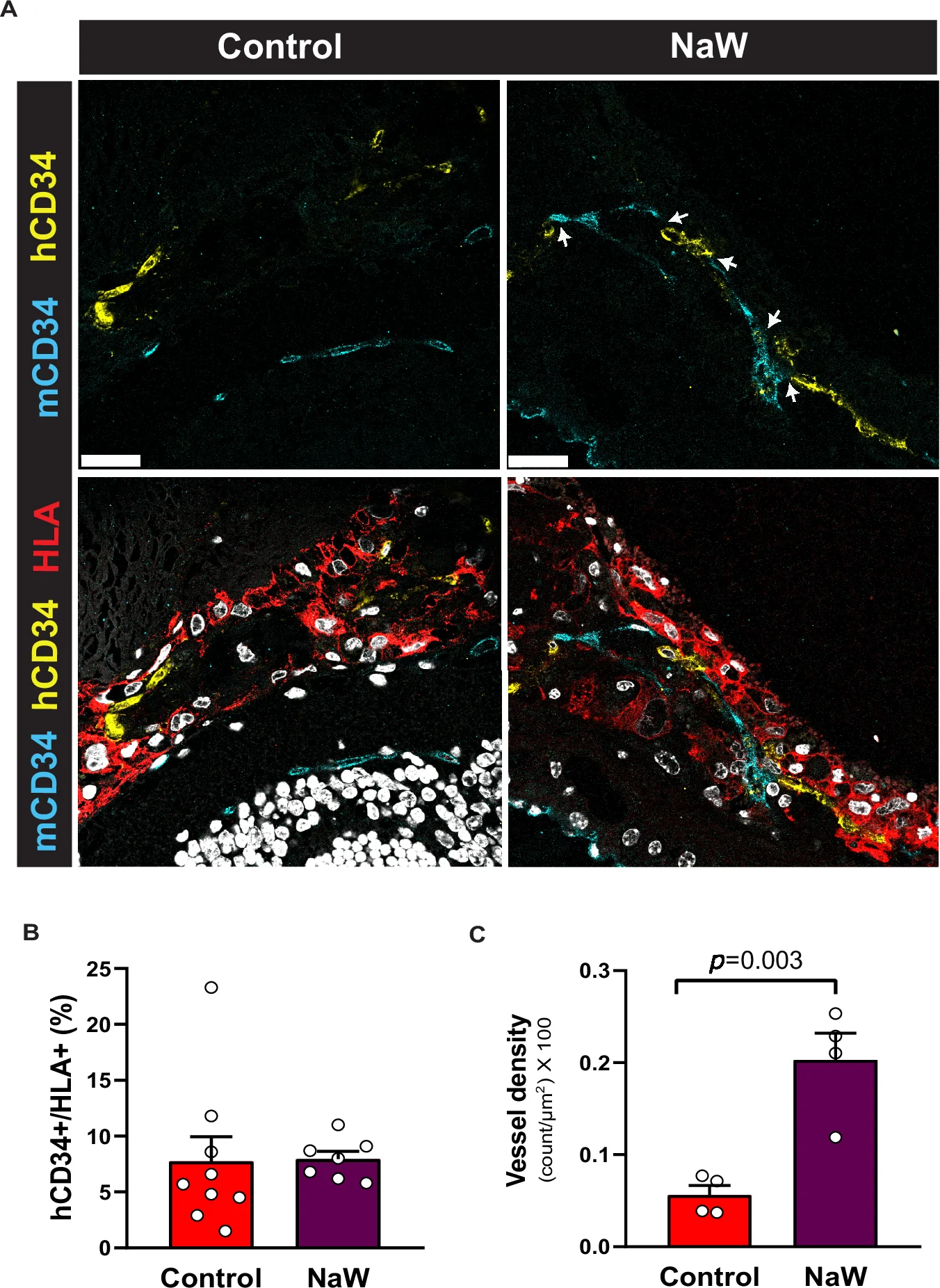
Histological characterization of HUVEC and mouse ECs in iBeta-like/HUVEC grafts at day 10 post-transplantation. (**A**) Representative immunofluorescence images of iBeta-like/HUVEC grafts at day 10 post-transplantation in control and NaW-treated mice. Grafts were stained for mouse CD34 (mCD34, cyan; mouse recipient endothelial cells), human CD34 (hCD34, yellow; transplanted HUVECs), HLA (red; transplanted human cells), and Hoechst (white; nuclei). White arrows indicate regions where mouse ECs are adjacent to human ECs. Scale bar: 25 μm. (**B**) Percentage of hCD34^+^HLA^+^ cells relative to total HLA^+^ cells at day 10 post-transplantation in grafts from control and NaW-treated mice. (**C**) Number of mCD34^+^ vessels relative to HLA^+^ area at day 10 post-transplantation in grafts from control and NaW-treated mice. In (**B**, **C**): data were presented as mean ± SEM, with *n* indicated by individual dots, each dot representing the mean value from an individual graft. Statistical analysis was performed using an unpaired Student’s *t*-test.

Functional vascularization was evaluated on day 10 post-transplantation. In NaW-treated mice, the vascularization area was increased twofold compared to untreated mice (Fig. 2A,B), indicating a larger area occupied by vessels. Additionally, vessel density was elevated by 50% in the NaW group (Fig. 2A,C), representing a richer microvascular network within the graft. Histological analysis revealed the presence of both host-derived mouse ECs and transplanted HUVEC cells within the grafts of NaW-treated animals. In contrast, in control animals, mouse ECs were found only in the vicinity of the grafts but not within them (Fig. EV2A). The proportion of HUVECs was not changed by NaW treatment (Fig. EV2B), indicating that NaW selectively promotes the recruitment of host-derived ECs rather than acting on the transplanted HUVEC cells. These findings highlight NaW as a driver of host endothelial cells to the transplantation site. To confirm this, vessel density was quantified in retrieved grafts using the mouse-specific CD34 antibody. This analysis validated the significant increase in vascularization observed in vivo in NaW-treated mice (Fig. EV2C). To determine whether this vascular response was accompanied by inflammation, macrophage infiltration was evaluated using F4/80 staining. While macrophages were readily visible within and around the graft region, the abundance of macrophages located in or in direct contact with the graft cells was not significantly changed by NaW (Appendix Fig. S3), suggesting that the pro-angiogenic effect of NaW is not primarily driven by an inflammatory mechanism.

We next examined how NaW treatment influenced iBeta-like cell graft outcomes. NaW-treated animals exhibited nearly a 50% increase in the proportion of INS^+^ cells among total HLA^+^CD34^-^ cells (Fig. 2D,E). TUNEL assays showed a significant reduction in cell death within the HLA^+^CD34^-^ compartment in grafts from treated mice compared to controls (Fig. 2F). The percentage of INS^+^TUNEL^+^ cells were low in both groups, suggesting that the survival benefit conferred by NaW primarily affects non or partially reprogrammed cells. Overall proliferation rates within the HLA^+^CD34^−^ population were low (~1%) and were not altered by NaW treatment. In contrast, INS^+^ cells in control grafts displayed almost no proliferation, whereas NaW-treated mice showed a significant, although still low (<0.5%), increase in INS^+^ cell proliferation (Fig. 2G). Together, these findings indicate that the higher proportion of INS^+^ cells observed at day 10 in NaW-treated mice is more likely due to increased proliferation of reprogrammed cells rather than reduced cell death.

Finally, to evaluate the longer-term impact of NaW on graft outcomes, selected transplants were analysed up to 30 days post-transplantation. Grafts from NaW-treated animals displayed reprogramming rates comparable to controls at day 30 (Fig. 2H), remaining close to the ~60% level already observed at day 10. Notably, while the reprogramming rate in untreated animals increased between days 10 and 30, grafts in NaW-treated mice had already reached a higher level at day 10 and maintained it thereafter, indicating that the effects of NaW occur early after transplantation. Moreover, by day 30, grafts from treated mice exhibited a twofold increase in HLA^+^CD34^+^ and INS^+^ cell numbers compared with controls (Fig. 2I,J), consistent with improved long-term survival of transplanted cells. Together, these findings highlight NaW as an effective, early acting enhancer of graft preservation and stability.

### Effects of NaW on VEGFA production by human iBeta-like cells

**Figure 3:**
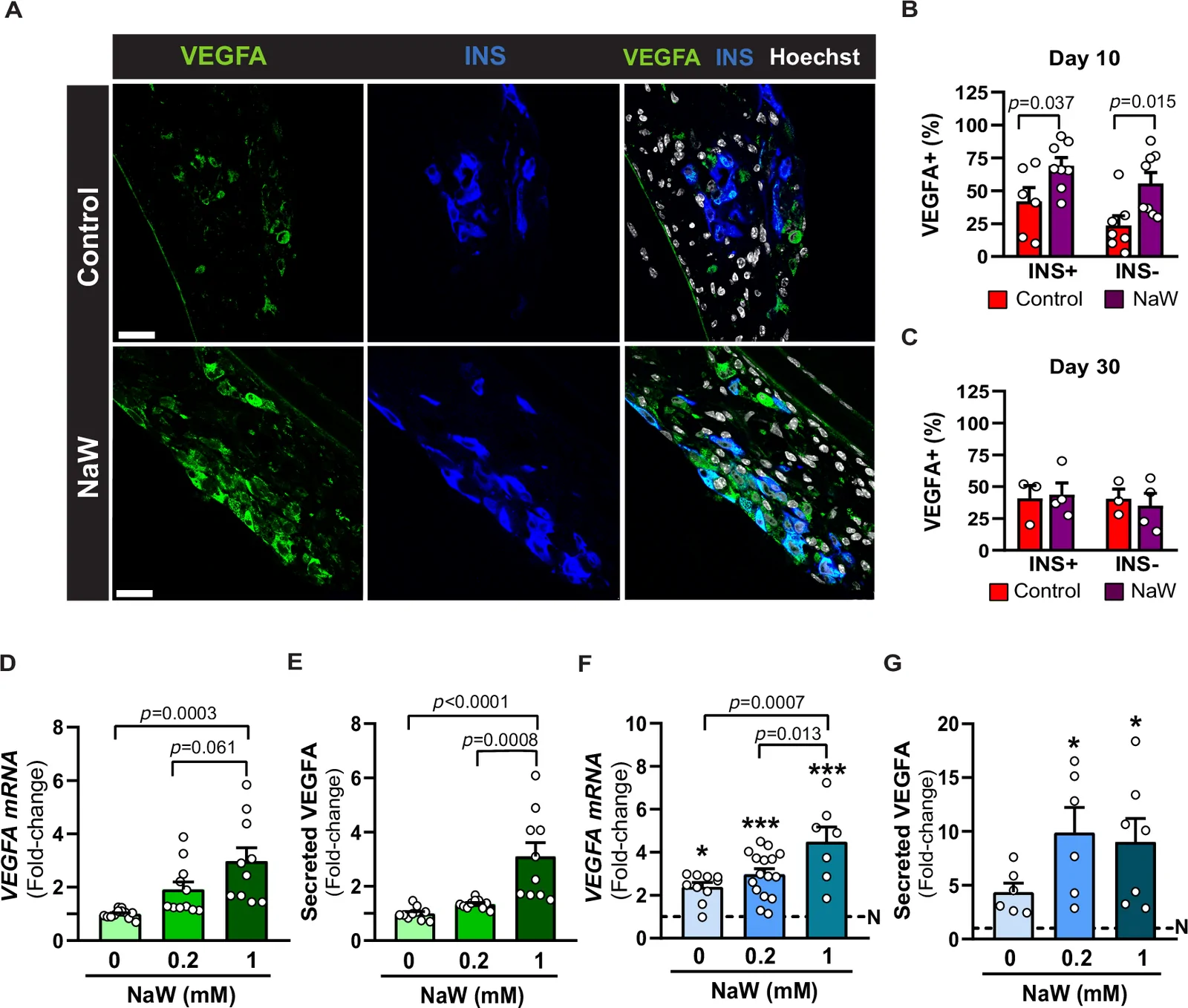
Analysis of VEGFA production by iBeta-like cells in vivo and in vitro. (**A**) Representative immunofluorescence images of iBeta-like/HUVEC grafts at day 10 post-transplantation from control and NaW-treated mice. Grafts were stained for VEGFA (green), insulin (INS, blue), and Hoechst (white; nuclei). Scale bar: 25 μm. (**B**,** C**) Quantification of the percentage of INS^+^ and INS^-^ cells co-expressing VEGFA relative to total HLA^+^CD34^−^ cells in iBeta-like/HUVEC grafts at day 10 (**B**) and day 30 (**C**) post-transplantation from control and NaW-treated mice. (**D**,** E**) *VEGFA* mRNA expression (**D**) and VEGFA protein released to the culture media (**E**) after culturing iBeta-like cells with or without NaW at the indicated concentrations for 48 h. Expression was measured by qPCR using *TBP* as a housekeeping gene and the amount of secreted VEGFA protein was quantified by ELISA. Data were expressed as fold change relative to untreated cells. (**F**,** G**) *VEGFA* mRNA expression (**F**) and VEGFA protein released to the culture media (**G**) after culturing iBeta-like cells with or without NaW at the indicated concentrations for 24 h under hypoxic conditions. Expression was measured by qPCR using *TBP* as a housekeeping gene and the amount of secreted VEGFA protein was quantified by ELISA. Data were expressed as fold change relative to untreated normoxic control (N, dotted line). Data were presented as mean ± SEM, with individual dots indicating the number of observations. In panels (**B**, **C**), each dot corresponds to a single graft. For all other panels, values are derived from at least three independent reprogramming experiments with 2–3 replicates each. Statistical significance was assessed using unpaired Student’s *t*-test between indicated groups (**B**, **C**) or one-way ANOVA (**D**–**G**). In (**F**, **G**): *p < 0.05, ***p < 0.0001 relative to normoxia (N). Exact *p* values were: (**F**) control, *p* = 0.019; 0.2 mM and 1 mM NaW, *p* < 0.0001; (**G**) 0.2 mM NaW, *p* = 0.016; 1 mM NaW, *p* = 0.027. Source data are available online for this figure.

In our previous study (Figueiredo et al, 2019), we demonstrated that transplanted beta cells deficient in the phosphatase PTP1B produce higher levels of VEGFA, which was proposed as the mechanism responsible for enhanced graft vascularization. To determine whether the pro-vascularization effect induced by NaW in iBeta-like cell grafts was mediated by upregulation of VEGFA, we performed immunofluorescence analysis on grafts collected 10 days post-transplantation. Quantitative analysis showed that 69% of INS^+^ cells in grafts from NaW-treated mice co-expressed VEGFA, compared with 43% in grafts from control animals (Fig. 3A,B). Increased VEGFA expression was also observed in insulin-negative cells, with 55% of VEGFA^+^/INS^-^ cells in grafts from treated mice versus 23% in control grafts.

By day 30 post-transplantation, the proportions of both VEGFA^+^/INS^+^ and VEGFA^+^/INS^-^ cells were comparable between treatment groups (Fig. 3C), indicating that the effect of NaW on VEGFA expression is transient. Together, these results suggest that NaW temporarily enhances VEGFA production in transplanted cells, a response that may be critical for promoting early graft vascularization.

To determine whether NaW directly influences VEGFA production in iBeta-like cells, we cultured the cells with or without NaW (0.2 or 1 mM) for 48 h. *VEGFA* mRNA expression was significantly increased at the highest concentration tested (Fig. 3D), and elevated levels of VEGFA protein was detected in the conditioned medium of NaW-treated cells (Fig. 3E), demonstrating that NaW enhances VEGFA production and secretion in iBeta-like cells. To mimic the transplantation microenvironment, we repeated the experiment under hypoxic conditions (3% O₂). After 48 h, *VEGFA* mRNA was increased under hypoxia compared to normoxia, and NaW (1 mM) further augmented this induction. A similar magnitude of increase was observed for secreted VEGFA protein relative to hypoxia alone; however, this increase was more variable across replicates and did not reach statistical significance. Compared to the normoxia control, secreted VEGFA under NaW was significantly elevated, whereas hypoxia alone was not (Fig. 3F,G).

Together, these findings indicate that NaW enhances VEGFA expression and secretion in iBeta-like cells, both under normoxic and hypoxic conditions, supporting VEGFA as a candidate mediator of NaW-induced vascularization in vivo.

### Direct effects of NaW on human endothelial cells

VEGFA signaling via VEGFR2 is negatively regulated by PTP1B, which dephosphorylates VEGFR2 and thereby attenuates downstream signaling (Lanahan et al, 2014). Given this regulatory relationship, we next asked whether NaW, in addition to stimulating VEGFA production by iBeta-like cells, could potentiate the paracrine effects of VEGFA on endothelial cells. To this end, HUVECs were treated with NaW in the presence or absence of exogenous VEGFA, and endothelial responses were assessed using standard functional assays.

**Figure 4:**
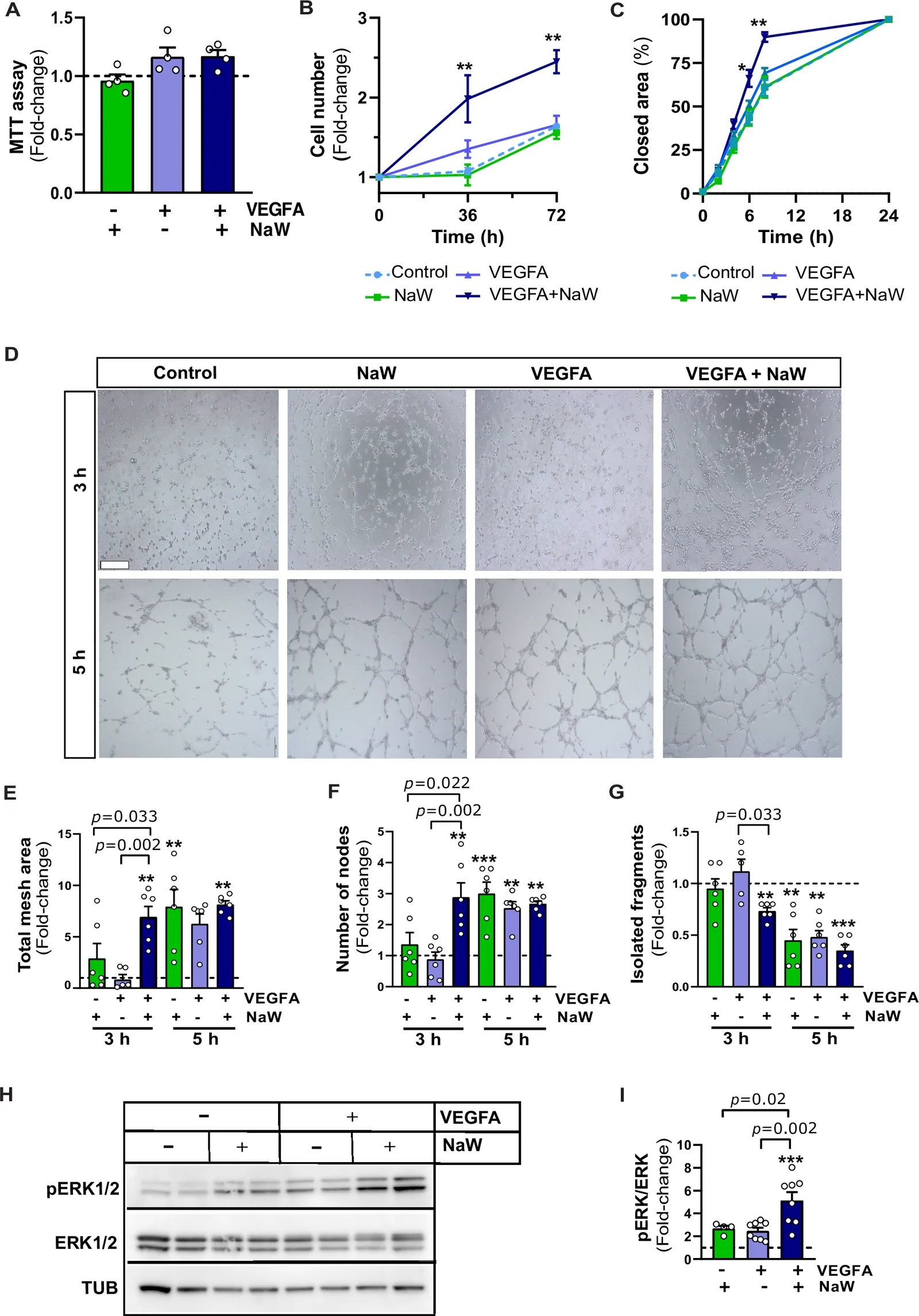
Effects of NaW on endothelial cell function in vitro. (**A**–**G**) HUVEC cells were cultured in normal medium without VEGFA and NaW (control), VEGFA (2 ng/mL), NaW (0.2 mM), or VEGFA (2 ng/mL) + NaW (0.2 mM). (**A**) HUVEC viability assessed by MTT assay after 48 h. Results are normalized to control (dotted line, set to 1). (**B**) HUVEC proliferation was evaluated by direct cell counting at the indicated time points. Data were expressed as fold increase relative to cell number at time 0 (set to 1). (**C**) HUVEC migration assessed by wound healing assay. Results are expressed relative to the 24-h time point, when wound closure reached 100% in all conditions. (**D**) Representative images of tube formation at 3 and 5 h. Scale bar: 200 μm. (**E**–**G**) Quantitative analysis of tubulogenesis at 3 and 5 h: total mesh area (**E**), number of nodes (**F**), and number of isolated segments (**G**). Data were expressed as fold increase relative to control cells (dotted line, set to 1). (**H**,** I**) Immunoblot analysis of ERK1/2 phosphorylation following 10 min of stimulation under the conditions described above. Tubulin (TUB) was used as a loading control. Panel H shows representative blots; panel I shows quantification of band intensities. Data were normalized to control (dotted line, set to 1). Data were presented as mean ± SEM for the number of *n* indicated by dots. In panel (**B**), *n* = 3–4 and in panel (**C**), *n* = 9. In panel (**I**), the data corresponds to four blots from four independent experiments. Statistical significance was determined using one-way (**A**, **E**, **F**, **G**, **I**) or two-way ANOVA (**B**, **C**). *p < 0.05, **p < 0.005, ***p < 0.0001 Exact *p* values were: (**B**) VEGFA + NaW vs control, *p* = 0.0003 (36 h), *p* = 0.0043 (72 h); VEGFA+NaW vs VEGFA, *p* = 0.007 (36 h), *p* = 0.0006 (72 h); (**C**) VEGFA+NaW vs control, *p* = 0.008 (6 h), *p* = 0.0006 (8 h); VEGFA + NaW vs VEGFA, *p* = 0.0005 (8 h); (**E**) Compared to control: VEGFA + NaW, *p* = 0.001 (3 h), *p* = 0.0003 (5 h); NaW, *p* = 0.0004 (5 h); VEGFA, *p* = 0.006 (5 h); (**F**) Compared to control: VEGFA + NaW, *p* = 0.004 (3 h), *p* = 0.0002 (5 h); NaW, *p* < 0.0001 (5 h); VEGFA *p* = 0.0005 (5 h); (**G**) Compared to control: VEGFA + NaW, *p* = 0.033 (3 h), *p* < 0.0001 (5 h); NaW, *p* = 0.0002 (5 h); VEGFA, *p* = 0.0004 (5 h); (**I**) VEGFA + NaW vs control, *p* < 0.0001. Source data are available online for this figure.

Cell viability, measured by MTT assay as an indicator of mitochondrial metabolic activity, was comparable across all treatment groups (Fig. 4A), confirming that NaW was not cytotoxic under the conditions tested. In contrast, direct cell counts, which quantify actual increases in cell number, revealed a significant rise in proliferation in NaW-treated HUVECs, but only in the presence of VEGFA (Fig. 4B). These data indicate that NaW enhances VEGFA-driven proliferative signaling.

Endothelial motility was then evaluated using a wound healing assay, which monitors the capacity of cells to migrate and repopulate a defined gap in a monolayer. NaW significantly increased migration when VEGFA was present, whereas no effect was observed with NaW alone (Fig. 4C), supporting a VEGFA-dependent enhancement of motility.

To assess pro-angiogenic behavior, a tube formation assay was performed to quantify the ability of endothelial cells to assemble into capillary-like structures. A rapid and pronounced increase in tubulogenesis was detected within 3 h in the VEGFA + NaW group (Fig. 4D–G). Notably, by 5 h, NaW alone induced tube formation to a level comparable to that achieved with VEGFA alone or with the VEGFA + NaW combination. As HUVECs are known to produce VEGFA (Imaizumi et al, 2000), this delayed effect of NaW alone may reflect potentiation of endogenous VEGFA autocrine signaling rather than a VEGFA-independent action.

To further define the signaling pathways involved, we examined activation of the MAPK/ERK1/2 cascade, a central mediator of VEGFA-driven endothelial responses. ERK1/2 phosphorylation was assessed by immunoblotting. Whereas VEGFA or NaW alone induced only modest ERK1/2 activation, cotreatment resulted in a pronounced increase in phosphorylation (Fig. 4H, I), indicative of a synergistic effect. Given ERK1/2’s well-established role in promoting endothelial proliferation, migration, and tubulogenesis, these results suggest that NaW enhances VEGFA-induced angiogenic behavior by potentiating MAPK/ERK signaling.

Collectively, these findings indicate that the pro-angiogenic actions of NaW arise not only from its ability to stimulate VEGFA production by iBeta-like cells, but also from its capacity to amplify VEGFA-dependent endothelial responses. This dual mechanism likely contributes to improved graft vascularization and may enhance the therapeutic efficacy of NaW in transplantation settings.

### Effects of NaW on human SC-islet grafts

To increase the translational relevance of these findings, we extended our experimental paradigm to human embryonic stem cell-derived islets (SC-islets), a clinically validated cell source (Reichman et al, 2025) (Appendix Fig. S4). Similar to primary human islets, SC-islet engraftment is often hindered by inadequate vascularization. By evaluating NaW in this context, we aimed to further validate its pro-vascular effects using a cell model currently employed in clinical protocols.

SC-islets were transplanted into the ACE of normoglycemic NSG mice, and in vivo vascularization was assessed at days 15 and 30 post-transplantation. Throughout the study, no differences in body weight or glycemia were observed between groups (Appendix Fig. S5), indicating that neither the transplantation procedure nor NaW treatment affected systemic metabolic parameters.

**Figure 5:**
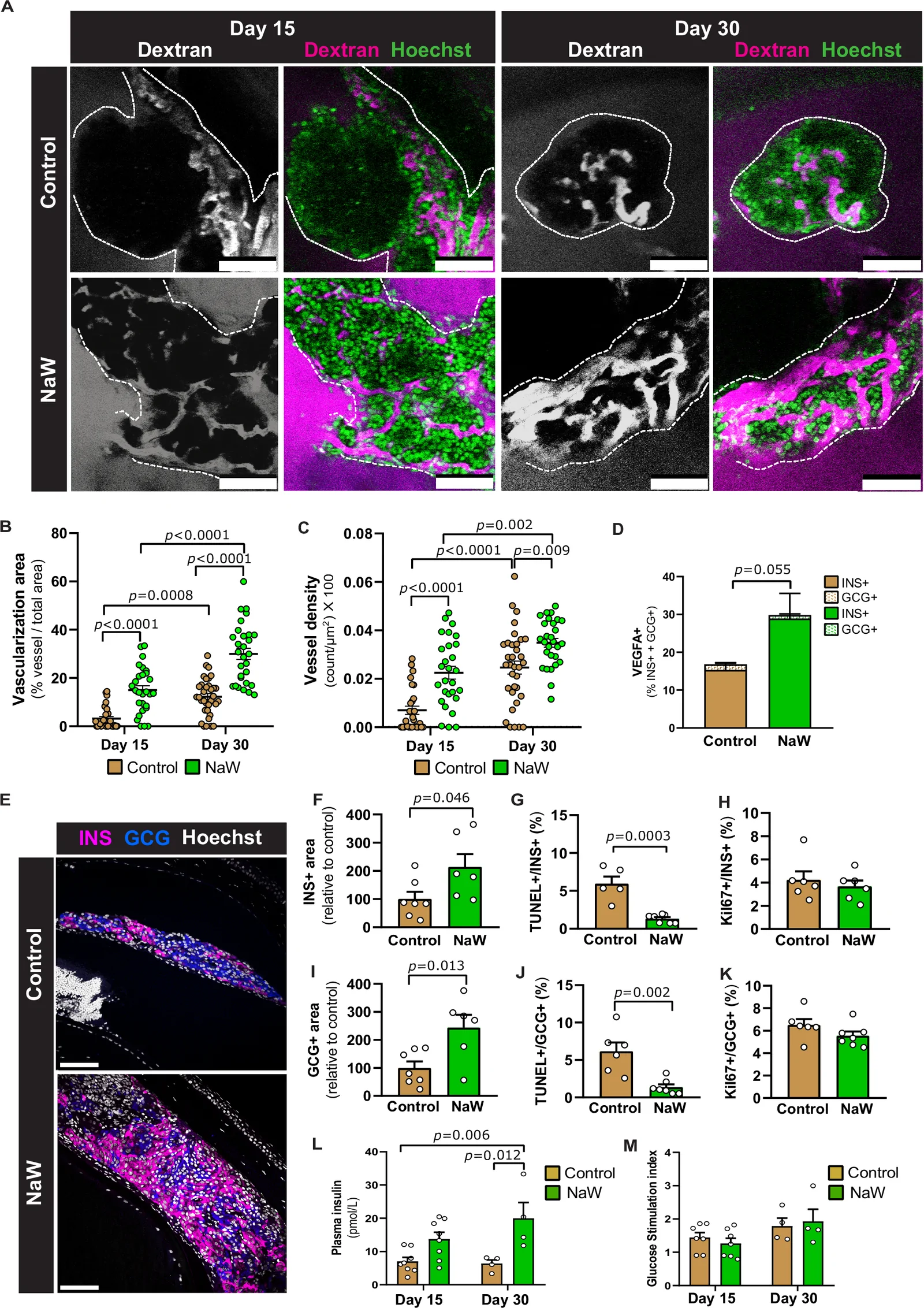
Effects of NaW treatment on SC-islet grafts. (**A**) Representative in vivo two-photon microscopy images of SC-islet grafts illustrating functional vasculature labeled with RITC-dextran (white in top row, magenta in bottom row) and transplanted cells visualized via Hoechst-stained nuclei (green, bottom row) in control and NaW-treated mice at day 15 post-transplantation. Discontinued lines represent graft borders. Scale bars: 100 μm. (**B**,** C**) Quantitative analysis of vascularized area (**B**) and vessel density (**C**) in grafts at days 15 and 30 post-transplantation. (**D**) Quantitative analysis of the percentage of endocrine cells (including INS^+^ and GCG^+^ cells) co-expressing VEGFA in grafts at day 5 post-transplantation from control and NaW-treated mice. (**E**) Representative immunofluorescence images of grafts stained for insulin (INS, magenta), glucagon (GCG, blue) and Hoechst (white; nuclei) in control and NaW-treated mice at day 30. Scale bar: 75 μm. (**F**,** I**) Quantification of insulin-positive (**F**) and glucagon-positive (**I**) areas in grafts from control and NaW-treated mice at day 30 post-transplantation. Values are expressed relative to control grafts, with values set to 100%. (**G**,** J**) Quantification of cell death by TUNEL. Values represent the percentage of TUNEL^+^INS^+^ (**G**) and TUNEL^+^GCG^+^ (**J**) cells relative to the total counted INS^+^ and GCG^+^ cells, respectively, in the indicated type of grafts at day 15 post-transplantation. (**H**,** K**) Quantification of cell proliferation by Ki67 staining. Values represent the percentage of Ki67^+^INS^+^ (**H**) and Ki67^+^GCG^+^ (**K**) cells relative to the total counted INS^+^ and GCG^+^ cells, respectively, in the indicated type of grafts at day 15 post-transplantation. (**L**) Fasting human plasma insulin in control mice and mice treated with NaW at days 15 and 30 post-transplantation. (**M**) Glucose-stimulated insulin secretion at days 15 and 30 post-transplantation in control and NaW-treated mice. Values are expressed as the fold increase  in plasma insulin 20 min after intraperitoneal glucose administration relative to initial fasting insulin levels for each animal (glucose stimulation index). Data were presented as mean ± SEM. The number of observations is indicated by individual dots, except for panel (**D**) (*n* = 4 grafts per condition). In panels (**B**, **C**), each dot represents one measured area from a total of *n* = 6–9 grafts. In panels (**F**–**K**), dots correspond to the mean value from individual grafts, while in panels (**L**, **M**), each dot represents an individual mouse. Statistical significance was assessed using one-tailed unpaired Student’s *t*-test (**D**), two-tailed unpaired Student’s *t*-test (**F**–**K**), or two-way ANOVA (**B**, **C**, **L**, **M**). Source data are available online for this figure.

**Figure EV3:**
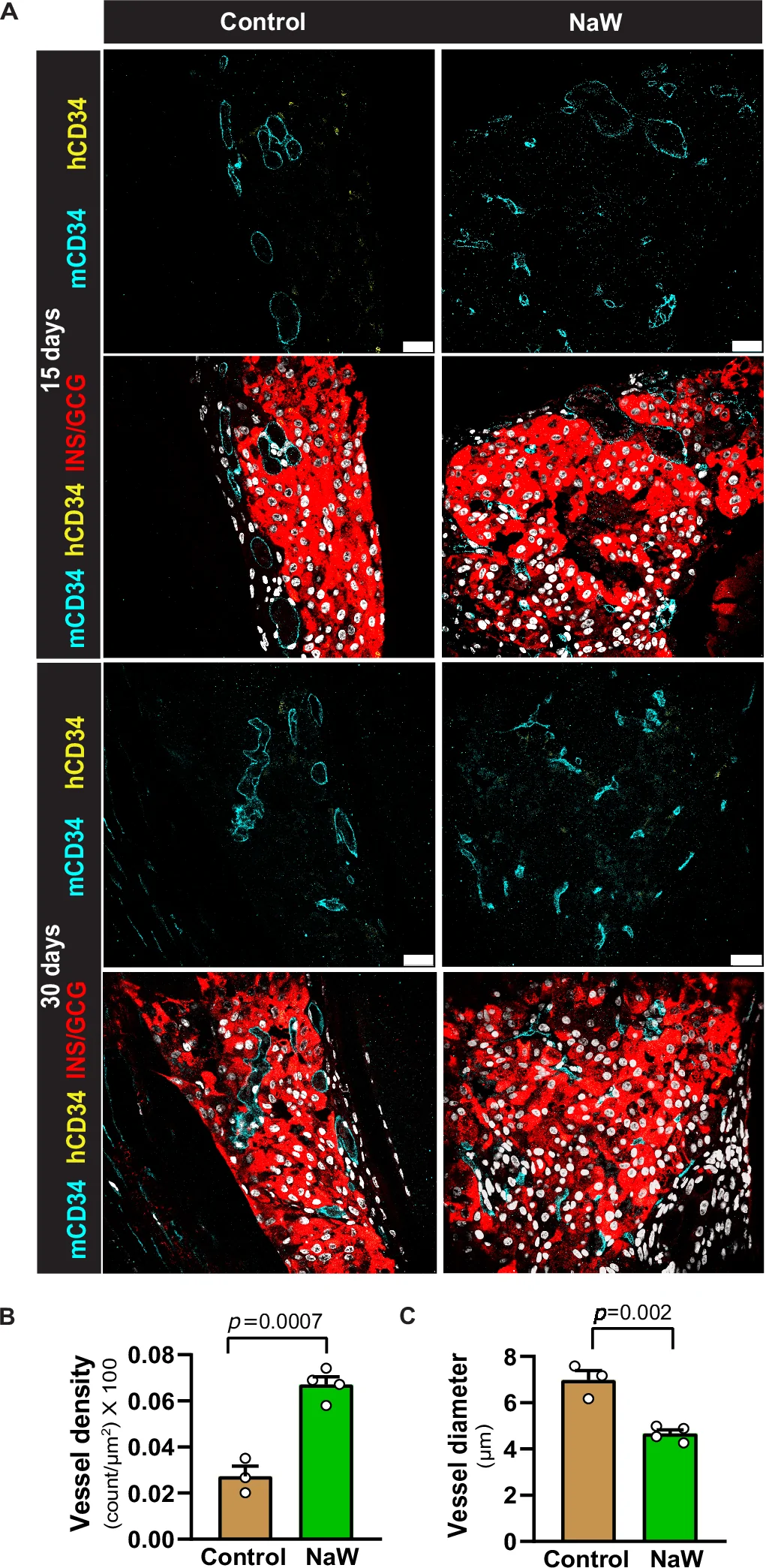
Histological characterization of human and mouse ECs in SC-islet grafts at days 15 and 30 post-transplantation. (**A**) Representative immunofluorescence images of SC-islet grafts at days 15 and 30 post-transplantation in control and NaW-treated mice. Grafts were stained for mouse CD34 (mCD34, cyan; mouse recipient endothelial cells), human CD34 (hCD34, yellow; human endothelial cells), INS + GCG (red; endocrine cells), and Hoechst (white; nuclei). Scale bar: 25 μm. (**B**) Number of mCD34^+^ vessels relative to HLA^+^ area at day 10 post-transplantation in grafts from control and NaW-treated mice. (**C**) Average lumen diameter of mCD34^+^ vessels at day 10 post-transplantation in grafts from control and NaW-treated mice. In (**B**, **C**): data were presented as mean ± SEM, with n indicated by individual dots; each dot represents the mean value from an individual graft. Statistical analysis was performed using an unpaired Student’s *t*-test.

At day 15, grafts from NaW-treated mice displayed a significantly greater vascularized area and higher vessel density compared with controls, and these differences remained robust at day 30 (Fig. 5A–C). Vascular structures appeared to originate exclusively from recipient mouse endothelial cells, as CD34 immunostaining did not detect human CD34^+^ cells within the grafts (Fig. EV3A). This observation is consistent with prior reports (Thorngren et al, 2024) and further supports the notion that host endothelial cells are the primary contributors to SC-islet graft vascularization under the conditions examined. We validated the in vivo vascularization results by immunostaining retrieved grafts with a CD34 antibody. Quantification of vessel density confirmed the increase observed in vivo, while lumen diameter measurements revealed smaller caliber structures in NaW-treated grafts (Fig. EV3B,C). This latter observation is compatible with active angiogenic remodeling, as newly formed capillaries characteristically display narrow lumina prior to maturation (Iruela-Arispe and Davis, 2009). Staining for macrophage infiltration revealed low levels that were unaffected by NaW treatment, indicating that, as observed for iBeta-like cells, the pro-vascularization effect of NaW does not appear to involve inflammatory mechanisms (Appendix Fig. S6).

To explore the mechanisms driving the enhanced vascularization, we performed VEGFA staining. At day 15, VEGFA protein was detected in only a few isolated cells in both treatment groups, prompting us to examine earlier time points. By day 5, both control and NaW-treated grafts exhibited a marked increase in VEGFA^+ ^ cells. Notably, NaW-treated grafts showed a trend toward a higher proportion of VEGFA-positive endocrine cells (combined INS⁺ + GCG⁺ cell populations) (Fig. 5D). Remarkably, the vast majority of VEGFA-expressing cells were insulin-positive, representing 91% of VEGFA^+^ cells in control grafts and 96% in NaW-treated grafts. These findings indicate that beta-like cells are the predominant source of VEGFA within SC-islet grafts at this early stage.

Quantification of the insulin-positive area at day 30 demonstrated a twofold increase in NaW-treated mice, indicating improved graft integration (Fig. 5E,F). Analysis of cell death by TUNEL staining at day 15 showed a marked reduction in INS^+^ cell apoptosis (Fig. 5G), whereas Ki67 staining revealed no significant differences in INS^+^ cell proliferation (Fig. 5H). Presence of beta cell markers, including the transcription factors PDX1, NKX6.1, and MAFA, was evaluated by immunofluorescence staining, and no differences were observed between control and NaW-treated mice, indicating that beta cell identity is preserved (Appendix Fig. S7). NaW-treated mice also showed an expansion of the glucagon-positive graft area, reduced GCG^+^ cell death, and unchanged GCG^+^ cell proliferation, suggesting that NaW effects extend beyond the beta cell compartment to support overall graft survival (Fig. 5E,I–K).

Plasma human insulin levels exhibited a trend toward higher values in NaW-treated mice at day 15 and reached statistical significance by day 30 (Fig. 5L), consistent with the increased insulin-positive graft area observed at this time point (Fig. 5F). Despite these elevations in insulin, the glucose stimulation index was similar between treatment groups at both time points (Fig. 5M), indicating that functional glucose responsiveness was preserved under all conditions. To discern a direct effect of NaW on beta cells, SC-islets were cultured for 24 h in the presence of 0.2 mM NaW. No changes were observed in *INS* and other beta cell marker gene expression, insulin protein content, or insulin secretion, indicating that NaW does not exert a direct effect on beta cell function in this in vitro system (Appendix Fig. S8). Together, these findings support a model in which NaW enhances beta cell mass and insulin output in vivo indirectly, through improved graft vascularization and survival, rather than through a direct stimulatory effect on beta cells themselves.

**Figure EV4:**
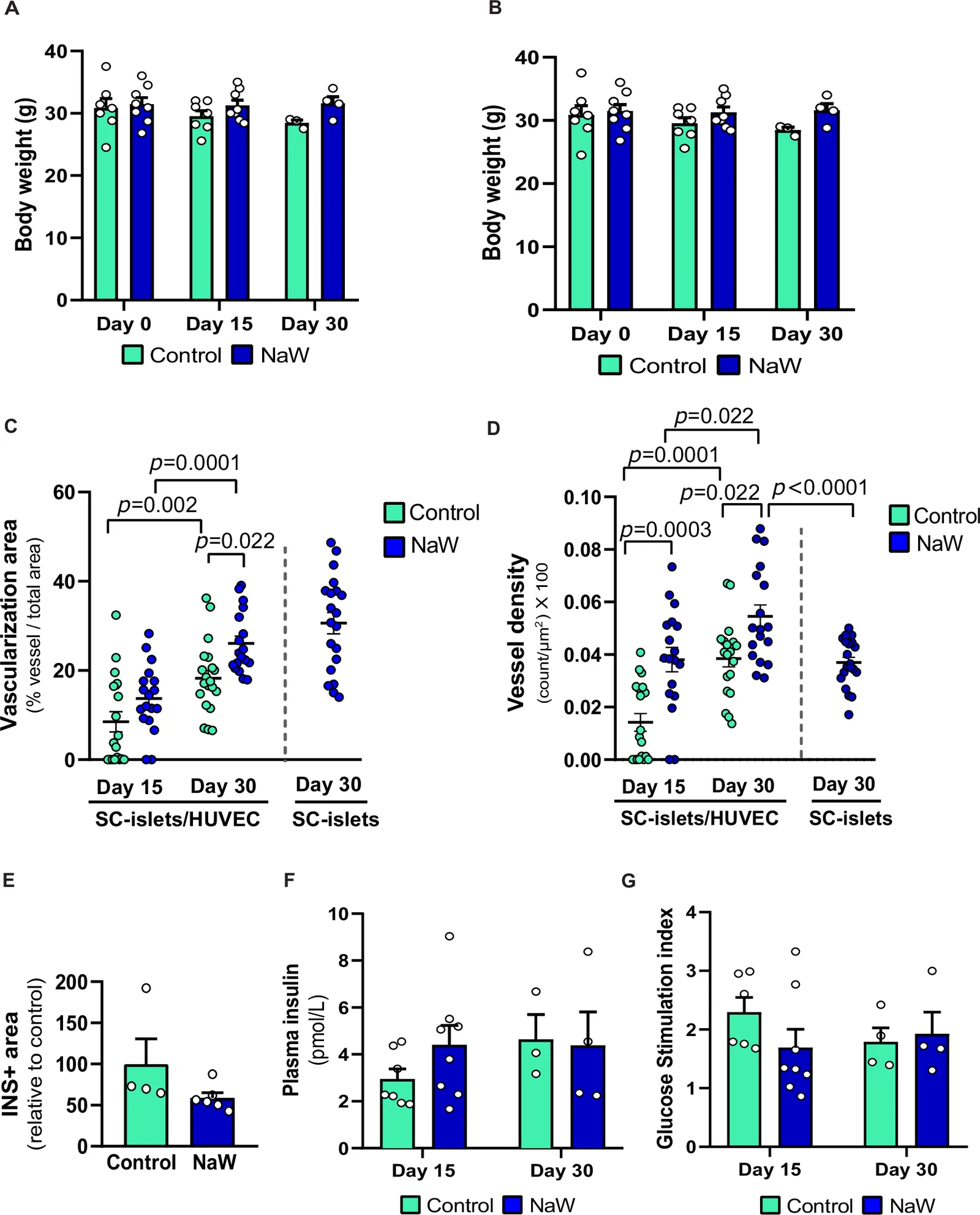
Effects of NaW treatment on SC-islet/HUVEC grafts. (**A**) Body weight at the day of transplantation (day 0) and on days 15 and 30 post-transplantation in control and NaW-treated mice. (**B**) Fasting glycemia at days 15 and 30 post-transplantation in control and NaW-treated mice. (**C**,** D**) Quantitative analysis of vascularized area (**C**) and vessel density (**D**) in grafts at days 15 and 30 post-transplantation. For comparative purposes, data obtained from SC-islet grafts (without HUVEC) in NaW-treated mice at day 30 (Fig. 5B,C) are presented on the right of each graft. (**E**) Quantification of insulin-positive area in grafts from control and NaW-treated mice at day 30 post-transplantation. Values are expressed relative to control grafts, with values set to 100%. (**F**) Fasting human plasma insulin in control mice and mice treated with NaW at days 15 and 30 post-transplantation. (**G**) Glucose-stimulated insulin secretion at days 15 and 30 post-transplantation in control and NaW-treated mice. Values are expressed as the fold increase in plasma insulin 20 min after intraperitoneal glucose administration relative to initial fasting insulin levels for each animal (glucose stimulation index). Data were presented as mean ± SEM for the number of *n* indicated by dots. In (**A**, **B**, **F**, **G**): each dot represents an individual mouse. In (**C**, **D**): each dot represents one measured area, from a total of *n* = 5–7 grafts. In (**E**): each dot corresponds to the mean value from an individual graft. Statistical analysis was performed using two-way ANOVA (**A**–**D**, **F**, **G**) and unpaired Student’s *t*-test (**E** and in **C**, **D** for the comparison SC-islets and SC-islet/HUVEC at day 30).

Building upon our previous findings in iBeta-like/HUVEC spheroids, we next evaluated the impact of NaW treatment on grafts composed of SC-islets combined with exogenous endothelial cells. HUVECs were mixed with dissociated SC-islet cells at a 1:9 ratio, and the resulting aggregates were allowed to form spheroids in low attachment plates for three days prior to transplantation. NaW treatment did not affect body weight or fasting glycemia in transplanted mice (Fig. EV4A,B). At 15 days post transplantation, the NaW-treated group exhibited a higher graft vessel density, but the overall graft vascularization area was not significantly different compared to untreated controls (Fig. EV4C,D). By day 30, NaW-treated grafts had significantly increased vascularization and vessel density relative to the untreated group (Fig. EV4C,D). Interestingly, at day 30, the degree of vascularization area achieved in NaW-treated SC-islet/HUVEC grafts was similar to that observed in SC-islet grafts treated with NaW, although vessel density was 1.6-fold higher in the presence of HUVECs. Notably, these increases in vascular area and density were not accompanied by enhancements in beta cell mass or graft function, as indicated by unchanged insulin-positive area and circulating insulin concentrations between NaW-treated and control groups at day 30 post-transplantation (Fig. EV4E–G). These observations suggest that beyond a certain threshold, additional vascular density does not further improve SC-islet graft survival or function.

Collectively, these findings demonstrate that NaW exerts robust pro-angiogenic effects on SC-islet grafts independent of exogenous endothelial cell addition, and that these effects likely contribute to the improved transplantation outcomes observed in NaW-treated recipients.

## Discussion

Limited and delayed vascularization represents a major obstacle to the success of cell-based therapies for diabetes, as it impairs the timely delivery of oxygen and nutrients to transplanted insulin-producing cells. This deficiency compromises graft survival and function, ultimately undermining long-term therapeutic outcomes. In this study, we demonstrate that NaW, a small molecule characterized by favorable oral bioavailability and a well-established safety profile, significantly promotes early vascularization in grafts composed of either human fibroblast-derived iBeta-like cells or human stem cell-derived islets. Mechanistically, NaW exerts a dual pro-angiogenic effect: it enhances VEGFA production by the transplanted endocrine cells and simultaneously potentiates VEGF-dependent pro-vascular signaling pathways in endothelial cells. These complementary actions lead to improved graft integration and survival in vivo, highlighting NaW’s potential as a clinically relevant adjunct to enhance the efficacy of cell-based interventions for diabetes.

Several strategies have been proposed to accelerate vascularization, including VEGF overexpression, incorporation of accessory cell types, use of prevascularized scaffolds, and transplantation of adipose-derived microvessels. However, each approach presents significant regulatory, manufacturing, and biological challenges. VEGF overexpression demands precise control of dosage and timing to avoid excessive or disorganized angiogenesis, which can compromise graft morphology and function (Agudo et al, 2012; Cai et al, 2012). Cell- and scaffold-based strategies raise concerns regarding immunogenicity, scalability, and fibrotic responses. Adipose-derived microvessels (Aghazadeh et al, 2021), which preserve native vascular architecture and are minimally manipulated, offer promise but may be limited by reduced quality and angiogenic potential in patients with T1D (Lespagnol et al, 2020; Snaith et al, 2025), as well as by complex processing and sterility requirements that hinder widespread clinical application.

By contrast, NaW is an off-the-shelf, chemically defined small molecule that is orally bioavailable, has undergone prior clinical testing for use in metabolic diseases and fertility treatments, and has demonstrated an absence of toxicity (Hanzu et al, 2010; Arbat et al, 2025; Bertinat et al, 2015). This favorable profile supports rapid translation to applications where enhancing graft vascularization is required. NaW also offers practical advantages, including low cost, stability, and suitability for systemic administration, features that may facilitate clinical implementation. Moreover, its use can be readily combined with more sophisticated pro-angiogenic strategies, providing a straightforward means to further augment their efficacy.

Our data suggest that NaW promotes vascularization through a coordinated, VEGFA-centered mechanism. In transplanted endocrine cells, NaW increased VEGFA production, providing a paracrine signal for endothelial ingrowth. In endothelial cells, NaW potentiated VEGFA-induced proliferation, migration, tube formation, and ERK activation. Together, these findings indicate that NaW’s pro-angiogenic activity on endothelial cells is closely linked to VEGFA signaling, highlighting its potential as a controllable strategy to boost graft vascularization. Whether NaW influences later stages of vessel maturation, such as pericyte recruitment, basement membrane deposition, or vascular stabilization, remains to be determined.

Interestingly, our data indicate that NaW’s pro-angiogenic effects in SC-islet grafts are mediated primarily through host endothelial cells, without requiring the presence of endothelial cells in the donor islets. This finding simplifies graft preparation by eliminating the need to pre-incorporate endothelial components. Notably, treatment with NaW did not enhance insulin graft area or circulating insulin levels in mice transplanted with SC-islets containing HUVECs, despite a marked increase in vessel density compared to SC-islets without HUVECs. This apparent paradox may stem from overly robust vascularization, which can potentially reduce cell viability or disrupt the spatial arrangement of the graft (Brissova et al, 2014; Agudo et al, 2012; Cai et al, 2012). Additionally, adverse effects encountered during the assembly and maintenance of SC-islet + HUVEC spheroids in vitro may ultimately compromise both the survival and functional integration of the graft. In this study, we did not test whether adding endothelial cells at the time of transplantation, instead of during spheroid formation, might be beneficial. Further research is required to clarify these possibilities before concluding that donor endothelial cells do not provide added value beyond NaW treatment alone.

Importantly, the beneficial effects of NaW were reproduced across two independent cellular models, highlighting the robustness of this strategy. Such consistency suggests that the observed pro-angiogenic and survival benefits are not limited to a specific cellular platform and may therefore be broadly applicable to beta cell replacement therapies, including donor islet transplantation.

### Limitations of the study

This study primarily evaluated vascular outcomes, leveraging the ACE as a transplantation site due to its accessibility and suitability for high-resolution in vivo imaging. While the ACE provided clear advantages for assessing early graft vascularization, its translational relevance is limited. Although ongoing clinical trials are investigating the ACE as a potential site for donor islet transplantation in patients with type 1 diabetes (NCT04198350, NCT02846571, and NCT02916680), further investigation of NaW effects in other clinically relevant sites, such as the subcutaneous, omental or muscular sites, is warranted. These anatomical niches can accommodate larger graft volumes but present additional challenges in achieving adequate vascularization (Cayabyab et al, 2021; Bellin et al, 2019). Importantly, the restricted graft capacity within the murine ACE site in mice precluded detailed functional analysis under hyperglycaemic conditions, thereby preventing evaluation of long-term metabolic outcomes in the present study. Future work should therefore extend these observations to transplantation models with higher graft loads and longer follow-up, where direct assessment of glycaemic control can be achieved.

The present data support a pro-angiogenic effect of NaW in an immunodeficient mouse model at doses consistent with those established as safe in preclinical toxicological studies (NTP TR-599, 2021). Nevertheless, given its broad phosphatase-inhibitory activity, off-target effects on immune cell function and fibrotic remodeling cannot be dismissed and warrant careful evaluation in future work, particularly given that prior toxicological studies were not conducted in NSG mice. Future investigations should prioritize optimization of dosing regimens and timing, with an emphasis on transient use during the peri-transplantation period, when pro-angiogenic effects are most critically required. Localized delivery strategies, including nanoparticles, hydrogels, scaffolds, or direct co-formulation with grafts, represent a promising avenue to concentrate effects within the graft microenvironment while limiting systemic exposure.

An important limitation of this work is the lack of direct evidence linking the observed effects of NaW to the inhibition of specific protein tyrosine phosphatases. Although NaW is known to inhibit several PTPs, including PTP1B, the phosphatase(s) and signaling pathways responsible for the effects observed in iBeta-like and SC-islet grafts and endothelial cells remain undefined. Ongoing studies in our laboratory are focused on identifying the PTP(s) mediating these effects using in vitro approaches, which will subsequently be validated in in vivo transplantation models. Such studies will be necessary to establish the mechanistic basis of NaW action in this setting. Once these mechanisms are better characterized, more selective inhibitors targeting the relevant phosphatase(s) could be explored as an alternative translational strategy, potentially reducing off-target effects while preserving the beneficial pro-angiogenic activity observed in this study.

## Conclusions

In conclusion, our study identifies NaW as a promising pharmacological agent to enhance graft revascularization in islet cell transplantation. By acting on both transplanted cells and endothelial cells, NaW facilitates early functional vascularization, which is a key determinant of graft survival and long-term efficacy. Its favorable safety profile supports further investigation of NaW as a translatable strategy to improve the success of beta cell replacement therapies. Together with our previous findings showing that PTP1B-deficient donor islets exhibit improved graft survival and glycaemic control following transplantation, these results strengthen the rationale for targeting phosphatase-dependent signaling to optimize engraftment in islet and stem cell-based transplantation. More broadly, our work highlights the potential of combining soluble chemical modulators with existing and emerging biological and biotechnological approaches to advance engraftment in cell transplantation protocols.

## Methods

**Table:** Reagents and tools table

Reagent/resource	Reference or source	Identifier or catalog number
**Experimental models**		
NSG (*Mus musculus*)	Charles River Laboratories	NOD.*Cg-Prkdc*^*scid*^*Il2rg*^*tm1Wjl*^/SzJ
HUVECs	Life Technologies	Cat# C0155C
iBeta-cells	Fontcuberta-PiSunyer et al Commun Biol. 2023 Mar 24;6(1):256.	
SC-islets	Barsby et al STAR Protoc. 2022 Dec 16;3(4):101711	
Human islets	This study	
**Recombinant DNA**		
AdCMV-Ngn3-Pdx1-Mafa	Fontcuberta-PiSunyer et al Commun Biol. 2023 Mar 24;6(1):256.	
AdCMV-Nkx2-2	Fontcuberta-PiSunyer et al Commun Biol. 2023 Mar 24;6(1):256.	
AdCMV-Pax4	Fontcuberta-PiSunyer et al Commun Biol. 2023 Mar 24;6(1):256.	
**Antibodies**		
Guinea Pig Anti-Insulin	Dako	Cat# A0564
Mouse Anti-Glucagon	Sigma	Cat# G2654
Mouse Anti-HLA Class 1 ABC	Abcam	Cat# ab7328
Rat Anti-C-Peptide	Hybridoma Bank	Cat# GN-ID4
Rabbit Anti-Ki67	Invitrogen	Cat# MA5-14520
Sheep Anti-hCD34	Biotechne, R&D Systems	Cat# AF7227
Rat Anti-mCD34	eBioscience	Cat# 14-0341-85
Rabbit Anti-F4/80 D2S9R	Cell Signaling	Cat# 70076
Rabbit Anti-veCADHERIN	Abcam	Cat# ab33168
Rabbit Anti-VEGF	Millipore	Cat# ABS82
Rabbit Anti-Pdx1	Abcam	Cat# ab47267
Mouse Anti-Nkx6.1	Hybridoma Bank	Cat# F64A6B4
Mouse Anti-MafA	Novus Biologicals	Cat# NBP1-00121
Rabbit Anti-P-p44/42 MAPK (T202/Y204)	Cell Signaling	Cat# 9101
Rabbit Anti-p44/42 MAP Kinase	Cell Signaling	Cat# 9102
Mouse Anti-alpha Tubulin	Sigma	Cat# T6074
AlexaFluor 488 anti-rabbit	Jackson Immunoresearch Laboratories	Cat# 711-546-152
Cy2 anti-rat	Jackson Immunoresearch Laboratories	Cat# 712-225-153
Cy2 anti-sheep	Jackson Immunoresearch Laboratories	Cat# 713-225-147
AlexaFluor 555 anti-mouse	Molecular Probes	Cat# A31570
AlexaFluor 555 anti-rabbit	Molecular Probes	Cat# A31572
AlexaFluor 647 anti-rat	Jackson Immunoresearch Laboratories	Cat# 712-605-153
AlexaFluor 647 anti-rabbit	Jackson Immunoresearch Laboratories	Cat# 711-605-152
Anti-mouse IgG Peroxidase	GE Healthcare	Cat# NA931
Anti-rabbit IgG Peroxidase	GE Healthcare	Cat# NA934
**Oligonucleotides and other sequence-based reagents**		
*TBP*	This study	Appendix Table S3
*INS*	This study	Appendix Table S3
*NKX6-1*	This study	Appendix Table S3
*PCSK1*	This study	Appendix Table S3
*PCSK2*	This study	Appendix Table S3
*KCNJ11*	This study	Appendix Table S3
*ABCC8*	This study	Appendix Table S3
*TWIST2*	This study	Appendix Table S3
*CDH5*	This study	Appendix Table S3
*VEGF*	This study	Appendix Table S3
**Chemicals, enzymes and other reagents**		
Sodium tungstate dihydrate	Sigma	Cat# 223336-100G
Superfect Transfection Reagent	Qiagen	Cat# 301307
JetPei Transfection Reagent	Polyplus transfection	Cat# 101-40N
Cultrex© RGF BME	Biotechne, R&D Systems	Cat# 3433-005-01
MTT (3-(4,5-dimethylthiazol-2-yl)-2,5-diphenyltetrazolium bromide)	Thermo Fisher Scientific	Cat# M6494
Rhodamine B isothiocyanate-Dextran	Sigma	Cat# R9379-100MG
Hoechst 33342	Invitrogen	Cat# H3570
Propidium iodide	Invitrogen	Cat# P3566
Vybrant™ CFDA SE Cell Tracer Kit	Molecular Probes	Cat# V12883
DeadEnd Fluorometric Tunel System	Promega	Cat# G3250
RPMI-1640	Sigma	Cat# R8758
DMEM 4,5g/L	Sigma	Cat# D6429
EGMTM-2 Endothelial Cell Growth Medium-2 BulletKit	Lonza	Cat# H3CC-3162
GlutaMAX	Thermo Fisher Scientific	Cat# 35050061
Penicillin-Streptomycin 100X solution	Hyclone	Cat# SV30010
L-glutamine	Hyclone	Cat# SH30034.01
Fetal Bovine Serum	Biosera	Cat# FB-1001
**Software**		
GraphPad Prism v. 8.0.1	Dotmatics https://www.graphpad.com/features	
ImageJ/Fiji	https://imagej.net/software/fiji/	
ImageJ Angiogenesis Analyzer macro	Carpentier et al Sci Rep. 2020 Jul 14;10(1):11568	
GIMP 3.2.2 (GNU Image Manipulation Program)	https://gimp.org	
Inkscape v1.4.3	https://inkscape.org	
**Other**		
Leica TCS SP5	Leica Microsystems	
Leica TCS SPE	Leica Microsystems	
OLYMPUS IX51	Olympus	
QuantStudio 5 qPCR	Applied Biosystems	
Infinite^®^ Mnano+ 200 PRO	Tecan	
Countess™ Automated Cell Counter	Invitrogen	
ImageQuant LAS4000	GE Healthcare Bio-Sciences	
ESCO CelCulture CO_2_ Incubator	Esco Lifesciences	

### Study design

The aim of this study was to determine whether sodium tungstate, a PTP1B inhibitor, promotes vascularization of beta-like cell grafts. Two cellular models were used: human fibroblast-derived beta-like cells and embryonic stem cell-derived islets, with or without co-transplantation of human umbilical vein endothelial cells (HUVECs). Grafts were transplanted into the anterior chamber of the eye of immunodeficient NSG mice and analysed at multiple time points up to day 30. NaW was administered in drinking water post-transplantation. Functional vascularization was assessed by RITC-dextran injection and visualized using two-photon confocal microscopy. Explanted grafts were fixed and processed for immunofluorescence to quantify cell death (TUNEL), proliferation (Ki67), insulin-positive and glucagon areas, and reprogramming rates. VEGFA expression within grafts was examined to explore angiogenic mechanisms. Graft functionality was determined in SC-islet transplants by measuring plasma human insulin levels under basal conditions and following glucose challenge. In vitro studies assessed the direct effects of NaW on beta-like cells by measuring VEGFA expression and secretion, and on endothelial cells using HUVECs as cell model to evaluate viability, proliferation, migration, wound healing, and signaling pathways via immunoblotting.

No statistical methods were used to predetermine sample size. Sample sizes were chosen based on previous experience with the experimental system and are consistent with those commonly used in the field. Mice were randomly allocated to experimental groups and identified by unique codes. Animals that died perioperatively or exhibited severe distress (e.g., >20% weight loss or labored breathing) were euthanized by CO₂ inhalation and excluded. Only mice surviving the predefined timeline were included in analyses. All procedures were approved by the Animal Ethics Committee of the University of Barcelona (113/22 and 344/23) and conducted in compliance with FELASA guidelines, Spanish Royal Decree 53/2013, and European Directive 2010/63/EU. Animal experiments followed the ARRIVE 2.0 guidelines. For in vitro experiments, treatments were assigned to wells at random. Each in vitro experiment included a minimum of two biological replicates, with exact numbers provided in figure legends. Samples were excluded only if they did not meet predefined quality-control requirements. Investigators were not blinded during experiments, outcome assessment, or data analysis.

### Mice

NOD scid gamma (NSG™) mice were maintained in the barrier animal facility at the University of Barcelona under a 12-h light/dark cycle with unrestricted access to food and water. Male and female mice aged 8–20 weeks served as recipients for transplantation experiments.

### iBeta-like/HUVEC spheroids

Human foreskin fibroblasts were reprogrammed into insulin-producing beta-like cells (iBeta-like) using five transcription factors: Neurog3, Pdx1, Mafa, Pax4, and Nkx2.2, as previously described (Fontcuberta-PiSunyer et al, 2023). Human umbilical vein endothelial cells (HUVEC; Life Technologies, Thermo Scientific, USA) were routinely grown in Microvascular Endothelial Cell Growth Medium-2 (EGM-2) (Lonza, Switzerland) and antibiotics. Mycoplasma testing was routinely carried out in our tissue culture facility.

To generate mixed iBeta-like/HUVEC spheroids, HUVECs were incorporated at a 1:9 ratio (HUVEC:iBeta-like) at the time of spheroid formation during the reprogramming protocol. In brief, spheroids containing a total of 1200–1600 cells per spheroid were prepared one day after Ad-Nkx2.2 infection using AggreWell-800 plates (STEMCELL Technologies, Canada). Cell aggregation and subsequent 6-day culture were performed in a hybrid medium composed of 75% RPMI-1640 supplemented with 6% (v/v) fetal bovine serum (FBS, Biosera, France), 1% (v/v) L-glutamine (Hyclone, Cytiva, USA), 25% EGM-2 and antibiotics. This medium composition was initially tested to confirm cell viability and lack of interference with the reprogramming process. The 1:9 proportion was chosen to roughly approximate the estimated <5% of endothelial cell content and average 10% vascular volume of native islets (Fasolino et al, 2022; Félix-Martínez et al, 2025; Grapensparr et al, 2018). Higher HUVEC proportions reported in the literature (Wassmer et al, 2021; Burganova et al, 2021) were also evaluated; however, 1:4 and 1:1 HUVEC:iBeta-like ratios produced spheroids with irregular morphology that failed to maintain structural integrity and progressively dissociated over the 6-day culture period.

For hypoxia experiments, iBeta-like cells were maintained in six-well plates for 3 days after Nkx2-2 introduction and then treated with or without NaW (0.2 or 1 mM) in RPMI-1640 supplemented with 6% (v/v) FBS, 1% (v/v) L-glutamine and antibiotics. Cells were incubated under hypoxic conditions (3% O₂), achieved by regulated nitrogen supplementation, for 48 h. Control cells were maintained under normoxic conditions (21% O₂) in a standard incubator. Following incubation, cell pellets were collected for gene expression analysis, and culture media were harvested for quantification of human VEGFA levels using an ELISA kit (RayBiotech, USA).

### Human islets

Human islets were prepared by collagenase digestion followed by density gradient purification at the Laboratory of Cell Therapy for Diabetes (Hospital Saint-Eloi, Montpellier, France). After reception, human islets were maintained in culture for 1–3 days in RPMI-1640 with 5.5 mM glucose, 10% fetal bovine serum (FBS) and antibiotics, before performing the transplantation experiments. Islets from two donors (Appendix Table S1) were used for Fig. 1C–E. Experiments involving human islets were performed in agreement with the local ethics committee (CHU, Montpellier) and the institutional ethical committee of the French Agence de la Biomédecine (DC Nos. 2014-2473 and 2016-2716). Informed consent was obtained from donors. Experiments conformed to the principles set out in the WMA Declaration of Helsinki and the Department of Health and Human Services Belmont Report.

### SC-islets

The use of human embryonic stem-cell lines does not require a specific ethical approval according to Finnish legislation. The H1 embryonic stem-cell line (WiCell, WA01) was differentiated to SC-islets with a seven-stage protocol (Balboa et al, 2022) detailed in Barsby et al (Barsby et al, 2022). Briefly, the human embryonic stem cells were induced to differentiate through definitive endoderm, primitive gut tube and posterior foregut-like states in a monolayer to pancreatic progenitors using media changes with defined small-molecule and growth-factor compositions. Two days after pancreatic progenitor stage initiation, the cells were dissociated and reaggregated using AggreWell 400 plates (STEMCELL Technologies). Then, aggregates were transferred to suspension culture (6-well plates, in rotation), and further differentiated through an endocrine progenitor state to insulin- and glucagon-positive endocrine cells. The final SC-islets were matured for 3–4 weeks in the final stage of the differentiation prior to being transplanted. Quality-control checkpoints were performed using flow cytometry at definitive endoderm stage (determining CXCR4+ %), pancreatic progenitor stage (PDX1 + NKX6.1+ %) and functional SC-islet stage (Stage 7 week 3, PDX1 + NKX6.1 + %, NKX6.1 + CPEP + %, and INS + GCG + %), performed as previously described(Balboa et al, 2022; Barsby et al, 2022). SC-islet functionality was assessed using dynamic glucose-stimulated insulin secretion in response to low glucose (G2.8 mM), high glucose (G16.8 mM), stimulated with GLP1 analog Exendin 4 (G16.8 mM + Ex4) and a final membrane depolarization stimulus (G2.8 mM+ KCl), as detailed previously (Balboa et al, 2022; Barsby et al, 2022). SC-islets were shipped from the University of Helsinki to IDIBAPS and maintained for 24 h in S7 medium under static conditions prior to transplantation. For SC-islet/HUVEC graft preparation, SC-islets were dissociated 48 h after arrival, mixed with HUVECs at a 9:1 ratio, reaggregated in AggreWell plates (1500 cells/well), and cultured for an additional 72 h prior to transplantation.

### Transplantation into the anterior chamber of the eye

iBeta-like spheroids, SC-islets and human islets were labeled with 10 μM CFDA SE (Molecular Probes, Thermo Fisher Scientific, USA), for 15 min at 37 °C before the transplant. NSG^TM^ mice were transplanted with 250–300 iBeta-like cell spheroids, SC-islets or human islets in the anterior chamber of the eye (ACE) as previously described (Figueiredo et al, 2019). Briefly, receptor mice were anesthetized with a mix of ketamine + xylazine (80 mg/Kg + 5 mg/Kg) intraperitoneally and received a subcutaneous injection of the analgesic buprenorphine (0.1 mg/Kg). Spheroids and islets were introduced in the ACE under a stereomicroscope, using a blunt polythene cannula (Fine Bore Polythene Tubing, Smiths Medical International, UK) and a Hamilton syringe. Viscofresh (10 mg/ml sodium carmellose) was used to relieve eye discomfort when the mice recovered from the anesthetics.

### Sodium tungstate treatment

After transplantation, mice were randomly assigned to receive NaW or no treatment. NaW dihydrate (Na_2_WO_4_·2H_2_O, Sigma, USA) was provided ad libitum in the drinking water at 1 g/L, with an average consumption estimated at ~125 mg/kg body weight/day. Toxicology data show that 1 g/L is well tolerated long-term, with only mild renal changes after prolonged exposure, and 28-day studies report no clinical or organ-level toxicity at this dose (Frawley et al, 2016; Toxicology and carcinogenesis studies of sodium tungstate dihydrate in Sprague Dawley rats and B6C3F1/N mice (drinking water studies), 2021).

For in vitro experiments, NaW concentrations ranged between 0.2 and 1 mM, selected to achieve functionally relevant PTP1B inhibition consistent with the reported IC₅₀ for tungstate (~210 µM) (Bellomo et al, 2016) and with concentrations previously shown to exert biological activity across multiple cellular models (Domínguez et al, 2003; Osterburg et al, 2010; Montero-Martin et al, 2024).

### In vivo vascularization and viability imaging

Functional graft revascularization was assessed as described (Figueiredo et al, 2019) at the indicated days after transplantation by a two-photon microscopy equipped with an incubation system with temperature control (Leica TCS SP5, Leica Microsystems, Germany). At the time of each experiment, anesthetized mice received an intravenous injection of 80 mg/Kg of rhodamine B isothiocyanate-dextran (RITC-dextran, functional vascular tracer) (Sigma, USA) and 2.5 μg/Kg of Hoechst (nuclear tracer) (Invitrogen, Thermo Fisher Scientific, USA). First, optical fluorescence was used to localize CFDA-positive grafts. Then, stacks of the grafts were acquired every 0.29 µm using a 25x water immersion objective (HCX IR APO L 25x, Numerical Aperture 0.95, Leica Microsystems), resonant scanner at 8000 lines and blue diode (405 nm), Argon (488 nm) and diode pumped solid state (561 nm). The confocal pinhole was set to its maximum opening. By the end of the visualization, animals were euthanized to perform eye enucleation for posterior immunofluorescence analysis. Collected images were analysed using ImageJ/Fiji software (v1.54p; National Institutes of Health, USA). Vascularization was quantified using two parameters. Vascularization area (%) was calculated as the percentage of the graft area occupied by vessels, defined as the total area positive for RITC-dextran relative to the total CFDA+ graft area, which reflects the overall extent of vascular coverage within the graft (integrating vessel number and calibre). Vessel density was determined as the number of discrete blood vessels per unit graft area and reflects the abundance of individual vascular structures within the graft, consistent with increased formation of new capillary branches rather than vessel dilation or maturation.

### In toto staining

After collection from the plate, spheroids were transferred into a 1.5 mL Eppendorf tube, washed, and fixed in 4% paraformaldehyde for 15 min at 4 °C. Immunofluorescence staining of whole spheroids was performed in suspension under a stereomicroscope. Samples were permeabilized with 0.8% Triton X-100 and blocked using a solution containing 10% (v/v) FBS and 5% (v/v) normal donkey serum (Jackson Immunoresearch Laboratories, USA). For subsequent washes, 0.3% (v/v) Triton X-100 was used, and antibody solutions (Appendix Table S2) were prepared with 5–10% (v/v) FBS. Following staining with the indicated antibodies, samples were mounted on a chamber slide to preserve their three-dimensional structure and imaged using a Leica TCS SPE confocal microscope. Z-stack images were acquired, and sections were analysed at 5 μm intervals using ImageJ/Fiji software.

### Gene expression analysis

For gene expression assays, total RNA was isolated using NucleoSpin^®^RNA (Macherey-Nagel, Germany) following the manufacturer’s instructions. First-strand cDNA was prepared using Superscript III Reverse Transcriptase (Invitrogen) or High-Capacity cDNA Reverse Transcription Kit (Applied Biosystems, Thermo Fisher Scientific, USA). Real-time PCR was performed on an ABI Prism 7900 or Applied Biosystems QuantStudio™ 5 Real-Time PCR system, using Gotaq master mix (Promega, USA). Expression relative to the housekeeping gene *TBP* was calculated using the delta(d)Ct method and expressed as 2^(-dCT). Primer sequences are provided in Appendix Table S3.

### Assessment of insulin secretion

To evaluate glucose-stimulated insulin secretion in vitro, iBeta-like or iBeta-like/HUVEC spheroids were first washed with phosphate-buffered saline (PBS) and pre-incubated in Krebs-Ringer Buffer containing 2 mM glucose for 45 min at 37 °C. Following this equilibration step, spheroids were sequentially incubated in Krebs Buffer with low glucose (2 mM) and subsequently with high glucose (20 mM) for 60 min at 37 °C. SC-islets were cultured in S7 medium without ITS-X and agitation for 3–4 days and then treated with NaW (0.2 mM) for 24 h. GSIS assays were performed as described above, using incubation periods of 1 h, 45 min, and 45 min for pre-incubation, basal glucose, and stimulatory glucose conditions, respectively. Supernatants were collected after each incubation period for insulin quantification. At the end of the secretion assay, SC-islets were harvested for insulin content determination by acid-ethanol extraction.

For in vivo assessment of glucose-induced insulin secretion, mice were fasted for 6 h prior to receiving an intraperitoneal glucose injection (3 g/Kg of body weight). Blood samples were collected at time 0- and 20-min post-injection for human insulin measurement.

Human insulin concentration in both in vitro supernatants and mouse plasma was determined using a Human Insulin ELISA kit (Crystal Chem, USA), following the manufacturer’s instructions.

### Immunofluorescence

The eyes containing grafts were fixed overnight in 2% paraformaldehyde and subsequently embedded in paraffin. Each graft was fully sectioned at 3 μm, and sections were distributed across two sets of ten slides, with five columns of three sections per slide. Consecutive columns were spaced 90-μm apart, such that each slide spanned 450 μm of tissue depth. For immunofluorescence analysis, a minimum of ten sections per graft were selected from two non-adjacent slides separated by at least five intervening slides, to ensure representative coverage of the graft. Tissue sections were rehydrated and subject to heat-mediated antigen retrieval in citrate buffer, permeabilized using 1% Triton X-100 and blocked in 5% normal donkey serum. Then, grafts were incubated at 4 °C overnight with primary antibodies and for 2 h at room temperature with fluorescent-labeled secondary antibodies (Appendix Table S2). For the apoptosis analysis, DeadEnd Fluorometric TUNEL System (Promega) was performed according to the manufacturer's instructions. In all cases, images were acquired in a Leica TCS SPE confocal microscope, using a 40x oil immersion objective. Images of positive cells and areas were quantified by using ImageJ/Fiji software.

### Cell viability

To assess iBeta-like cell viability, cell spheroids were labeled with 10 µM CFDA SE, 2.5 µg/mL propidium iodide (PI) (Invitrogen) for 15 min at 37 °C. Spheroids were then visualized using Leica TCS SPE confocal, and images were analysed using ImageJ/Fiji software. Cell viability was calculated as the percentage of CFDA⁺PI⁻ cells relative to the total number of cells within each spheroid.

HUVEC viability was assayed by MTT assay. In brief, HUVECs were seeded in 96-well plates (10,000 cells/well). After 24 h, the medium was replaced with fresh EGM-2 containing VEGFA (2 ng/mL), NaW (0.2 mM), both, or neither (control). After 48 h of treatment, cells were incubated with 0.75 mg/mL MTT (3-(4,5-dimethylthiazol-2-yl)-2,5-diphenyltetrazolium bromide) for 3 h at 37 °C. Formazan crystals were dissolved in isopropanol with 0.04 N HCl, and absorbance was measured at 575 and 650 nm using a TECAN Infinite® 200 PRO reader (Tecan, Switzerland). Corrected OD (OD575–OD650) was normalized to the control (100%).

### Evaluation of angiogenic properties in HUVECs

#### Proliferation assay

HUVECs were seeded at 10,000 cells/well in 96-well plates. After 24 h, the medium was replaced with EGM-2 supplemented with VEGFA (2 ng/mL), NaW (0.2 mM), both, or neither (control). Cell proliferation was evaluated at 36 and 72 h post-treatment. At each time point, cells were trypsinized, neutralized with DMEM containing 10% FBS, and counted using the Countess™ Automated Cell Counter (Invitrogen).

#### Migration assay

Cell migration was assessed following established wound healing protocols (Luna et al, 2015). HUVECs were seeded at 250,000 cells/well in 12-well plates and cultured to 90–100% confluence. A scratch was made using a sterile pipette tip, and cells were treated with NaW (0.2 mM), VEGFA (2 ng/mL), both, or control. Migration into the wound area was monitored at 0, 2, 4, 6, 8, and 24 h using an inverted optical microscope (OLYMPUS IX51, Germany). Images were analysed with ImageJ/Fiji software to quantify the percentage of wound closure relative to the initial scratch area.

#### Tube formation assay

Endothelial tube formation was assessed following established protocol (DeCicco-Skinner et al, 2014; Xie et al, 2016). A total of 150 µl/well of reduced Growth Factor Basement Membrane Extract (Cultrex© RGF BME, R&D Systems, USA) was added to 48-well plates and incubated at 37 °C with 5% CO₂ for 30 min to allow gelation. HUVECs (40,000 cells/well) were then seeded in conditioned medium containing NaW (0.2 mM), VEGFA (2 ng/mL), both, or control. After 3–5 h of incubation, tube-like structures were imaged using an inverted optical microscope (OLYMPUS IX51). Five representative images per well (center and cardinal axes) were analysed using the ImageJ Angiogenesis Analyzer macro (Carpentier et al, 2020) to quantify tube formation parameters.

### Immunoblot analysis

About 250,000 HUVEC cells/well were seeded in six-well plates and cultured for 14–16 h in low serum DMEM (0.2% FBS). Cells were treated for 10 min with NaW, VEGFA (25 ng/mL), or both. Cells were lysed in triple detergent buffer (50 mM Tris-HCl, 150 mM NaCl, 0.1% SDS, 1% NP40, 0.5% sodium deoxycholate) with phosphatase and protease inhibitors (Roche, Switzerland). Lysates were frozen/thawed twice and sonicated (5 × 30 s bursts at 20 kHz). Debris was pelleted, and protein quantified using the Lowry assay (Bio-Rad, USA). About 15 μg of protein per replicate was separated on 4–12% gradient gels (Bio-Rad) and transferred to PVDF membranes (Perkin Elmer, USA). Membranes were incubated overnight at 4 °C with antibodies against ERK1/2, phospho-ERK1/2 and α-TUBULIN as a loading control. Detection was performed using HRP-conjugated secondary antibodies and ECL reagent (Pierce, Thermo Fisher Scientific, USA). Bands were visualized with LAS4000 (GE Healthcare Bio-Sciences, Sweden) and quantified using ImageJ/Fiji. Antibody information is provided in Appendix Table S2.

### Statistics

Data were analysed using GraphPad Prism version 8.00 for Windows (www.graphpad.com) and presented as the mean ± standard error of the mean (SEM). Unpaired Student’s t-tests were used for comparison of two groups. One-way ANOVA or two-way ANOVA were used according to the appropriate number of comparisons, followed by recommended post hoc tests (Tukey’s or Dunnett’s), applied to determine significant differences among multiple comparisons. Formal normality testing was not routinely performed because of the sample sizes used. No obvious violations of test assumptions were observed. Differences were considered significant at *P* < 0.05. *P* values greater than 0.05 and lower than 0.1 were considered trends.

## Supplementary information

Appendix

Peer Review File

Source data Fig. 1

Source data Fig. 3

Source data Fig. 4

Source data Fig. 2

Source data Fig. 5

Expanded View Figures

## Data Availability

This study includes no data deposited in external repositories. The source data of this paper are collected in the following database record: biostudies:S-SCDT-10_1038-S44321-026-00523-4.
