# Supplementary material for: Sodium tungstate promotes vascularization to support beta cell replacement in diabetes
Source: EMBO Mol Med. 2026 Sep 23;18(11):4334–56. doi: 10.1038/s44321-026-00523-4 (PMC13748639; doi:10.1038/s44321-026-00523-4)
Supplement: Supplementary file 1 [file 44321_2026_523_MOESM1_ESM.pdf]

Appendix

Sodium tungstate promotes vascularization to support beta cell replacement in diabetes

Ainhoa García-Alamán[1,2,3], Marta Fontcuberta-PiSunyer[1], Jonna M Saarimäki-Vire[4], Nea Asumaa[4], Marta Perea-Atienzar[1,2,3], Rebeca Fernandez-Ruiz[1,2], Hugo Alves-Figueiredo[5], Christophe Broca[6], Joan-Marc Servitja[1,2], Ramon Gomis[1], Diego Balboa[4], Josep Vidal[1,2,7], Rosa Gasa[1,2]

[1] Institut d'Investigacions Biomèdiques August Pi i Sunyer (IDIBAPS), Barcelona, Spain

[2] CIBER de Diabetes y Enfermedades Metabólicas Asociadas, Instituto de Salud Carlos III, Madrid, Spain

[3] Faculty of Medicine and Health Sciences, Universitat de Barcelona, Barcelona, Spain

[4] Stem Cells and Metabolism Research Program, Faculty of Medicine, University of Helsinki, Helsinki, Finland

[5] Tecnológico de Monterrey, Escuela de Medicina y Ciencias de la Salud, Monterrey N.L., México

[6] CHU Montpellier, Laboratory of Cell Therapy for Diabetes (LTCD), Hospital St-Eloi, Montpellier, France

[7] Endocrinology and Nutrition Department, Hospital Clinic of Barcelona, Barcelona, Spain

Appendix contents

Appendix Figure S1……………………………………………………………………….3
Proliferation of iBeta-like cells after transplantation
Appendix Figure S2……………………………………………………………………….4
Body weight and glycemia in mice transplanted with iBeta-like/HUVEC spheroids
Appendix Figure S3……………………………………………………………………….5
Histological characterization of macrophage infiltration in iBeta-like/HUVEC grafts
Appendix Figure S4……………………………………………………………………….6
Characterization of SC-islet differentiation and function
Appendix Figure S5 ……………………………………………………………………….7
Body weight and glycemia in mice transplanted with SC-islets
Appendix Figure S6……………………………………………………………………….8
Histological characterization of macrophage infiltration in SC-islet grafts
Appendix Figure S7……………………………………………………………………….9
Histological characterization of beta cells in SC-islet grafts
Appendix Figure S8……………………………………………………………………...10
Effects of NaW on SC-islets *in vitro*
Appendix Table S1………………………………………………………………………11
Human donor islet information
Appendix Table S2……………………………………………………………………..…12
Antibody information
Appendix Table S3……………………………………………………………………..…13
Primer sequences information

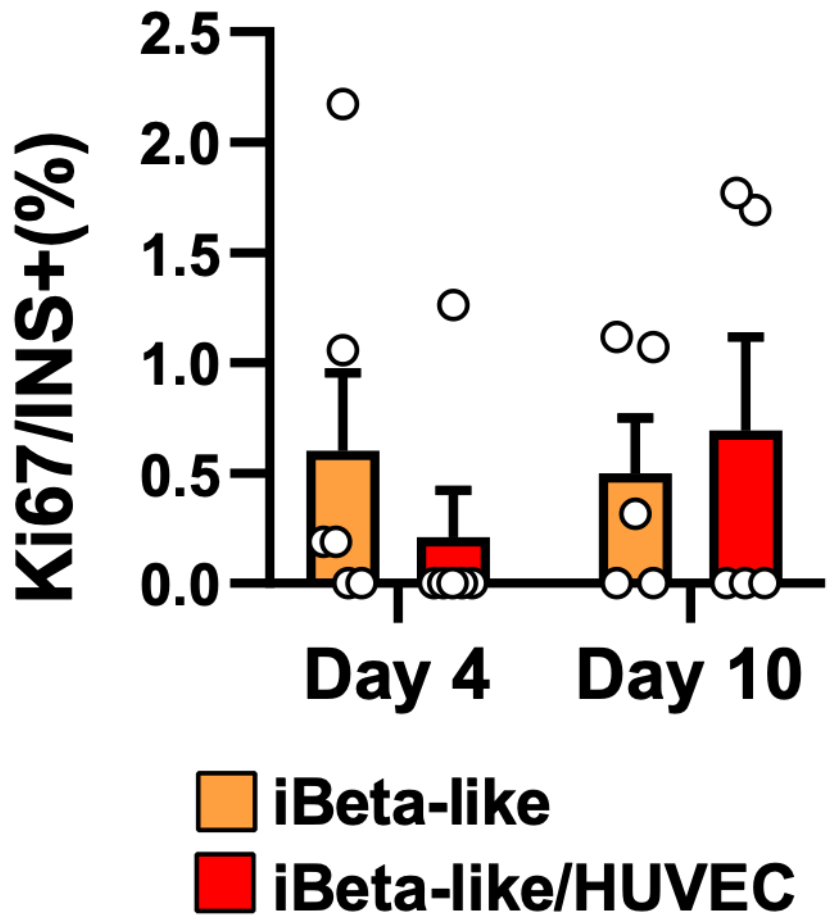

Appendix Figure S1. Proliferation of iBeta-like cells after transplantation.

Cell proliferation was assessed as the percentage of INS^+ cells co-expressing Ki67, relative to the total number of INS^+ cells, in grafts of the indicated types. Data are presented as mean ± SEM for the number of n indicated by dots, with each dot representing an individual graft. Statistical analysis was performed using two-way ANOVA; no significant differences were observed.

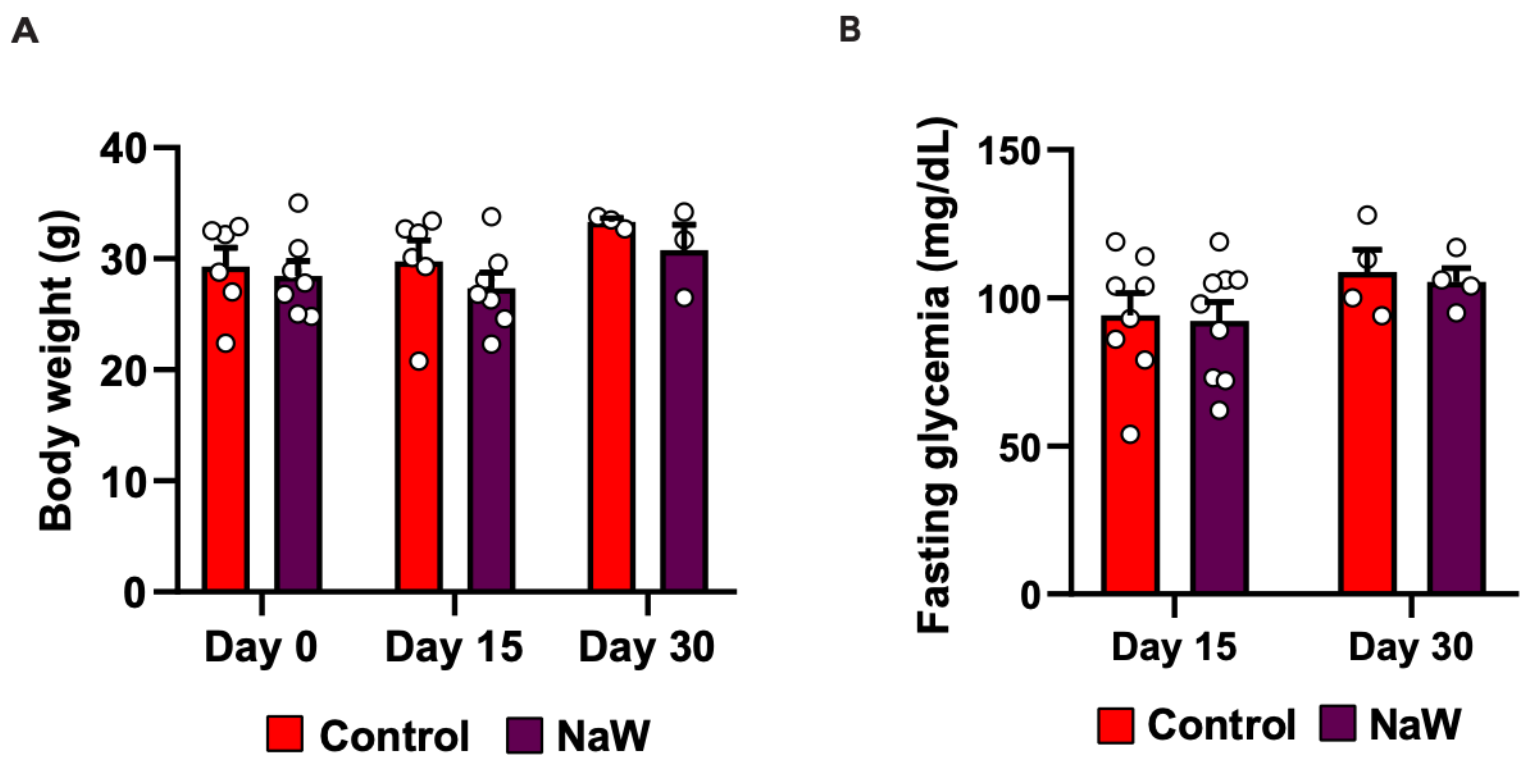

Appendix Figure S2. Effects of NaW treatment on body weight and glycemia in mice transplanted with iBeta-like/HUVEC spheroids.

Body weight **(A)** and fasting glycemia **(B)** were measured at the day of transplantation (day 0) and on days 15 and 30 post-transplantation in control and NaW-treated mice. Data are expressed as mean ± SEM, with each dot representing an individual animal (n as indicated). Statistical analysis was performed using two-way ANOVA, which revealed no significant differences.

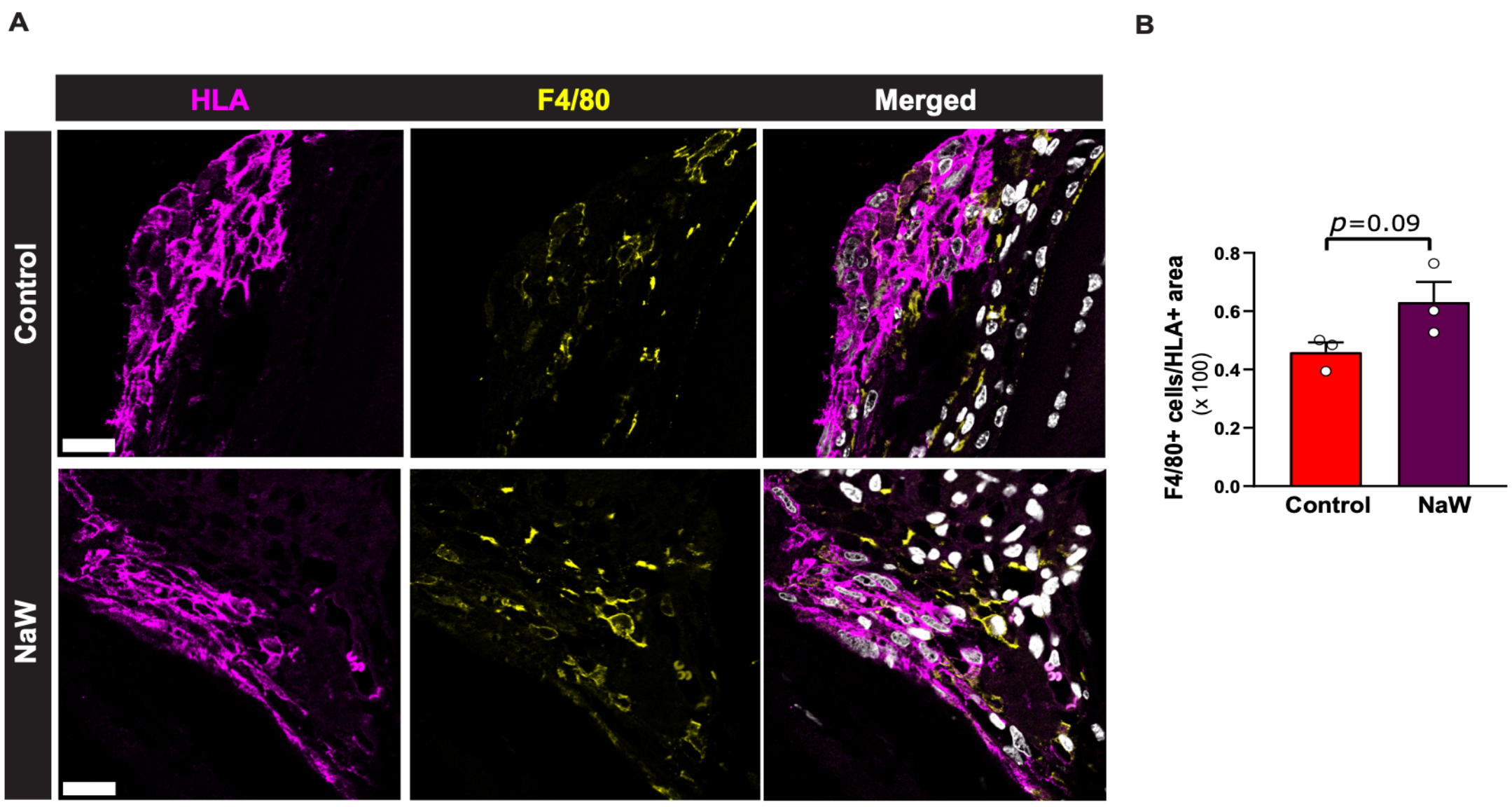

Appendix Figure S3. Histological characterization of macrophage infiltration in iBeta-like/HUVEC grafts at day 10 post-transplantation.

(A) Representative immunofluorescence images of iBeta-like/HUVEC grafts at day 10 post-transplantation in control and NaW-treated mice. Grafts were stained for HLA (magenta; human cells), F4/80 (yellow; macrophages) and Hoechst (white; nuclei). Scale bar: 25 µm. **(B)** Quantification of $F4/80^+$ cells located within or in direct contact with the HLA^+ graft area at day 10 post-transplantation in control and NaW-treated mice. Data are presented as mean ± SEM; each dot represents the mean value from an individual graft. Statistical analysis was performed using an unpaired two-tailed Student's t-test.

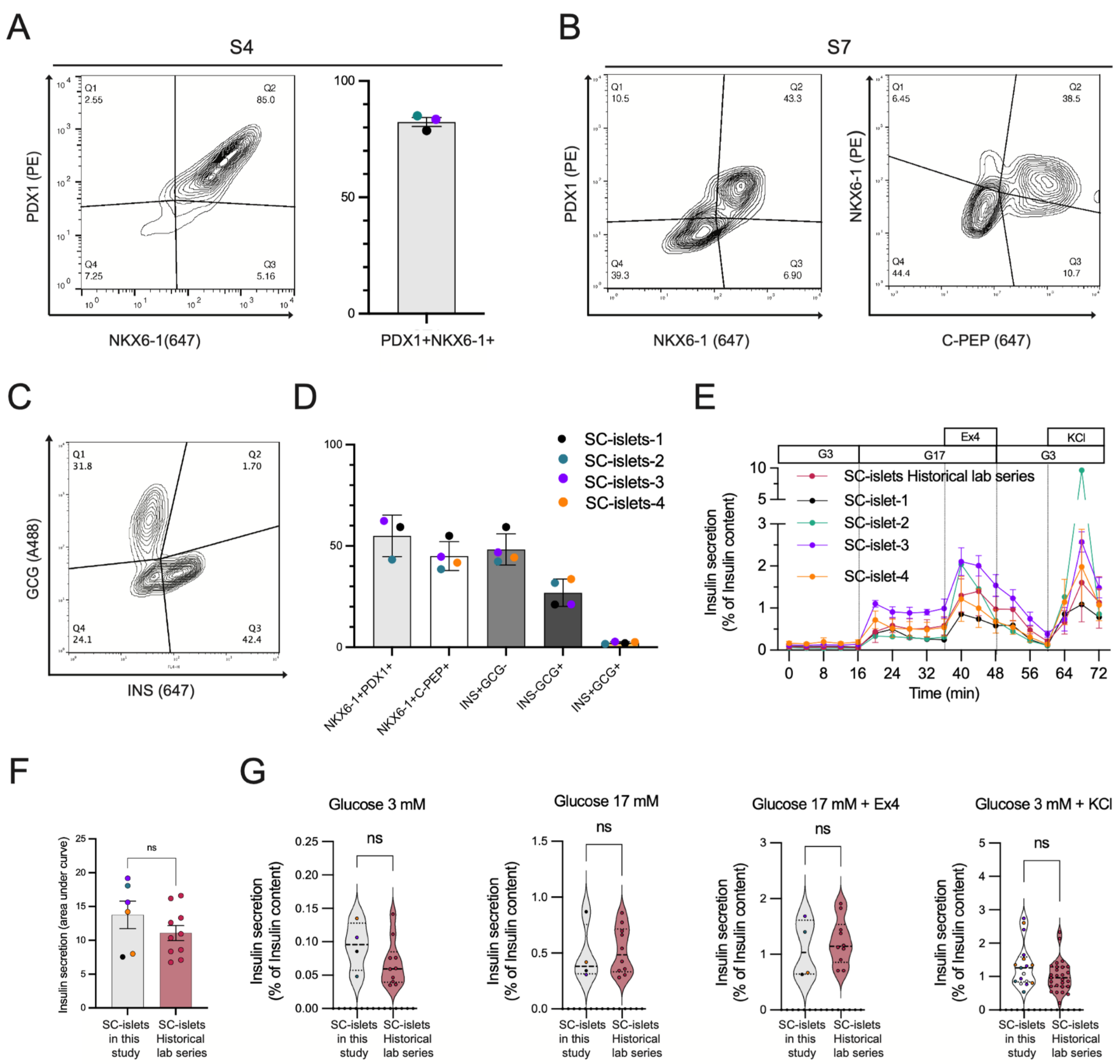

Appendix Figure S4. Characterization of SC-islet differentiation and function.

(A) Flow cytometry analysis with PDX1 and NKX6-1 antibodies in pancreatic progenitor stage (S4).

(B,C) Flow cytometry analysis of SC-islets at S7 week 3 with PDX1 + NKX6-1, NKX6-1+ C-PEP antibodies **(B)**, and INS + GCG antibodies **(C)**.

(D) Quantification of SC-islet populations using flow cytometry of the SC-islet differentiations used in this study.

(E) SC-islet dynamic glucose stimulated insulin secretion, in low 3mM glucose (G3), high 17 mM glucose (G17), high glucose + 50ng/ml Exendin4 (Ex4) and low glucose + 30mM KCl, presented as % of protein content.

(F) Area under the curve quantifications of data in (E).

(G) Insulin secretion as percentage of total insulin for the different stimuli presented in (E). Data are presented as mean ± SEM. Colored dots indicate individual SC-islet batches. ns: not statistically significant. Mann-Whitney U-test.

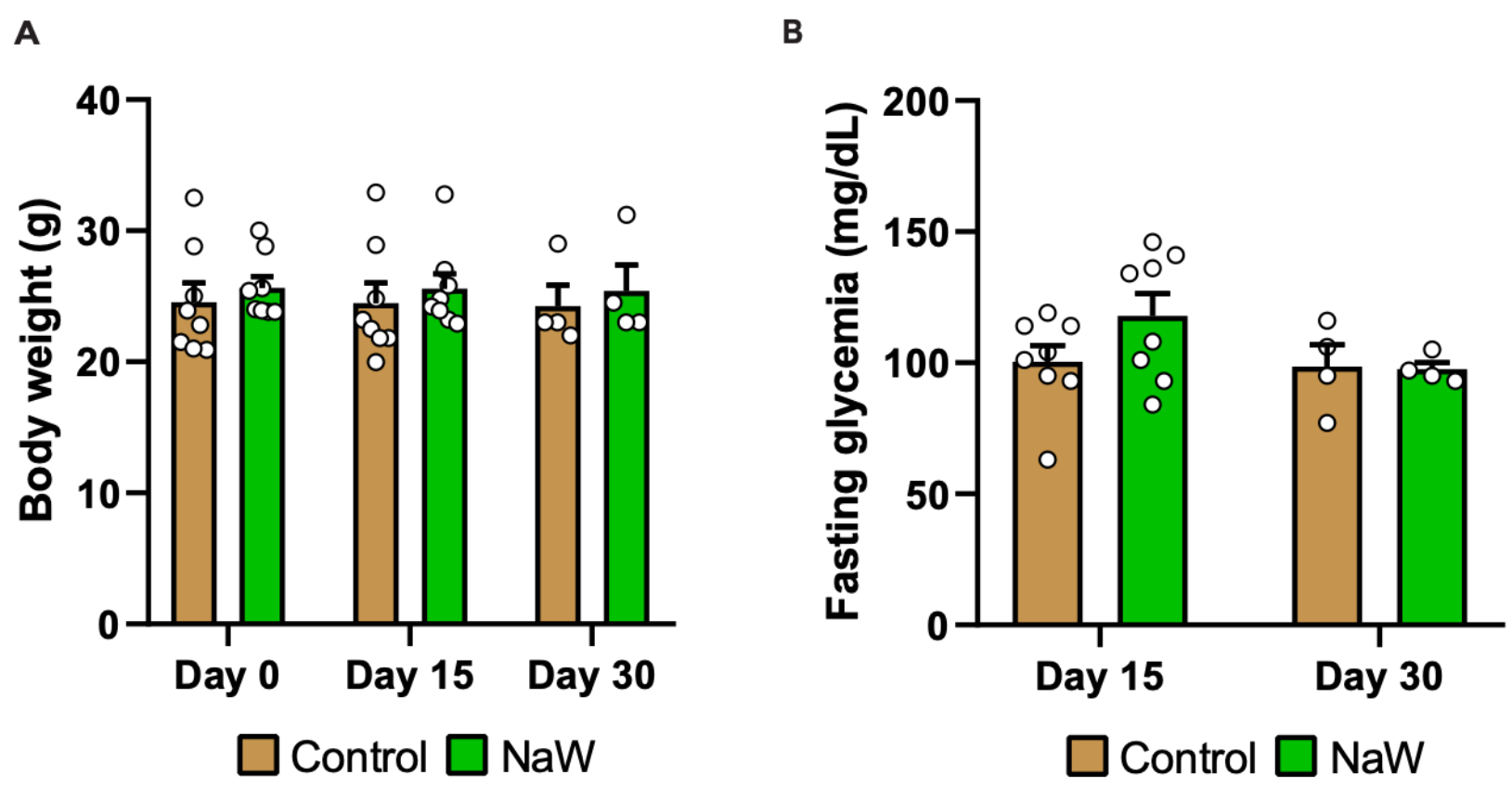

Appendix Figure S5. Effects of NaW treatment on body weight and glycemia in mice transplanted with SC-islets.

Body weight **(A)** and fasting glycemia **(B)** were measured at the day of transplantation (day 0) and on days 15 and 30 post-transplantation in control and NaW-treated mice. Data are expressed as mean ± SEM, with each dot representing an individual animal (n as indicated). Statistical analysis was performed using two-way ANOVA, which revealed no significant differences.

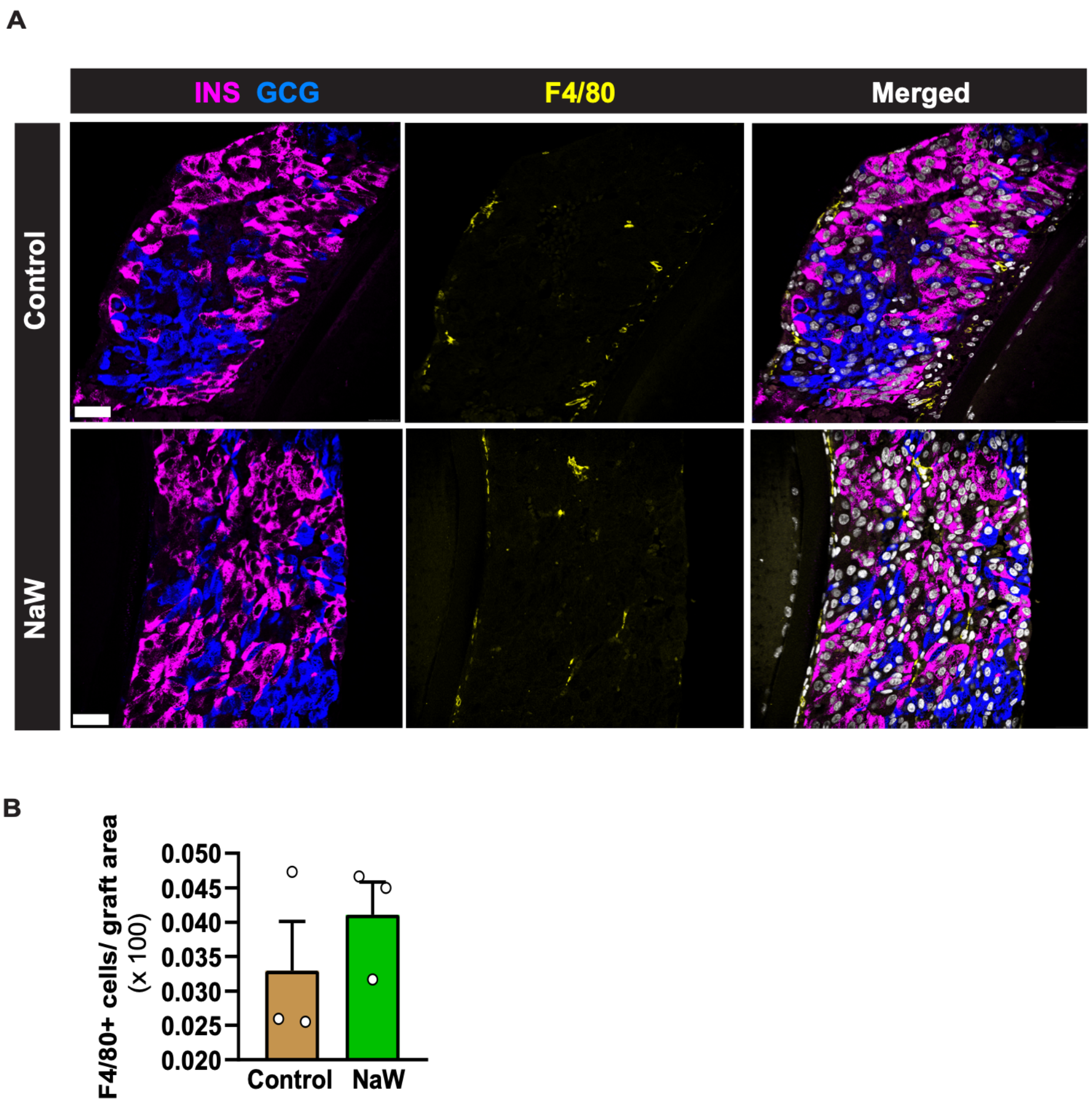

Appendix Figure S6. Histological characterization of macrophage infiltration in SC-islet grafts at day 30 post-transplantation.

(A) Representative immunofluorescence images of SC-islet grafts at day 30 post-transplantation in control and NaW-treated mice. Grafts were stained for insulin (INS, magenta), glucagon (GCG, blue), F4/80 (yellow; macrophages) and Hoechst (white; nuclei). Scale bar: 25 µm.

(B) Quantification of $F4/80^+$ cells located within or in direct contact with the graft area (INS^+ + GCG^+ areas) at day 30 post-transplantation in control and NaW-treated mice. Data are presented as mean ± SEM; each dot represents the mean value from an individual graft. Statistical analysis was performed using an unpaired two-tailed Student's t-test. No significant differences were observed.

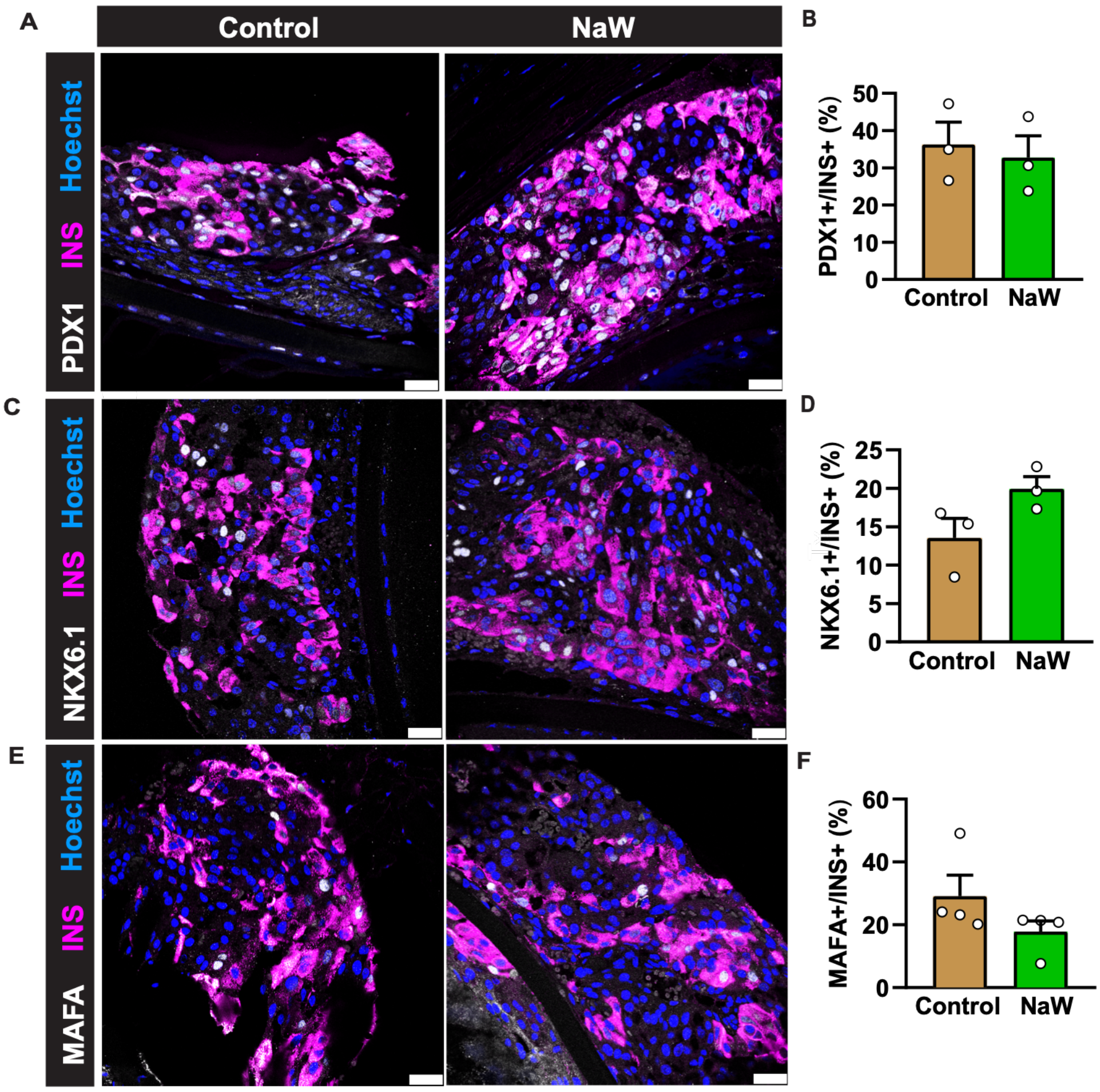

Appendix Figure S7. Histological characterization of beta cells in SC-islet grafts at day 30 post-transplantation.

(A,C,E) Representative immunofluorescence images of SC-islet grafts at day 30 post-transplantation in control and NaW-treated mice. Grafts were stained for the beta cell factors PDX-1 (**A**), NKX6.1 (**C**) or MAFA (**E**) (white: nuclei), insulin (INS, magenta), and Hoechst (blue; nuclei). Scale bar: 25 µm.

(B,D,F) Quantification of the percentage of cells co-expressing INS and PDX1 **(B)**, NKX6.1 **(D)** and MAFA+ **(F)** at day 30 post-transplantation in control and NaW-treated mice. Percentages are calculated relative to the total number of INS^+ cells counted across multiple sections per graft. Data are presented as mean ± SEM; each dot represents the mean value from an individual graft. Statistical analysis was performed using an unpaired two-tailed Student's t-test. No statistical significances were detected between groups.

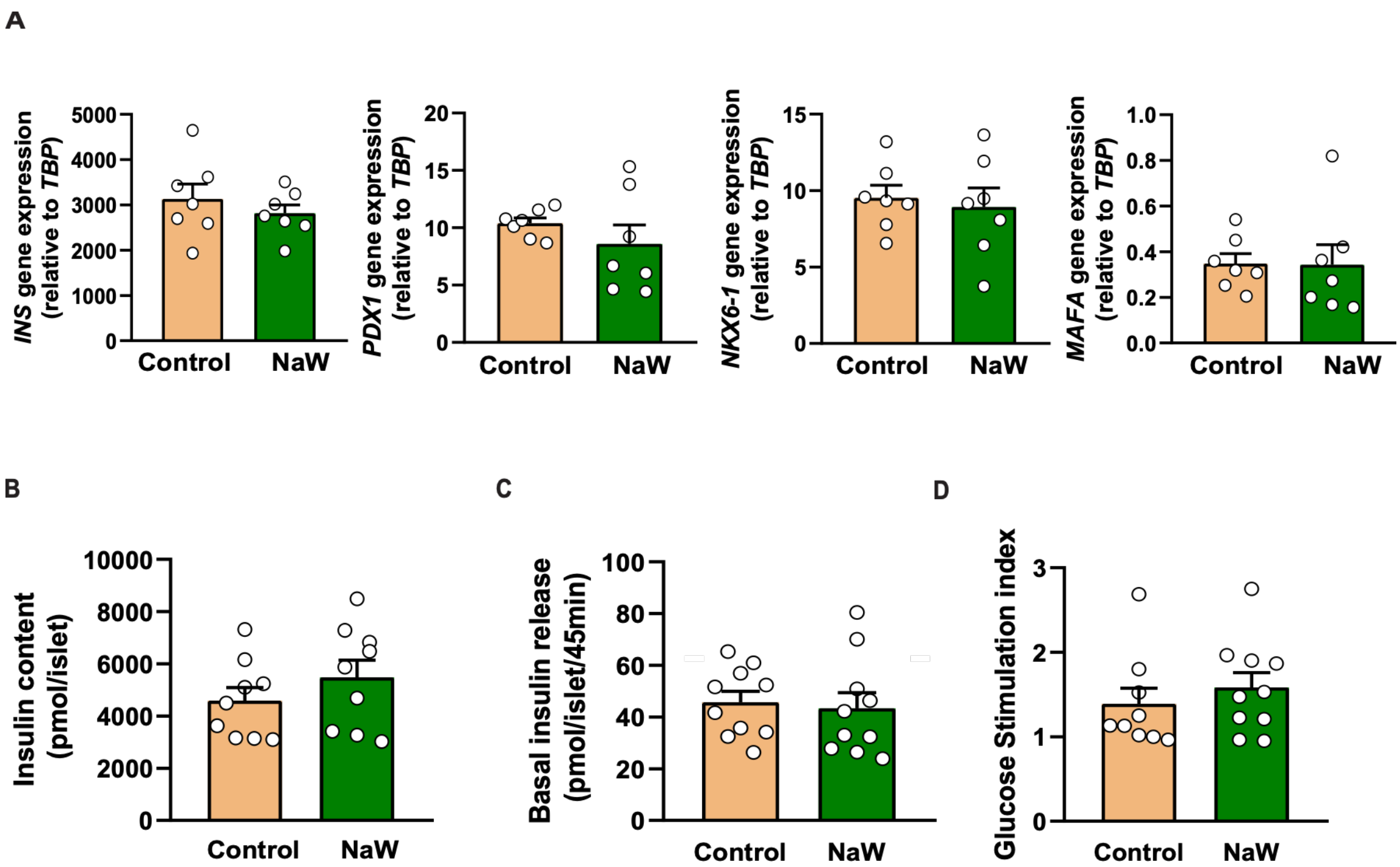

Appendix Figure S8. Effects of NaW on SC-islets *in vitro.*

SC-islets were cultured for 24 hours in the presence of NaW (0.2mM). At the end of this period, RNA was extracted for gene expression quantification and static glucose-induced insulin secretion was performed. Insulin was quantitated by ELISA**.**

(A) qPCR results for the indicated genes.

(B) Insulin protein content following acid alcohol extraction.

(C) Secreted insulin at low glucose (2 mM)

(D) Glucose stimulation Index (fold-change in insulin secretion at 20mM versus 2mM glucose).

Data are presented as mean ± SEM, with each dot representing a biological replicate from n=3 (A) and n=2 (B-D) independent SC-islet differentiations. Statistical analysis was performed using an unpaired two-tailed Student's t-test. No statistical significances were detected between groups.

Appendix Table S1.

Human islet donor information.

Unique identifier	HM102	HM116
Donor age (years)	70	61
Donor sex (M/F)	M	F
Donor BMI (kg/m^2)	24,7	28,5
Donor HbA1c	5.6	5,3
Islet isolation centre	Montpellier	Montpellier
Donor history of diabetes	No	No
Diabetes duration (years)	-	-
Glucose-lowering therapy at time of deathc	-	-
RECOMMENDED INFORMATION		
Donor cause of death	Stroke	Stroke
Warm ischaemia time (h)	<2h	<2h
Cold ischaemia time (h)	11h45	8h40
Estimated purity (%)	85	85
Estimated viability (%)	>80	>80
Total culture time (h)d	3 days	> 2 days
Glucose-stimulated insulin secretion or other functional measuremente	GSIS ratio (16.7 mM vs 2.8mM) = 1,71	?
Handpicked to purity? Please select yes/no from drop down list	?	?
Additional notes	anti-thrombosis / hypertension and anticholesterol treatments	hypertension treatment

Appendix Table S2

Antibody inventory with corresponding techniques.
(IF: immunofluorescence staining in paraffin sections; IT: in toto immunofluorescence staining; WB: immunoblot)

Antibody	Target	Raised in	Working dilution	Commercial source	Technique
**Anti-Insulin**	Human, mouse	Guinea Pig	1/500	Dako	IF
**Anti-Glucagon**	Human, mouse	Mouse	1/250	Invitrogen	IF
**Anti-HLA Class 1 ABC**	Human	Mouse	1/100	Abcam	IF
**Anti-C-Peptide**	Human	Rat	1/300	Hybridoma Bank	IF, IT
**Anti-Ki67**	Human, mouse	Rabbit	1/200	Invitrogen	IF
**Anti-CD34**	Mouse	Rat	1/150	eBioscience	IF
**Anti-CD34**	Human	Sheep	1/20	Biotechne	IF
**Anti-veCADHERIN**	Human, mouse	Rabbit	1/150	Abcam	IF, IT
**Anti-VEGF**	Human, mouse	Rabbit	1/200	Millipore	IF
**Anti-Pdx1**	Human	Rabbit	1/250	Abcam	IF
**Anti-Nkx6.1**	Human., mouse	Mouse	1/50	Hybridoma Bank	IF
**Anti-MafA**	Human, mouse	Rabbit	1/75	Novus Biologicals	IF
**Anti-P-p44/42 MAPK (T202/Y204)**	Human, mouse	Rabbit	1/1000	Cell Signaling	WB
**Anti-p44/42 MAP Kinase**	Human, mouse	Rabbit	1/1000	Cell Signaling	WB
**AlexaFluor 488**	Rabbit	Donkey	1/250	Jackson Immunoresearch,	IF
**AlexaFluor 488**	Guinea Pig	Donkey	1/250	Jackson Immunoresearch,	IF
**Cy2**	Rat	Donkey	1/250	Jackson Immunoresearch,	IF
**Cy2**	Sheep	Donkey	1/250	Jackson Immunoresearch,	IF
**AlexaFluor 555**	Mouse	Donkey	1/400	Molecцlar Probes	IF
**AlexaFluor 555**	Rabbit	Donkey	1/400	Molecцlar Probes	IF, IT
**AlexaFluor 647**	Rat	Donkey	1/250	Jackson Immunoresearch	IF, IT
**AlexaFluor 647**	Rabbit	Donkey	1/250	Jackson Immunoresearch	IF
**Anti-IgG Peroxidase**	Mouse	Sheep	1/5000	GE Healthcare	WB
**Anti-IgG Peroxidase**	Rabbit	Donkey	1/5000	GE Healthcare	WB

Appendix Table S3

List of primers used for gene expression analysis.

Gene		Sequences
TBP	Forward<br>Reverse	5’ - ATCCCTCCCCCATGACTCCCATG -3’<br>5’ - ATGATTACCGCAGCAAACCGC -3’
INS	Forward<br>Reverse	5’ - GCAGCCTTTGTGAACCAACA -3’<br>5’ - TTCCCCGCACACTAGGTAGAGA
NKX6-1	Forward<br>Reverse	5’ - ACACGAGACCCACTTTTTCCG -3’<br>5’ - GCCCCGCCAAGTATTTTGTT -3’
PCSK1	Forward<br>Reverse	5’ - AAGCAAACCCAAATCTCACCTGGC -3’<br>5’ - TCACCATCAAGCCTGCTCCATTCT -3’
PCSK2	Forward<br>Reverse	5’ - CCGGGTTCCTCTTCTGTGTC -3’<br>5’ - AGCAAAGGGAAGCTTTCGGA-3’
KCNJ11	Forward<br>Reverse	5’ - TGTGTCACCAGCATCCACTC -3’<br>5’ - CACTTGGACCTCAATGGAGAA -3’
ABCC8	Forward<br>Reverse	5’ - AGACCCTCATGAACCGACAG -3’<br>5’ - GGCTCTGTGGCTTTTCTCTC -3’
TWIST2	Forward<br>Reverse	5’ - CGCAAGTGGAATTGGGATGC -3’<br>5’ - CGATGTCACTGCTGTCCCTT -3’
CDH5	Forward<br>Reverse	5’ - ATGCGGCTAGGCATAGCATT -3’<br>5’ - TGTGACTCGGAAGAACTGGC -3’
VEGFA	Forward<br>Reverse	5’ - AGTCCAACATCACCATGCAG -3’<br>5’ - TTCCCTTTCCTCGAACTGATTT -3’
MAFA	Forward<br>Reverse	5’ - CTTCAGCAAGGAGGAGGTCA -3’<br>5’ - TTGTACAGGTCCCGCTCTTT -3’
PDX1	Forward<br>Reverse	5’ - GATTGGCGTTGTTTGTGGCT -3’<br>5’ - GCCGGCTTCTCTAAACAGGT -3’
