# Supplementary material for: Sodium tungstate promotes vascularization to support beta cell replacement in diabetes
Source: EMBO Mol Med. 2026 Sep 23;18(11):4334–56. doi: 10.1038/s44321-026-00523-4 (PMC13748639; doi:10.1038/s44321-026-00523-4)
Supplement: Supplementary file 2 [file 44321_2026_523_MOESM2_ESM.pdf]

EMBO Molecular Medicine

Sodium tungstate promotes vascularization to support beta cell replacement in diabetes

Ainhoa García-Alamán, Marta Fontcuberta-PiSunyer, Jonna Saarimäki-Vire, Nea Asumaa, Marta Perea-Atienzar, Rebeca Fernandez-Ruiz, Hugo Alves-Figueiredo, Christophe Broca, Joan-Marc Servitja, Ramon Gomis, Diego Balboa, Josep Vidal, and Rosa Gasa

Corresponding author: Rosa Gasa (rgasa@recerca.clinic.cat)

Review Timeline:

Submission Date:	16th Mar 26
Editorial Decision:	22nd Apr 26
Revision Received:	15th Jul 26
Editorial Decision:	30th Jul 26
Revision Received:	24th Aug 26
Accepted:	28th Aug 26

Editor: Lise Roth

Transaction Report:

(Note: With the exception of the correction of typographical or spelling errors that could be a source of ambiguity, letters and reports are not edited. Depending on transfer agreements, referee reports obtained elsewhere may or may not be included in this compilation. Referee reports are anonymous unless the Referee chooses to sign their reports.)

22nd Apr 2026

Dear Dr. Gasa,

Thank you for submitting your manuscript to our editorial office, and please accept my apologies for the delay in getting back to you, as one referee needed more time to complete their report. We have now received feedback from the three referees who evaluated your study. As you will see in the reports below, they acknowledge the potential translational interest of the study but also make several recommendations to strengthen the conclusions.

If you feel you can satisfactorily address the points listed by the referees, you may wish to submit a revised version of your manuscript. Please attach a covering letter giving details of the way in which you have handled each of the points raised by the referees. A revised manuscript will once again be subject to review, and we cannot guarantee at this stage that the eventual outcome will be favorable. We expect your revised manuscript within three months. If you anticipate any delay, please contact us.

If you would like to discuss further the reports from the referees, I am available to do so via email or video. Let me know if you are interested in this option.

We are expecting your revised manuscript within three months, if you anticipate any delay, please contact us.

When preparing your revised manuscript, please refer to our guidelines: https://link.springer.com/journal/44321/submission-guidelines#cms-Revised-submissions. We perform an initial quality control of all revised manuscripts before re-review; failure to include requested items will delay the evaluation of your revision.

We require:

1) A .docx formatted version of the manuscript text (including legends for main figures, EV figures and tables). Please make sure that the changes are highlighted to be clearly visible.

2) Individual production quality figure files as .eps, .tif, .jpg (one file per figure). For guidance, download the 'Figure Guide PDF': https://media.springernature.com/original/springer-cms/rest/v1/content/27825798/data/v1.

3) A .docx formatted letter INCLUDING the reviewers' reports and your detailed point-by-point responses to their comments. As part of the EMBO Press transparent editorial process, the point-by-point response is part of the Review Process File (RPF), which will be published alongside your paper.

4) A complete author checklist, which you can download from our author guidelines. Please insert information in the checklist that is also reflected in the manuscript. The completed author checklist will also be part of the RPF.

5) Please note that all corresponding authors are required to supply an ORCID ID for their name upon submission of a revised manuscript.

6) It is mandatory to include a 'Data Availability' section after the Materials and Methods. Before submitting your revision, primary datasets produced in this study need to be deposited in an appropriate public database, and the accession numbers and database listed under 'Data Availability'. Please remember to provide a reviewer password if the datasets are not yet public.

In case you have no data that requires deposition in a public database, please state so in this section. Note that the Data Availability Section is restricted to new primary data that are part of this study.

7) For data quantification: please specify the name of the statistical test used to generate error bars and P values, the number (n) of independent experiments (specify technical or biological replicates) underlying each data point and the test used to calculate p-values in each figure legend. The figure legends should contain a basic description of n, P and the test applied. Graphs must include a description of the bars and the error bars (s.d., s.e.m.).

8) At EMBO Press we ask authors to provide source data for the main manuscript figures. You will receive a separate email with instructions for providing source data with your revised manuscript, including how to upload and organize the files.

9) Our journal encourages inclusion of *data citations in the reference list* to directly cite datasets that were re-used and obtained from public databases. Data citations in the article text are distinct from normal bibliographical citations and should directly link to the database records from which the data can be accessed. In the main text, data citations are formatted as follows: "Data ref: Smith et al, 2001" or "Data ref: NCBI Sequence Read Archive PRJNA342805, 2017". In the Reference list, data citations must be labeled with "[DATASET]". A data reference must provide the database name, accession

number/identifiers and a resolvable link to the landing page from which the data can be accessed at the end of the reference.

10) We replaced Supplementary Information with Expanded View (EV) Figures and Tables that are collapsible/expandable online. A maximum of 5 EV Figures can be typeset. EV Figures should be cited as 'Figure EV1, Figure EV2" etc... in the text and their respective legends should be included in the main text after the legends of regular figures.

- For the figures that you do NOT wish to display as Expanded View figures, they should be bundled together with their legends in a single PDF file called *Appendix*, which should start with a short Table of Content. Appendix figures should be referred to in the main text as: "Appendix Figure S1, Appendix Figure S2" etc.

- Additional Tables/Datasets should be labeled and referred to as Table EV1, Dataset EV1, etc. Legends have to be provided in a separate tab in case of .xls files. Alternatively, the legend can be supplied as a separate text file (README) and zipped together with the Table/Dataset file.

11) The paper explained: EMBO Molecular Medicine articles are accompanied by a summary of the articles to emphasize the major findings in the paper and their medical implications for the non-specialist reader. Please provide a draft summary of your article highlighting
- the medical issue you are addressing,
- the results obtained and
- their clinical impact.

This may be edited to ensure that readers understand the significance and context of the research. Please refer to any of our published articles for an example.

12) Author contributions: You will be asked to provide CRediT (Contributor Role Taxonomy) terms in the submission system. These replace a narrative author contribution section in the manuscript.

13) A "Disclosure and Competing Interests Statement" should be provided in the main text.

14) Every published paper includes a 'Synopsis' to further enhance discoverability. Synopses are displayed on the journal webpage and are freely accessible to all readers. They include a short stand first (maximum of 300 characters, including space) as well as 2-5 one-sentences bullet points that summarizes the paper. Please write the bullet points to summarize the key NEW findings. They should be designed to be complementary to the abstract - i.e. not repeat the same text. We encourage inclusion of key acronyms and quantitative information (maximum of 30 words / bullet point). Please use the passive voice. Please attach these in a separate file or send them by email, we will incorporate them accordingly.

Please also suggest a striking image or visual abstract to illustrate your article as a PNG file 550 px wide x 300 px high.

15) Include a Reagents and Tools Table as part of the Methods section, which can be downloaded from our author guidelines.

16) As part of the EMBO Publications transparent editorial process initiative (see our Editorial at http://embomolmed.embopress.org/content/2/9/329), EMBO Molecular Medicine will publish online a Review Process File (RPF) to accompany accepted manuscripts.
In the event of acceptance, this file will be published in conjunction with your paper and will include the anonymous referee reports, your point-by-point response and all pertinent correspondence relating to the manuscript. Let us know whether you agree with the publication of the RPF and as here, if you want to remove or not any figures from it prior to publication.
Please note that the Authors checklist will be published at the end of the RPF.

EMBO Molecular Medicine has a "scooping protection" policy, whereby similar findings that are published by others during review or revision are not a criterion for rejection. Should you decide to submit a revised version, I do ask that you get in touch after three months if you have not completed it, to update us on the status.

I look forward to receiving your revised manuscript.

Yours sincerely,

Lise Roth

Lise Roth, PhD
Senior Editor
EMBO Molecular Medicine

***** Reviewer's comments *****

Referee #1 (Remarks for Author):

The Paper of Gasa and colleagues shows that sodium tungstate NaW can improve vascularization in transplanted insulin-producing cell clusters and thereby support cell survival. Particularly strong are the use of two model systems, the mechanistic analysis through VEGFA, and the translational relevance for diabetes therapies.
The study provides interesting evidence that NaW can enhance vascularization in transplanted cell clusters, but the use of a relatively high concentration of 210 µM raises questions about clinical translatability. Although the authors refer to prior safety data and argue that the compound is well tolerated, it remains unclear whether such a dose is truly necessary or whether lower concentrations could achieve similar effects with less risk. the authors should perform a dose curve showing that lower concentrations are efficient / not efficient ?
A further limitation is that the work focuses mainly on early vascular and histological outcomes, while potential dose-dependent side effects, off-target effects, and long-term consequences of systemic administration are not addressed in sufficient depth. For a broadly acting phosphatase inhibitor, a more rigorous dose-response analysis would be important to better balance efficacy and safety. The authors should at least discuss this issue.

Referee #2 (Remarks for Author):

The authors show that sodium tungstate (NaW), a broad phosphatase inhibitor (PTP) improved vascularization in human fibroblast-derived insulin-producing cells and human stem cell-derived islets transplanted into the anterior chamber of the eye in immunodeficient mice. Mechanistically, it is shown that NaW upregulates P-ERK in HUVECs.
While it is clinically highly important to improve vascularization of transplanted stem-cell derived beta-cells, and NaW is a potential strategy, I have some further questions for better understanding of the data.

Fig.1B shows HUVECs preferentially localizing to the spheroid core in a ring-like
arrangement, while insulin-positive (INS^+) cells were predominantly found at the periphery.
I don't really understand this well, as there is no "periphery" shown. There is a patchy staining of insulin in blue, but it is unclear what it is. Additional DAPI label or similar would probably show the cells better.

In Fig.1F-H, analysis is shown after graft removal; while vascularization has shown to be increased, TUNEL remains unchanged among differentiated cells.
It would be great to provide representative pictures of the analysis 1F-H
Why was TUNEL not correlated to INS+ (1F)?
HLA+/CD31- was used for normalization. Was HLA itself changed in transplanted cells?
How was vascularization in the graft ex-vivo= e.g. lectin, VE-cadherin, CD31?
Does improved vascularization come with more inflammation? Can authors also stain for an inflammation marker or macrophages in the graft?
How are iBeta-like cells defined (1H)?
The Glucose Stimulation Index from S1E should be moved to Fig.1, and basal and stimulated insulin secretion and content should also be shown.

Fig.2
Does NaW now have an additive effect on vascularization, e.g. what are the effects of HUVECs (shown in Fig.1)/NaW alone?
It would be much better to combine the same analysis with those in Fig.1; there are some differences; in 1F, TUNEL is 4%, in 2F only 2.5%, and here, analysis is shown in INS+ cells is shown.
In 2I,J; how is graft in controls shown relative to control? What is the difference? Probably this needs better explanation of the normalization strategy.

Fig.4
It is shown that NaW upregulates P-ERK in HUVECs. Authors describe that NaW
amplifies VEGFA-induced endothelial cell proliferation,
migration, and tubulogenesis via MAPK/ERK signalling.
Data show that effect on mesh, nodes and ERK is independent on VEGFA, i.e. in absence of VEGFA, the NaW effect is

stronger.
No n for westerns given in 4H,I
Why only mechanism for HUVECs and not mechanistic analysis in grafts, i.e. proof whether NaW was specific for PTP?

Fig.5; it is difficult to assess the contribution of the effect of NaW in the graft on inulin secretion. What is the effect of NaW on insulin secretion without transplantation? As it is described in methods, can authors show both human and mouse insulin secretion at basal, stimulated, content and index?
Inflammation markers should be shown in the grafts, authors could add some beta-cell identity/functional markers and inflammation markers in their PCR analysis.

Referee #3 (Remarks for Author):

This is an interesting paper that explores the potential use of sodium tungstate (NaW) to enhance VEGFA production and improve islet graft vascularization. Human fibroblast derived insulin producing cells and human stem cell derived islets were transplanted into the anterior chamber of the eye in immunodeficient mice. NaW treatment increased both vascularized area and insulin containing tissue, decreased apoptosis and these effects were associated with increases in VEGFA expression. It is suggested that some of the actions of this known phosphatase inhibitor are due to modifications of the ERK pathway. Last, evidence presented indicating that the pro-angiogenic effects are independent of exogenous endothelial cell supplementation and are the result of host endogenous endothelial cells. The authors conclude that the use of NaW or other phosphatase inhibitors may provide a viable strategy to improve vascularization and survival of cell-based transplantation protocols.
Overall, this is a descriptive study that associates improvements in vascularization and survival of islet like clusters in an immune privileged site (ACE) with an inexpensive potential therapeutic shown to be well tolerated and safe in multiple studies. While descriptive in nature, there is novelty in identifying effects on host endothelial cell vascularization in the transplanted clusters, the lack of effects of combining islet cells with endothelial cells prior to transplant. The caveats of this work are well described, and methods appear appropriate for the studies performed. Additional detail on off target actions of NaW, including metabolic enzyme activity would be helpful as this compound is known to inhibit gluconeogenic enzymes, modify glycogen metabolism, alter lipid metabolism, in addition to PTP1B inhibition.
Specific areas that could be addressed include:
1. Insulin secretion assays show stimulation index. This index does not provide information on basal insulin release, which could be modified when treating with a phosphatase inhibitor. Were there differences between basal insulin release between the treatment groups in Fig S1D. Note that in Fig 5, plasma human insulin levels were increased in NaW treated mice, yet the stimulation index was not modified.
2. NaW was provided in the drinking water, were consumption levels of NaW monitored? While others have used this dose, are there positive controls that could be performed to ensure that the inhibitor concentrations are sufficient to attenuate phosphatase activity in the desired tissues?
3. Functional vascularization was evaluated at a single timepoint, day 10. Were there effects are earlier times post-transplant? It is possible that at this single time point changes in vascularization were missed. This is also the case for proliferation rates. Also, is there a reason that proliferation was not assessed using BrDU vs cell counting?
4. The authors correlate increases in VEGFA production by ibeta like cells with NaW treatment, but suggest it is a direct effect. This study was done at a single time point of 48 h of treatment and at high concentrations of the drug. What is the time-dependence of NaW treatment of ibeta like clusters on VEGFA mRNA accumulation and how does this correlate with release?

***** Reviewer's comments *****

Referee #1 (Remarks for Author):

The Paper of Gasa and colleagues shows that sodium tungstate NaW can improve vascularization in transplanted insulin-producing cell clusters and thereby support cell survival. Particularly strong are the use of two model systems, the mechanistic analysis through VEGFA, and the translational relevance for diabetes therapies.
The study provides interesting f that NaW can enhance vascularization in transplanted cell clusters, but the use of a relatively high concentration of 210 µM raises questions about clinical translatability. Although the authors refer to prior safety data and argue that the compound is well tolerated, it remains unclear whether such a dose is truly necessary or whether lower concentrations could achieve similar effects with less risk. the authors should perform a dose curve showing that lower concentrations are efficient / not efficient?

Response: We thank the reviewer for raising this important point about dose and clinical translatability.

On safety: the acute oral LD50 in mice is approximately 1904 mg/kg (doi: 10.1177/1091581815585568, cited in the manuscript), and our estimated intake of ~125 mg/kg/day represents only ~6.6% of this value. The National Toxicology Program's two-year study (NTP TR-599, 2021) found no evidence of carcinogenicity or genotoxicity at concentrations up to 1000-2000 mg/L, and several independent studies in standard mouse strains report no overt toxicity at comparable doses (doi: 10.1021/tx200011k; doi: 10.3109/1547691X.2016.1154118), all cited in the paper.

We acknowledge that demonstrating efficacy at our chosen dose doesn't establish the minimum effective dose. In this regard, we can note that in separate, ongoing work, we have obtained unpublished preliminary data using half the standard dose (0.5 g/L) in diabetic NSG recipients transplanted with iBeta-like/HUVEC spheroids, showing comparable pro-vascularization effects, with one parameter narrowly missing statistical significance (Reviewer Figure R1). While this data is not part of the current manuscript, it provides preliminary evidence that a lower dose may retain efficacy, albeit potentially with a smaller effect size. Given the consistency of the observed effects and the substantial preclinical evidence supporting the safety and validity of the 1 g/L dose, we respectfully feel that repeating the full experimental battery at a lower dose is not feasible within the scope of the current study.

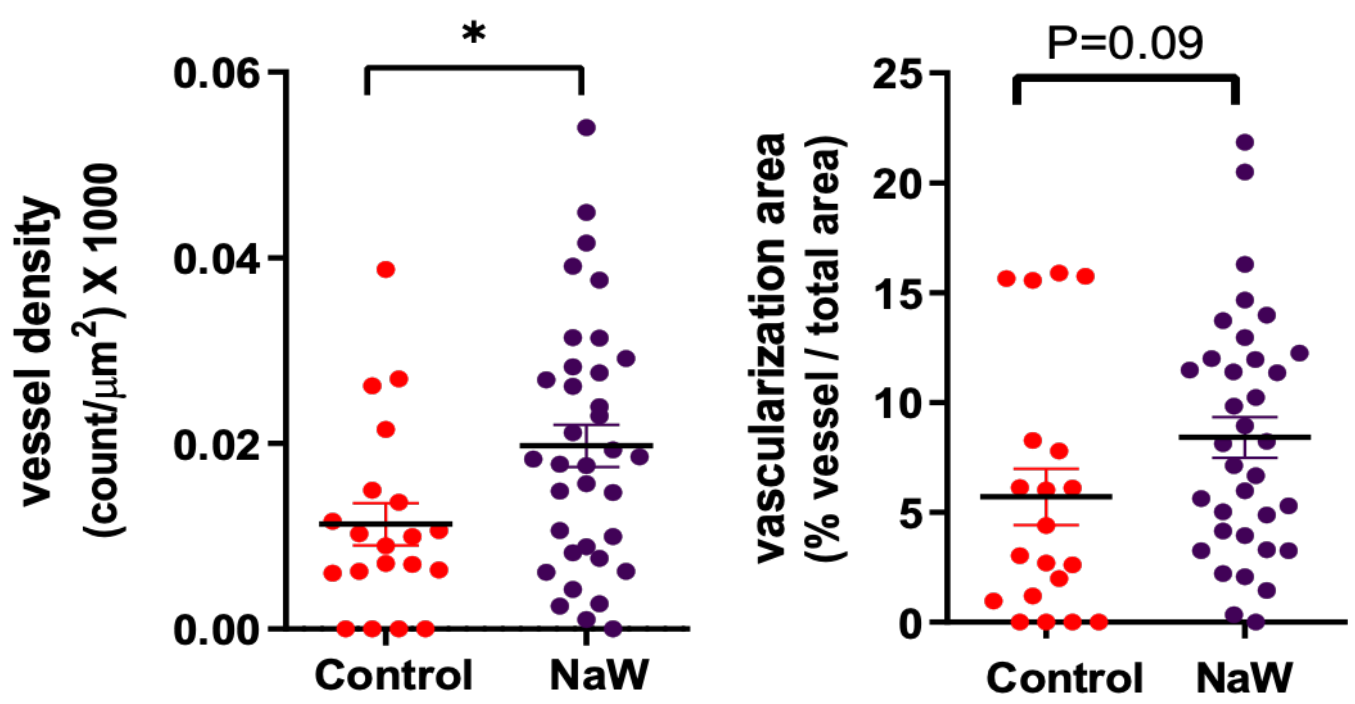

Figure R1. Quantitative analysis of vessel density and vascularized area in grafts at day 10 post-transplantation in diabetic NSG mice treated with 0.5 g/L NaW in the drinking water. Data are presented as mean ± SEM, with the number of observations indicated by individual dots. Each dot corresponds to one measured area from a total of 3 (control) and 5 (NaW) grafts. Statistical significance was determined using an unpaired Student's t-test: *P < 0.05.

A further limitation is that the work focuses mainly on early vascular and histological outcomes, while potential dose-dependent side effects, off-target effects, and long-term consequences of systemic administration are not addressed in sufficient depth. For a broadly acting phosphatase inhibitor, a more rigorous dose-response analysis would be important to better balance efficacy and safety. The authors should at least discuss this issue.

Response: We thank the reviewer for this constructive comment. The primary objective of the present study was to evaluate early vascular and histological outcomes following sodium tungstate administration, and we agree that a more comprehensive dose-response and long-term toxicological analysis would strengthen the translational case for this approach. As noted in our response to the previous comment, we have preliminary unpublished data from a lower-dose pilot experiment supporting that lower doses may retain efficacy; a full dose-response and long-term safety study, however, was beyond the scope of the current work.

Throughout the 30-day treatment period, no adverse effects were observed, including no weight loss or deterioration of general condition in any of the experimental animals. Regarding the safety profile of the dose employed, we have now moved the relevant toxicological references, including the National Toxicology Program Technical Report TR-599 (2021), from the Methods section to the Introduction of the revised manuscript, where we believe they will be more visible and provide clearer justification for the dose selection at the beginning of the paper.

We also acknowledge that prior toxicological studies were not conducted in NSG mice, and therefore strain-specific long-term effects cannot be entirely excluded. We have addressed this point more explicitly in the last paragraph of the Limitations section of the revised manuscript. We hope that these clarifications adequately address the reviewer's concerns.

Referee #2 (Remarks for Author):

The authors show that sodium tungstate (NaW), a broad phosphatase inhibitor (PTP) improved vascularization in human fibroblast-derived insulin-producing cells and human stem cell-derived islets transplanted into the anterior chamber of the eye in immunodeficient mice. Mechanistically, it is shown that NaW upregulates P-ERK in HUVECs. While it is clinically highly important to improve vascularization of transplanted stem-cell derived beta-cells, and NaW is a potential strategy, I have some further questions for better understanding of the data.

Fig.1B shows HUVECs preferentially localizing to the spheroid core in a ring-like arrangement, while insulin-positive (INS^+) cells were predominantly found at the periphery. I don't really understand this well, as there is no "periphery" shown. There is a patchy staining of insulin in blue, but it is unclear what it is. Additional DAPI label or similar would probably show the cells better.

Response: We have prepared an updated figure layout that includes separate images for INS and CDH5 staining, in addition to a merged image incorporating the nuclear staining layer (displayed in white) to enhance clarity and interpretation. The spheroid boundary is also delineated to further improve visual clarity and interpretation.

In Fig.1F-H, analysis is shown after graft removal; while vascularization has shown to be increased, TUNEL remains unchanged among differentiated cells. It would be great to provide representative pictures of the analysis 1F-H. Why was TUNEL not correlated to INS+ (1F)?

Response: We have re-analysed the TUNEL images at days 2 and 10 to quantify the percentage of INS^+ cells (now presented as inset of Panel H in revised Fig. 1). The analysis shows no significant differences between spheroids composed of iBeta-like cells alone and those combined with HUVEC cells at either time point.

HLA+/CD31- was used for normalization. Was HLA itself changed in transplanted cells?

Response: HLA expression was not observably altered following transplantation within the 30-day experimental window, and we have not detected any evident decline in this marker over this period in our laboratory. Anti-HLA immunostaining is a well-established method for identifying human cells in murine xenotransplantation models. Representative post-explant images showing HLA, INSULIN and hCD34 staining have been included in the revised manuscript (Fig. 1F). These images support the quantification data in Fig. 1G, showing that the inclusion of HUVEC cells does not affect the reprogramming rate in spheroids.

How was vascularization in the graft ex-vivo= e.g. lectin, VE-cadherin, CD31? Does improved

vascularization come with more inflammation? Can authors also stain for an inflammation marker or macrophages in the graft?

Response: We thank the reviewer for this important question. In the revised manuscript, we have performed additional analyses in the transplantation experiments that included a NaW treatment arm (as these represent the experiments most central to the study's main findings on graft survival). This excludes the iBeta-like ± HUVEC comparison shown in Figure 1, which did not include NaW treatment.

To evaluate vascularization, we performed immunofluorescence staining for the endothelial marker CD34 on retrieved iBeta-like/HUVEC and SC-islet grafts. This confirmed an increase in the number of $CD34^+$ vascular structures following NaW treatment in both models (Figures EV2 and EV3 of the revised manuscript). We also provide new data showing that the proportion of HUVEC cells in iBeta-like/HUVEC transplants is not affected by NaW treatment, pointing to host mouse endothelial cells as responsible for the increased vascularization. In SC-islet grafts we additionally observed a reduced calibre of intragraft vessels in NaW-treated animals relative to controls, consistent with active angiogenesis.

To evaluate the inflammatory response, we quantified macrophage infiltration (F4/80+ cells per graft area) in both SC-islet and iBeta-like /HUVEC cell grafts. These new data are provided in Figures S3 and S6 of the revised manuscript. SC-islets showed low macrophage content that remained largely unchanged following treatment. On the other hand, iBeta-like/HUVEC cell grafts displayed markedly higher baseline macrophage infiltration with no-significant change in NaW-treated mice. Notably, both models showed comparable increases in vascularization (Figures 2,5, EV2, EV3) despite this difference in baseline macrophage content, suggesting that the elevated macrophage infiltration observed in reprogrammed cell grafts reflects a cell-intrinsic property of this model rather than a generalized inflammatory consequence of NaW's pro-angiogenic activity. This may relate to residual fibroblast characteristics of the reprogrammed cells, including their secretory profile or extracellular matrix composition, which could promote innate immune cell recruitment independently of vascularization. The precise basis of this property is beyond the scope of this study, but is being investigated in ongoing work.

How are iBeta-like cells defined (1H)?

Response: Cells with human INSULIN and HLA-positive staining in 30-day grafts.

The Glucose Stimulation Index from S1E should be moved to Fig.1, and basal and stimulated insulin secretion and content should also be shown.

Response: We thank the reviewer for this suggestion. The insulin secretion experiments were performed on iBeta-like cell clusters at the end of the reprogramming protocol, prior to transplantation. We therefore consider these data part of the *in vitro* characterization of the cells, alongside other analyses such as cell

survival, reprogramming efficiency, and gene expression, and have kept them together in this section of the revised manuscript.

To address the reviewer's comment, we have added a new panel showing basal and glucose-stimulated insulin secretion (Figure EV1E), alongside the glucose stimulation index (Figure EV1F), which was previously derived from these measurements and shown in Figure S1E of the original submission.

As Figure 1 is dedicated to the *in vivo* transplantation experiments, we felt it was clearer to keep the *in vitro* characterization data, including insulin secretion, together in Figure EV1 rather than splitting it across two figures. We hope the reviewer agrees this maintains a clearer distinction between the *in vitro* and *in vivo* datasets, but we are happy to reconsider if the reviewer feels strongly that this panel should appear in Figure 1.

Fig.2

Does NaW now have an additive effect on vascularization, e.g. what are the effects of HUVECs (shown in Fig.1)/NaW alone? It would be much better to combine the same analysis with those in Fig.1; there are some differences; in 1F, TUNEL is 4%, in 2F only 2.5%, and here, analysis is shown in INS+ cells is shown.

Response: We thank the reviewer for this important question.

Our study was designed sequentially: we first validated that HUVEC co-transplantation improves vascularization of iBeta-like grafts (Figure 1), and built on this established system to next test whether NaW treatment could further enhance vascularization (Figure 2). This sequential design is also the reason we have kept these datasets in separate figures rather than integrating them into a single analysis, as the experiments were conducted as distinct, independently controlled cohorts rather than as a single combined study. As such, the present experimental design does not include a NaW-alone (without HUVEC) arm within the iBeta-like model, and we cannot formally test for an additive or interactive effect between NaW and HUVEC co-transplantation in this system.

Regarding the differences in TUNEL values between Figures 1 and 2, we consider this degree of variation (e.g., iBeta-like/HUVEC spheroids, day 10, $TUNEL^+HLA^+$ of 1.67% versus 2.49%, or $TUNEL^+$ INS^+ of 0.66% versus 0.43%) to be within normal range of biological variability expected between independently performed in vivo cohorts

In 2I,J; how is graft in controls shown relative to control? What is the difference? Probably this needs better explanation of the normalization strategy.

Response: Briefly, all INS^+ cells across multiple sections throughout the retrieved eye were summed for each animal. To account for the inherent biological variability (i.e. differences in the number of cells successfully injected into ACE) and the number of cells remaining in 30-day grafts, individual values were expressed relative to the mean of the untreated control group, which was arbitrarily set to 100%. This approach allows meaningful comparison between groups while accounting for inter-animal variability,

and is appropriate given that absolute cell numbers cannot be reliably normalized by any other reference in this model.

Fig.4. It is shown that NaW upregulates P-ERK in HUVECs. Authors describe that NaW amplifies VEGFA-induced endothelial cell proliferation, migration, and tubulogenesis via MAPK/ERK signalling. Data show that effect on mesh, nodes and ERK is independent on VEGFA, i.e. in absence of VEGFA, the NaW effect is stronger.

No n for westerns given in 4H,I

Response: Total number of westerns was 4 corresponding to 4 independent experiments. For each experiment, n=2 lanes corresponding to replicates (different wells in the same plate).

Why only mechanism for HUVECs and not mechanistic analysis in grafts, i.e. proof whether NaW was specific for PTP?

Response: We appreciate the reviewer's comment. However, mechanistic analyses in transplanted grafts are not straightforward. To formally demonstrate that the observed effects are mediated by PTP inhibition, additional genetic approaches would be required, including the generation of β-like cells or SC-islets deficient in PTP1B, and potentially other PTPs, prior to transplantation and/or the use of PTP-deficient recipient animals. While we agree that such studies would provide valuable mechanistic insight, they are beyond the scope of the present work and not feasible within the timeframe of this revision.

Nevertheless, this is an important question that we are currently pursuing. We are investigating the contribution of PTP1B, as well as other PTPs, to the effects of NaW using *in vitro* approaches and plan to subsequently extend these studies to *in vivo* transplantation models. We acknowledge this limitation in the revised manuscript and have discussed it accordingly in the Limitations section.

Fig.5; it is difficult to assess the contribution of the effect of NaW in the graft on inulin secretion. What is the effect of NaW on insulin secretion without transplantation? As it is described in methods, can authors show both human and mouse insulin secretion at basal, stimulated, content and index?

Response: We agree with the reviewer that, *in vivo*, it is difficult to distinguish whether the increased basal human insulin secretion observed in NaW-treated animals results from enhanced insulin secretion at the level of individual β-cells or from an increase in total β-cell mass. To address this question, we performed additional *in vitro* experiments to evaluate whether NaW exerts direct effects on SC-islets. Specifically, we assessed β-cell marker gene expression by qPCR, insulin content, basal insulin release and the glucose stimulation index (for comparison with *in vivo* data). We observed no significant changes in any of these parameters, suggesting that NaW does not directly affect β-cell identity, insulin content, or secretory function under the tested *in vitro* conditions (Appendix Figure S8). Given these findings and the

observed increase in graft area (Figure 5F), we propose that the elevated basal insulin release in NaW-treated animals is more likely attributable to an increased number of β-cells rather than enhanced secretory activity per cell.

Regarding the reviewer's request to present both human and mouse insulin data, we note that the study was designed to assess graft-derived human insulin secretion. Mouse insulin measurements were not included in the original study design, and no plasma samples remain available for additional analyses. Therefore, we are unable to provide mouse insulin secretion data.

Nonetheless, to address the reviewer's concern regarding the effects of NaW in the absence of transplantation, we refer to independent experiments performed in our laboratory using BALB/c mice treated with sodium tungstate. In these studies, fasting insulin levels were measured and no significant differences were detected between NaW-treated and control animals. These findings further support the conclusion that NaW does not substantially influence β-cell function or insulin secretion directly. We provide these data for the reviewer's consideration (Reviewer Figure R2).

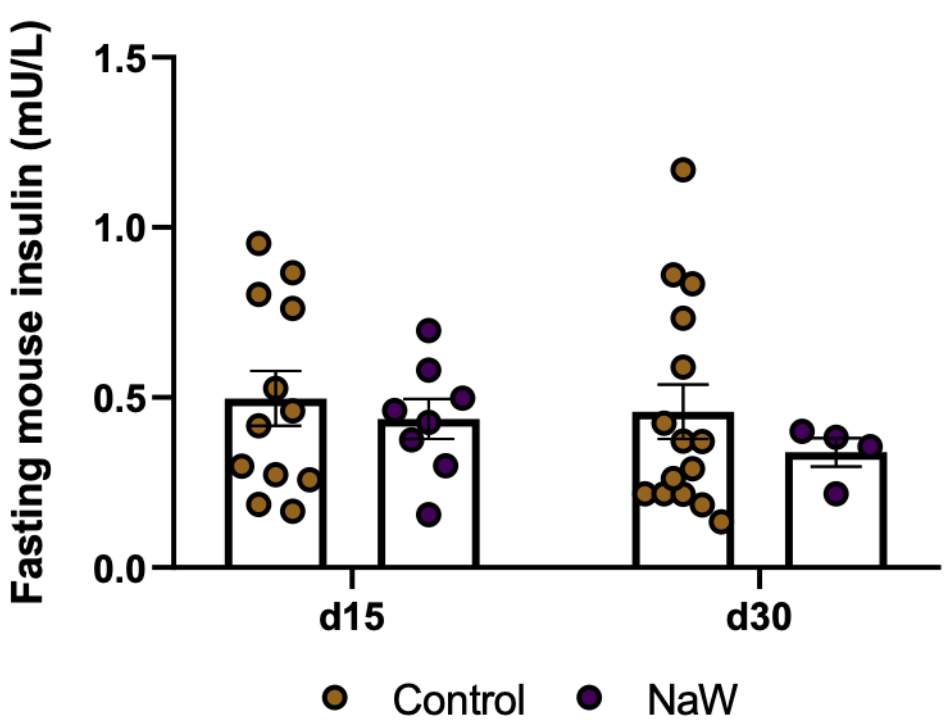

Figure R2. Balb/c mice were treated with sodium tungstate in the drinking water (0.5 g/L) for 15 and 30 days. Plasma mouse insulin was measured after a 5 h fast. Data are presented as mean ± SEM. Each dot corresponds to an individual mouse. No significant differences were observed using two-way ANOVA.

Inflammation markers should be shown in the grafts, authors could add some beta-cell identity/functional markers and inflammation markers in their PCR analysis.

Response: As described in our response to a previous comment (see page 5 of this letter), we quantified macrophage infiltration (F4/80+ cells per graft area) in iBeta-like/HUVEC and SC-islets grafts and found no significant changes following NaW treatment (Figures S3 and S6). This argues against a generalized inflammatory effect of the pro-angiogenic treatment itself.

Referee #3 (Remarks for Author):

This is an interesting paper that explores the potential use of sodium tungstate (NaW) to enhance VEGFA production and improve islet graft vascularization. Human fibroblast derived insulin producing cells and human stem cell derived islets were transplanted into the anterior chamber of the eye in immunodeficient mice. NaW treatment increased both vascularized area and insulin containing tissue, decreased apoptosis and these effects were associated with increases in VEGFA expression. It is suggested that some of the actions of this known phosphatase inhibitor are due to modifications of the ERK pathway. Last, evidence presented indicating that the pro-angiogenic effects are independent of exogenous endothelial cell supplementation and are the result of host endogenous endothelial cells. The authors conclude that the use of NaW or other phosphatase inhibitors may provide a viable strategy to improve vascularization and survival of cell-based transplantation protocols.

Overall, this is a descriptive study that associates improvements in vascularization and survival of islet like clusters in an immune privileged site (ACE) with an inexpensive potential therapeutic shown to be well tolerated and safe in multiple studies. While descriptive in nature, there is novelty in identifying effects on host endothelial cell vascularization in the transplanted clusters, the lack of effects of combining islet cells with endothelial cells prior to transplant. The caveats of this work are well described, and methods appear appropriate for the studies performed. Additional detail on off target actions of NaW, including metabolic enzyme activity would be helpful as this compound is known to inhibit gluconeogenic enzymes, modify glycogen metabolism, alter lipid metabolism, in addition to PTP1B inhibition.

Specific areas that could be addressed include:

1. Insulin secretion assays show stimulation index. This index does not provide information on basal insulin release, which could be modified when treating with a phosphatase inhibitor. Were there differences between basal insulin release between the treatment groups in Fig S1D. Note that in Fig 5, plasma human insulin levels were increased in NaW treated mice, yet the stimulation index was not modified.

Response: We thank the reviewer for bringing up this point. We agree that the stimulation index alone does not provide information regarding basal insulin secretion, which could potentially be affected by phosphatase inhibition. To address this issue, respect to Figure S1D, we now show data on both basal and glucose-stimulated insulin secretion, in addition to the glucose stimulation index, in Figure EV1. These data demonstrate that there are no significant differences in either basal or stimulated insulin secretion between iBeta-like and iBeta-like/HUVEC cells, confirming that the similar stimulation index observed in Figure S1D is not masking differences in basal insulin release.

With respect to Figure 5**,** we indeed observed increased basal human insulin levels in NaW-treated animals (Fig. 5L), whereas the stimulation index remained unchanged (Fig. 5M). We agree that, *in vivo*, it is difficult to distinguish whether this increase reflects enhanced secretory activity of individual β-cells or an increase

in total β-cell mass. To address this question, we performed additional *in vitro* experiments to determine whether NaW directly affects SC-islet function. We assessed β-cell marker gene expression by qPCR, insulin content, as well as insulin release (basal and glucose stimulation index). No significant differences were observed in any of these parameters (Appendix Figure S8), suggesting that NaW does not directly affect β-cell identity, insulin content, or secretory function under the tested *in vitro* conditions. Together with the increased graft area observed in NaW-treated animals (Fig. 5F), these findings support the interpretation that the elevated basal insulin levels are more likely due to an increase in β-cell mass rather than enhanced insulin secretion on a per-cell basis

2. NaW was provided in the drinking water, were consumption levels of NaW monitored? While others have used this dose, are there positive controls that could be performed to ensure that the inhibitor concentrations are sufficient to attenuate phosphatase activity in the desired tissues?

Response: We thank the reviewer for this important comment. Water consumption was monitored throughout the study. On average, mice consumed 3.6 mL of water per day, corresponding to an estimated sodium tungstate dose of approximately 125 mg $NaWO_4 \cdot 2H_2O$/kg body weight/day. We have now included this information in the *"Sodium tungstate treatment"* subsection of the revised Methods section.

Regarding the assessment of target engagement, we acknowledge that direct demonstration of phosphatase inhibition in the graft or recipient tissues would further strengthen the mechanistic interpretation of our findings. However, to the best of our knowledge, there remains a substantial gap in understanding the *in vivo* mechanisms of action of sodium tungstate. Although a metabolic study in diabetic rats reported that sodium tungstate treatment (2 g/L) restored the phosphorylation of several hepatic kinases (doi:10.1371/journal.pone.0042305), most of the available evidence regarding its molecular mechanisms has been obtained from *in vitro* studies.

In the context of our study, formally addressing this question would require the use of genetic models targeting PTP1B and potentially other protein tyrosine phosphatases (PTPs). Such approaches could include the use of PTP-deficient NSG recipient mice and/or the transplantation of PTP-deficient stem cell-derived islets (SC-islets) or iBeta-like cells to directly assess the contribution of these phosphatases to the effects of sodium tungstate. While we agree that these experiments would provide important mechanistic insight, they are beyond the scope and timeframe of the current study. Nevertheless, this is an important area that we are actively pursuing, initially through *in vitro* studies and subsequently in *in vivo* transplantation models. We have explicitly acknowledged this limitation in the Discussion section of the revised manuscript.

3. Functional vascularization was evaluated at a single timepoint, day 10. Were there effects are earlier times post-transplant? It is possible that at this single time point changes in vascularization were missed. This is also the case for proliferation rates. Also, is there a reason that proliferation was not assessed using BrDU vs cell counting?

Response: We assessed an earlier time point (day 4) in our initial experiments using iBeta-like and iBeta-like/HUVEC spheroids, where a clear increase in vascularization was observed between day 4 and days 10-12. Based on these findings, as well as prior experience in our laboratory, we selected day 10 for the NaW experiments, as this time point corresponds to the establishment of functional vascularization and aligns with our interest in evaluating this outcome. Importantly, at this stage we were able to detect differences in response to sodium tungstate; had this not been the case, earlier time points would have been further explored. We also note that, as shown in Fig. 5, functional vascularization at day 15 has not yet reached its maximum and continues to increase up to day 30 in SC-islets.

We did not perform BrdU incorporation assays due to the experimental design, which included dextran infusion to assess functional vascularization, followed by animal sacrifice and graft retrieval. In addition, BrdU staining would have required an additional injection, which was not incorporated into the experimental protocol.

4. The authors correlate increases in VEGFA production by ibeta like cells with NaW treatment, but suggest it is a direct effect. This study was done at a single time point of 48 h of treatment and at high concentrations of the drug. What is the time-dependence of NaW treatment of ibeta like clusters on VEGFA mRNA accumulation and how does this correlate with release?

Response: We thank the reviewer for this question. We selected 48 h as our primary timepoint to ensure sufficient VEGFA accumulation in the medium for reliable ELISA quantification. However, a separate recent experiment performed in the frame of another study showed that 0.4 mM NaW (intermediate concentration) already increased *VEGFA* mRNA at 24 h relative to control (Reviewer Figure R3), suggesting transcriptional induction begins earlier than 48 h. We have not performed a full time-course, so we cannot describe the detailed kinetics of mRNA induction or its relationship to protein secretion; we agree this would be valuable to address in future work.

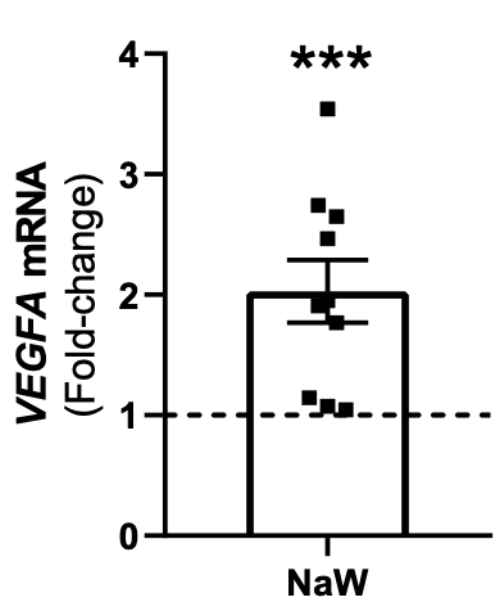

Figure R3. iBeta-like cells were exposed to 0.4mM NaW for 24 h. RNA was extracted and *VEGFA* mRNA levels quantitated by qPCR using *TBP* as housekeeping gene. Graph depicts fold-change *VEGFA* gene expression in NaW-treated cells compared to control cells (given the value of 1, dotted line). Data are mean ± SEM for the number of biological replicates indicated by dots, corresponding to 3 independent reprogramming experiments. P<0.001 using one sample t-test.

30th Jul 2026

Dear Dr. Gasa,

Thank you for submitting your revised manuscript. Referees #2 and #3 have evaluated your revisions, and referee #2 also assessed your responses to referee #1's comments, as this referee was unfortunately unavailable at this stage. As you will see below, the referees are overall satisfied with your revisions. I will therefore be able to accept your manuscript once the following editorial requests have been addressed:

1/ Referees' concerns:
Please address the remaining concerns mentioned by referee #1.

2/ Manuscript text:
- Please remove the red font text and only indicate in track changes mode any new modification to the text.
- Methods:
o Remove the reagent table from manuscript text and upload as a separate file; please correct the citations of Tabs S3 to Appendix Table S3.
o Please include the approval number for the animal experiments granted by the Animal Ethics Committee of the University of Barcelona.
o For the experiments involving human islets, please include a statement confirming that the experiments conformed to the principles set out in the WMA Declaration of Helsinki and the Department of Health and Human Services Belmont Report.
- Please include the Paper Explained in the manuscript text, after the abstract.

3/ Figures:
- Appendix: you are welcome to make some of your Appendix figures into EV figures. If you do so, please make sure to update the figure callouts.
- Please address the query from our data editors in the figure legends:
1. Please note that the exact p values are not provided in the legends of figures 1D, E, H; 2B, C, E, F, I, J; 3B, D, E, F, G; 4B, C, E, F, G, I; 5B, C, F, G, I, J, L; EV1 C, EV2 C, EV3 B, C; EV4 C, D.
2. Please note that information related to n is missing in the legends of figures EV2B, C; EV3 B, C.

4/ Source Data:
Please check the raw data provided for figures 3D and 3E, as identical values were found in different rows.
Please also provide the completed source data checklist.

5/ Synopsis:
I made a minor edit in the stand first of your synopsis, please let me know if you agree:
"Building on prior evidence that Protein Tyrosine Phosphatase 1B (PTP1B) deficiency improves mouse islet graft vascularization, sodium tungstate (NaW), a pharmacological protein phosphatase inhibitor, is shown to enhance vascularization and survival of human beta cell grafts."
The image you provided is very nice, however, for the visual abstract, we need a scheme representing the main findings of the work. Please provide it as a png/tiff/jpeg file 550 px wide x 300 px high.

6/ As part of the EMBO Publications transparent editorial process initiative (see our Editorial at http://embomolmed.embopress.org/content/2/9/329), EMBO Molecular Medicine will publish online a Review Process File (RPF) to accompany accepted manuscripts.
This file will be published in conjunction with your paper and will include the anonymous referee reports, your point-by-point response and all pertinent correspondence relating to the manuscript. Let us know whether you agree with the publication of the RPF and as here, if you want to remove or not any figures from it prior to publication.
Please note that the Authors checklist will be published at the end of the RPF.

I look forward to receiving your revised manuscript.

Yours sincerely,

Lise Roth

Lise Roth, PhD
Senior Editor
EMBO Molecular Medicine

***** Reviewer's comments *****

Referee #2 (Remarks for Author):

Authors have improved some of their analyses, however, some strategies should be further explained:

Rev.1
Authors now calculated NaW provided ad libitum in the drinking water at 1 g/L, with an average consumption estimated at ~125 mg/kg body weight/day, which is way below the LD50. However, in vitro experiments were performed with NaW (0.2 or 1mM), and the secretion/gene expression experiments with 0.2 mM.
It is hard to understand that strategy, as the increase in VEGFA was only observed at the higher dose and insulin data shown at lower dose.
Include doses in the legends and include a clear limitation statement regarding the different dosage used, and how this in vitro dose may relate to the in vivo dose used.

Rev.2
Authors include histological characterization of beta cells in SC-islet grafts (S7), please include representative figures of the beta-cell markers, and refer to Nkx6.1 as the protein. Please also provide representative pictures of the TUNEL analysis in Fig.1, as asked before.
Effects of NaW on SC-islets in vitro (S8), here, the stim index is unusually low, compared to other experiments. Please explain.

Referee #3 (Remarks for Author):

Address all of my original concerns.

Point by point response

Manuscript: EMM-2026-23662

1/ Referees' concerns:
Please address the remaining concerns mentioned by referee #1.

Referee #2 (Remarks for Author):
Authors have improved some of their analyses, however, some strategies should be further explained:

Rev.1
Authors now calculated NaW provided ad libitum in the drinking water at 1 g/L, with an average consumption estimated at ~125 mg/kg body weight/day, which is way below the LD50. However, in vitro experiments were performed with NaW (0.2 or 1mM), and the secretion/gene expression experiments with 0.2 mM.
It is hard to understand that strategy, as the increase in VEGFA was only observed at the higher dose and insulin data shown at lower dose.
Include doses in the legends and include a clear limitation statement regarding the different dosage used, and how this in vitro dose may relate to the in vivo dose used.

Response: We thank the reviewer for this comment. *In vitro* concentrations (0.2–1 mM) were selected to achieve functionally relevant PTP1B inhibition, consistent with the reported Ki for tungstate (~210 µM) (10.1016/j.ccr.2016.07.002) and with concentrations previously shown to exert biological activity across multiple cellular models (e.g.10.1074/jbc.M30833420010.3390/ijms25179150; 10.1186/15476911003631617). The lower concentration (0.2 mM) was applied in SC-islet experiments based on reduced cellular tolerance observed in preliminary assays. All concentrations are now specified in either the graphs or the figure legends, and an explanation of the *in vitro* doses used has been included in the Methods section.

Rev.2
Authors include histological characterization of beta cells in SC-islet grafts (S7), please include representative figures of the beta-cell markers, and refer to Nkx6.1 as the protein. Please also provide representative pictures of the TUNEL analysis in Fig.1, as asked before.
Effects of NaW on SC-islets in vitro (S8), here, the stim index is unusually low, compared to other experiments. Please explain.

Response: Representative images of the beta cell markers in SC-islet grafts have now been included in Appendix Figure S7, and NKX6.1 is now used when referred the protein throughout the manuscript. In addition, representative images of the TUNEL analysis have been added to Figure 1 as requested.

Regarding the low stimulation index observed in Appendix Figure S8, we agree that the response is lower than shown in Appendix Figure S4. However, S8 experiments were performed using SC-islets following shipment to our laboratory and assessed using a static glucose-stimulated insulin secretion assay. It is important to note that static incubation and dynamic perifusion assays (shown in S4) evaluate islet secretory function differently and may yield different estimates of glucose responsiveness (10.1177/09636897251350654). In addition, SC-islets were maintained without ITS-X supplementation before the assay to minimize potential interference from exogenous insulin signaling when assessing the effects of sodium tungstate. Although the reason for the relatively low stimulation index cannot be determined with certainty, no significant differences were observed between NaW-treated and untreated SC-islets, which was the primary objective of this experiment.

Referee #3 (Remarks for Author):

Address all of my original concerns.

28th Aug 2026

Dear Dr. Gasa,

Thank you for submitting your revised files. I am pleased to inform you that your manuscript is accepted for publication and is now being sent to our publisher to be included in the next available issue of EMBO Molecular Medicine.

Your manuscript will be processed for publication by EMBO Press. It will be copy edited and you will receive page proofs prior to publication. Please note that you will be contacted by Springer Nature Author Services to complete licensing and payment information.

You may qualify for financial assistance for your publication charges - either via a Springer Nature fully open access agreement or an EMBO initiative. Check your eligibility: https://link.springer.com/journal/44321/how-to-publish-with-us

Should you be planning a Press Release on your article, please notify the production team via this form in order to coordinate publication and release dates: https://forms.cloud.microsoft/e/eH4A8JVP6g

If you have any questions, please do not hesitate to contact the Editorial Office. Thank you for your contribution to EMBO Molecular Medicine.

With kind regards,

Lise

Lise Roth, Ph.D
Senior Editor
EMBO Molecular Medicine

>>> Please note that it is EMBO Molecular Medicine policy for the transcript of the editorial process (containing referee reports and your response letter) to be published as an online supplement to each paper. If you do NOT want this, you will need to inform the Editorial Office via email immediately. More information is available here: https://link.springer.com/partners/embo-press/editorial-policies#Peer%20review

EMBO Press Author Checklist

Corresponding Author Name: Rosa Gasa	
Journal Submitted to: EMBO Molecular Medicine	
Manuscript Number: EMM-2026-23662	

USEFUL LINKS FOR COMPLETING THIS FORM
The EMBO Journal - Author Guidelines
EMBO Reports - Author Guidelines
Molecular Systems Biology - Author Guidelines
EMBO Molecular Medicine - Author Guidelines

Reporting Checklist for Life Science Articles (updated January
This checklist is adapted from Materials Design Analysis Reporting (MDAR) Checklist for Authors. MDAR establishes a minimum set of requirements in transparent reporting in the life sciences (see Statement of Task: 10.31222/osf.io/9sm4x). Please follow the journal's guidelines in preparing your
Please note that a copy of this checklist will be published alongside your article.

Abridged guidelines for figures
1. Data
The data shown in figures should satisfy the following conditions:
- the data were obtained and processed according to the field's best practice and are presented to reflect the results of the experiments in an accurate and unbiased manner.
- ideally, figure panels should include only measurements that are directly comparable to each other and obtained with the same assay.
- plots include clearly labeled error bars for independent experiments and sample sizes. Unless justified, error bars should not be shown for technical
- if n<5, the individual data points from each experiment should be plotted. Any statistical test employed should be justified.
- Source Data should be included to report the data underlying figures according to the guidelines set out in the authorship guidelines on Data

2. Captions
Each figure caption should contain the following information, for each panel where they are relevant:
- a specification of the experimental system investigated (eg cell line, species name).
- the assay(s) and method(s) used to carry out the reported observations and measurements.
- an explicit mention of the biological and chemical entity(ies) that are being measured.
- an explicit mention of the biological and chemical entity(ies) that are altered/varied/perturbed in a controlled manner.
- the exact sample size (n) for each experimental group/condition, given as a number, not a range;
- a description of the sample collection allowing the reader to understand whether the samples represent technical or biological replicates (including how many animals, litters, cultures, etc.).
- a statement of how many times the experiment shown was independently replicated in the laboratory.
- definitions of statistical methods and measures:
  - common tests, such as t-test (please specify whether paired vs. unpaired), simple χ2 tests, Wilcoxon and Mann-Whitney tests, can be unambiguously identified by name only, but more complex techniques should be described in the methods section;
  - are tests one-sided or two-sided?
  - are there adjustments for multiple comparisons?
  - exact statistical test results, e.g., P values = x but not P values < x;
  - definition of 'center values' as median or average;
  - definition of error bars as s.d. or s.e.m.

Please complete ALL of the questions below.
Select "Not Applicable" only when the requested information is not relevant for your study.

Materials

Newly Created Materials	Information included in the manuscript?	In which section is the information available? (Reagents and Tools Table, Materials and Methods, Figures, Data Availability Section)
New materials and reagents need to be available; do any restrictions apply?	Not Applicable	

Antibodies	Information included in the manuscript?	In which section is the information available? (Reagents and Tools Table, Materials and Methods, Figures, Data Availability Section)
For **antibodies** provide the following information:<br>- Commercial antibodies: RRID (if possible) or supplier name, catalogue number and or/clone number<br>- Non-commercial: RRID or citation	Yes	Reagents and tools table

DNA and RNA sequences	Information included in the manuscript?	In which section is the information available? (Reagents and Tools Table, Materials and Methods, Figures, Data Availability Section)
**Short novel DNA or RNA including primers, probes**: provide the sequences.	Yes	Appendix Figure S3

Cell materials	Information included in the manuscript?	In which section is the information available? (Reagents and Tools Table, Materials and Methods, Figures, Data Availability Section)
**Cell lines:** Provide species information, strain. Provide accession number in repository **OR** supplier name, catalog number, clone number, and/**OR** RRID.	Yes	Reagents and Tools Table
**Primary cultures:** Provide species, strain, sex of origin, genetic modification status.	Yes	Materials an Methods (Human fibroblasts and isolated human islets)
Report if the cell lines were recently **authenticated** (e.g., by STR profiling) and tested for mycoplasma contamination.	Yes	Materials and Methods

Experimental animals	Information included in the manuscript?	In which section is the information available? (Reagents and Tools Table, Materials and Methods, Figures, Data Availability Section)
**Laboratory animals or Model organisms:** Provide species, strain, sex, age, genetic modification status. Provide accession number in repository **OR** supplier name, catalog number, clone number, **OR** RRID.	Yes	Reagents and Tools Table
**Animal observed in or captured from the field:** Provide species, sex, and age where possible.	Not Applicable	
Please detail **housing and husbandry conditions**.	Yes	Material and Methods

Plants and microbes	Information included in the manuscript?	In which section is the information available? (Reagents and Tools Table, Materials and Methods, Figures, Data Availability Section)
**Plants:** provide species and strain, ecotype and cultivar where relevant, unique accession number if available, and source (including location for collected wild specimens).	Not Applicable	
**Microbes:** provide species and strain, unique accession number if available, and source.	Not Applicable	

Human research participants	Information included in the manuscript?	In which section is the information available? (Reagents and Tools Table, Materials and Methods, Figures, Data Availability Section)
If collected and within the bounds of privacy constraints report on age, sex and gender or ethnicity for all study participants.	Not Applicable	

Core facilities	Information included in the manuscript?	In which section is the information available? (Reagents and Tools Table, Materials and Methods, Figures, Data Availability Section)
If your work benefited from core facilities, was their service mentioned in the acknowledgments section?	Yes	Acknowledgements Section

Design

Study protocol	Information included in the manuscript?	In which section is the information available? (Reagents and Tools Table, Materials and Methods, Figures, Data Availability Section)
If study protocol has been **pre-registered, provide DOI in the manuscript**. For clinical trials, provide the trial registration number **OR** cite DOI.	Not Applicable	
Report the **clinical trial registration number** (at ClinicalTrials.gov or equivalent), where applicable.	Not Applicable	

Laboratory protocol	Information included in the manuscript?	In which section is the information available? (Reagents and Tools Table, Materials and Methods, Figures, Data Availability Section)
Provide DOI OR other citation details if **external detailed step-by-step protocols** are available.	Not Applicable	

Experimental study design and statistics	Information included in the manuscript?	In which section is the information available? (Reagents and Tools Table, Materials and Methods, Figures, Data Availability Section)
Include a statement about **sample size** estimate even if no statistical methods were used.	Yes	Material and Methods (Study design sub-section)
Were any steps taken to minimize the effects of subjective bias when allocating animals/samples to treatment (e.g. **randomization procedure**)? If yes, have they been described?	Yes	Material and Methods (Study design sub-section)
Include a statement about **blinding** even if no blinding was done.	Yes	Material and Methods (Study design sub-section)
Describe **inclusion/exclusion criteria** if samples or animals were excluded from the analysis. Were the criteria pre-established?<br><br>If sample or data points were omitted from analysis, report if this was due to attrition or intentional exclusion and provide justification.	Yes	Material and Methods (Study design sub-section)
For every figure, are **statistical tests** justified as appropriate? Do the data meet the assumptions of the tests (e.g., normal distribution)? Describe any methods used to assess it. Is there an estimate of variation within each group of data? Is the variance similar between the groups that are being statistically compared?	Yes	Material and Methods (Statistics sub-section)

Sample definition and in-laboratory replication	Information included in the manuscript?	In which section is the information available? (Reagents and Tools Table, Materials and Methods, Figures, Data Availability Section)
In the figure legends: state number of times the experiment was **replicated** in laboratory.	Yes	Figure legends
In the figure legends: define whether data describe **technical or biological replicates**.	Yes	Figure legends

Ethics

Ethics	Information included in the manuscript?	In which section is the information available? (Reagents and Tools Table, Materials and Methods, Figures, Data Availability Section)
Studies involving **human participants**: State details of **authority granting ethics approval** (IRB or equivalent committee(s), provide reference number for approval.	Yes	Materials and Methods (Human islet sub-section)
Studies involving **human participants**: Include a statement confirming that **informed consent** was obtained from all subjects and that the experiments conformed to the principles set out in the WMA Declaration of Helsinki and the Department of Health and Human Services Belmont Report.	Yes	Materials and Methods (Human islet sub-section)
Studies involving **human participants: For publication of patient photos**, include a statement confirming that consent to publish was obtained.	Not Applicable	
Studies involving experimental **animals**: State details of **authority granting ethics approval** (IRB or equivalent committee(s), provide reference number for approval. Include a statement of compliance with ethical regulations.	Yes	Materials and Methods (Study design sub-section)
Studies involving **specimen and field samples:** State if relevant **permits** obtained, provide details of authority approving study; if none were required, explain why.	Not Applicable	

Dual Use Research of Concern (DURC)	Information included in the manuscript?	In which section is the information available? (Reagents and Tools Table, Materials and Methods, Figures, Data Availability Section)
Could your study fall under dual use research restrictions? Please check biosecurity documents and list of **select agents and toxins** (CDC): https://www.selectagents.gov/sat/list.htm	Not Applicable	
If you used a select agent, is the security level of the lab appropriate and reported in the manuscript?	Not Applicable	
If a study is subject to dual use research of concern regulations, is the name of the **authority granting approval and reference number** for the regulatory approval provided in the manuscript?	Not Applicable	

Reporting

The MDAR framework recommends adoption of discipline-specific guidelines, established and endorsed through community initiatives. Journals have their own policy about requiring specific guidelines and recommendations to complement MDAR.

Adherence to community standards	Information included in the manuscript?	In which section is the information available? (Reagents and Tools Table, Materials and Methods, Figures, Data Availability Section)
State if relevant guidelines or checklists (e.g., **ICMJE, MIBBI, ARRIVE, PRISMA**) have been followed or provided.	Yes	Material and Methods (Study design subsection)
For **tumor marker prognostic studies,** we recommend that you follow the **REMARK** reporting guidelines (see link list at top right). See author guidelines, under 'Reporting Guidelines'. Please confirm you have followed these guidelines.	Not Applicable	
For **phase II and III randomized controlled trials**, please refer to the **CONSORT** flow diagram (see link list at top right) and submit the CONSORT checklist (see link list at top right) with your submission. See author guidelines, under 'Reporting Guidelines'. Please confirm you have submitted this list.	Not Applicable	

Data Availability

Data availability	Information included in the manuscript?	In which section is the information available? (Reagents and Tools Table, Materials and Methods, Figures, Data Availability Section)
Have **primary datasets** been deposited according to the journal's guidelines (see 'Data Deposition' section) and the respective accession numbers provided in the Data Availability Section?	Not Applicable	
Were **human clinical and genomic datasets** deposited in a public access-controlled repository in accordance to ethical obligations to the patients and to the applicable consent agreement?	Not Applicable	
Are **computational models** that are central and integral to a study available without restrictions in a machine-readable form? Were the relevant accession numbers or links provided?	Not Applicable	
If publicly available data were reused, provide the respective **data citations in the reference list**.	Not Applicable	
