# Supplementary material for: Sodium tungstate promotes vascularization to support beta cell replacement in diabetes
Source: EMBO Mol Med. 2026 Sep 23;18(11):4334–56. doi: 10.1038/s44321-026-00523-4 (PMC13748639; doi:10.1038/s44321-026-00523-4)
Supplement: Supplementary file 3 [file 44321_2026_523_MOESM8_ESM.pdf]

Expanded View Figures

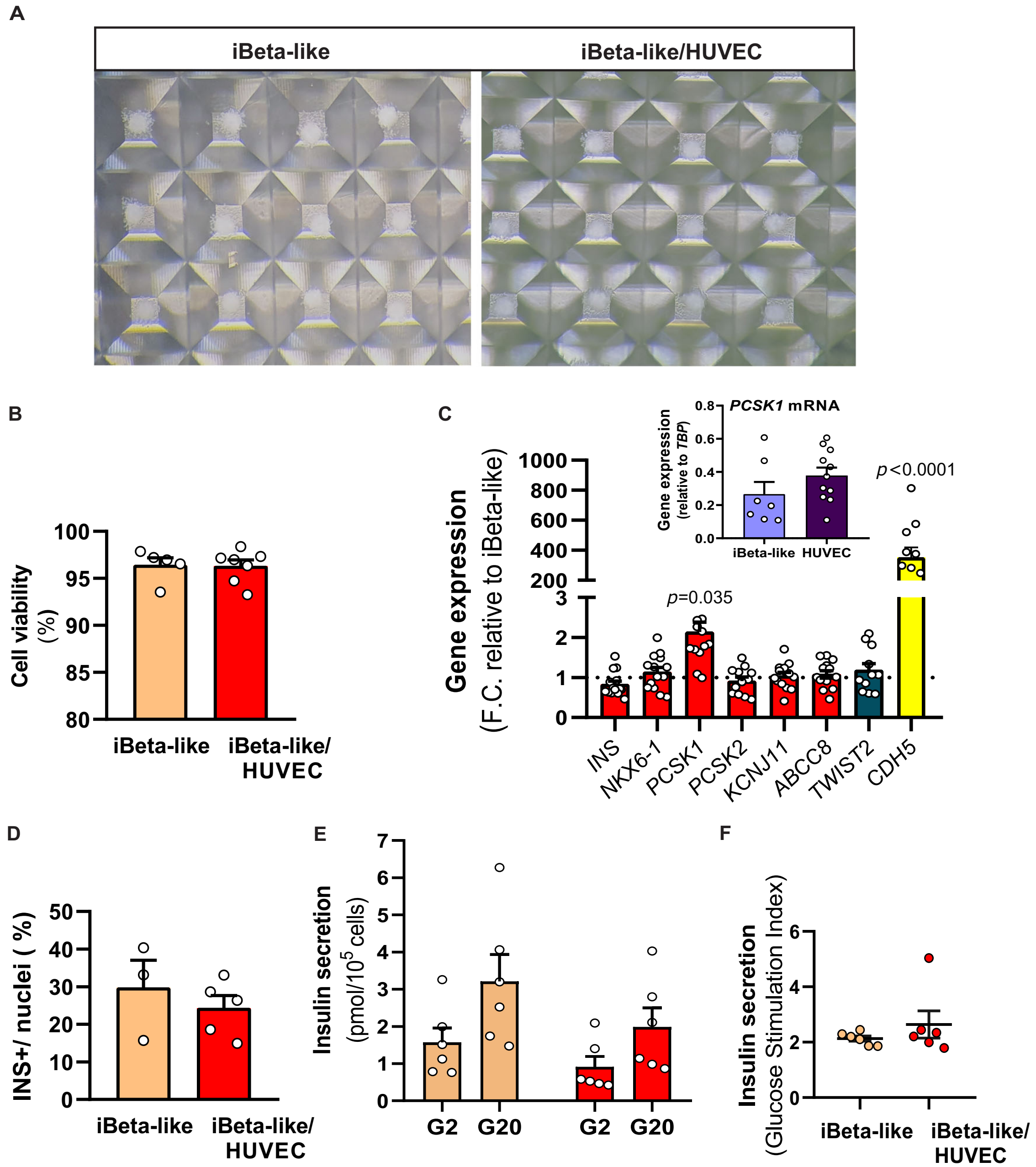

Figure EV1. Characterization of iBeta-like and iBeta-like/HUVEC spheroids.

(**A**) Photographs of AggreWell plates showing spheroids composed of iBeta-like or iBeta-like/HUVEC cells 5 days after seeding single cells into microwells. (**B**) Cell viability as assessed by CFDA and propidium iodide (PI) staining. Cell viability was calculated as the percentage of CFDA$^+$PI$^-$ cells relative to the total number of cells. Each dot represents an individual spheroid. (**C**) Gene expression analysis of the indicated genes by qPCR. Data in the main graph are presented as fold change relative to iBeta-like spheroids, which were set to 1. The inset compares *PCSK1* gene expression in iBeta-like cells and HUVECs; values were normalized to the housekeeping gene *TBP* within each sample. Each dot represents an independent spheroid preparation or HUVEC culture. (**D**) Percentage of INS$^+$ cells in the indicated spheroid types at the end of the reprogramming protocol. For iBeta-like/HUVEC spheroids, CDH5$^+$ cells (corresponding to HUVECs) were excluded from the total nuclei count. Each dot represents an independent reprogramming experiment. (**E**, **F**) Insulin secretion was measured in static incubation assays. Secreted insulin at 2 mM (G2) and 20 mM (G20) glucose is shown in Panel (**E**). The glucose stimulation index, representing the fold change between G20 and G2, is shown in Panel (**F**). Dots represent independent spheroid pools from at least three independent reprogramming experiments. Data were presented as mean ± SEM, with n indicated by individual dots. Statistical analysis was performed using unpaired Student's *t*-test in (**B**, **D**, **F**); one-sample *t*-test in (**C**) and two-way ANOVA in (**E**).

 © The Author(s)

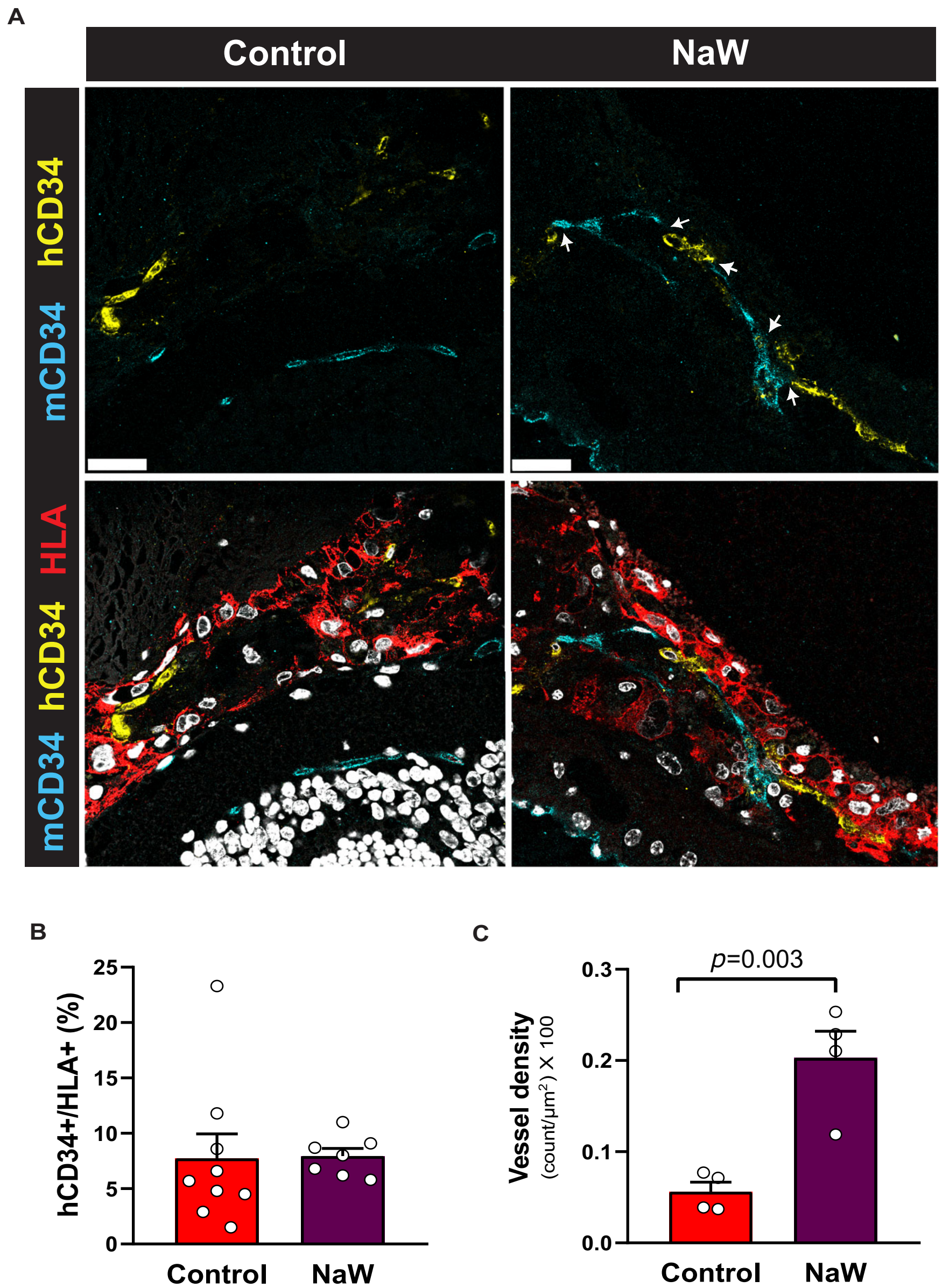

Figure EV2. Histological characterization of HUVEC and mouse ECs in iBeta-like/HUVEC grafts at day 10 post-transplantation.

(**A**) Representative immunofluorescence images of iBeta-like/HUVEC grafts at day 10 post-transplantation in control and NaW-treated mice. Grafts were stained for mouse CD34 (mCD34, cyan; mouse recipient endothelial cells), human CD34 (hCD34, yellow; transplanted HUVECs), HLA (red; transplanted human cells), and Hoechst (white; nuclei). White arrows indicate regions where mouse ECs are adjacent to human ECs. Scale bar: 25 µm. (**B**) Percentage of $hCD34^{+}HLA^{+}$ cells relative to total HLA^{+} cells at day 10 post-transplantation in grafts from control and NaW-treated mice. (**C**) Number of $mCD34^{+}$ vessels relative to HLA^{+} area at day 10 post-transplantation in grafts from control and NaW-treated mice. In (**B**, **C**): data were presented as mean ± SEM, with *n* indicated by individual dots, each dot representing the mean value from an individual graft. Statistical analysis was performed using an unpaired Student's *t*-test.

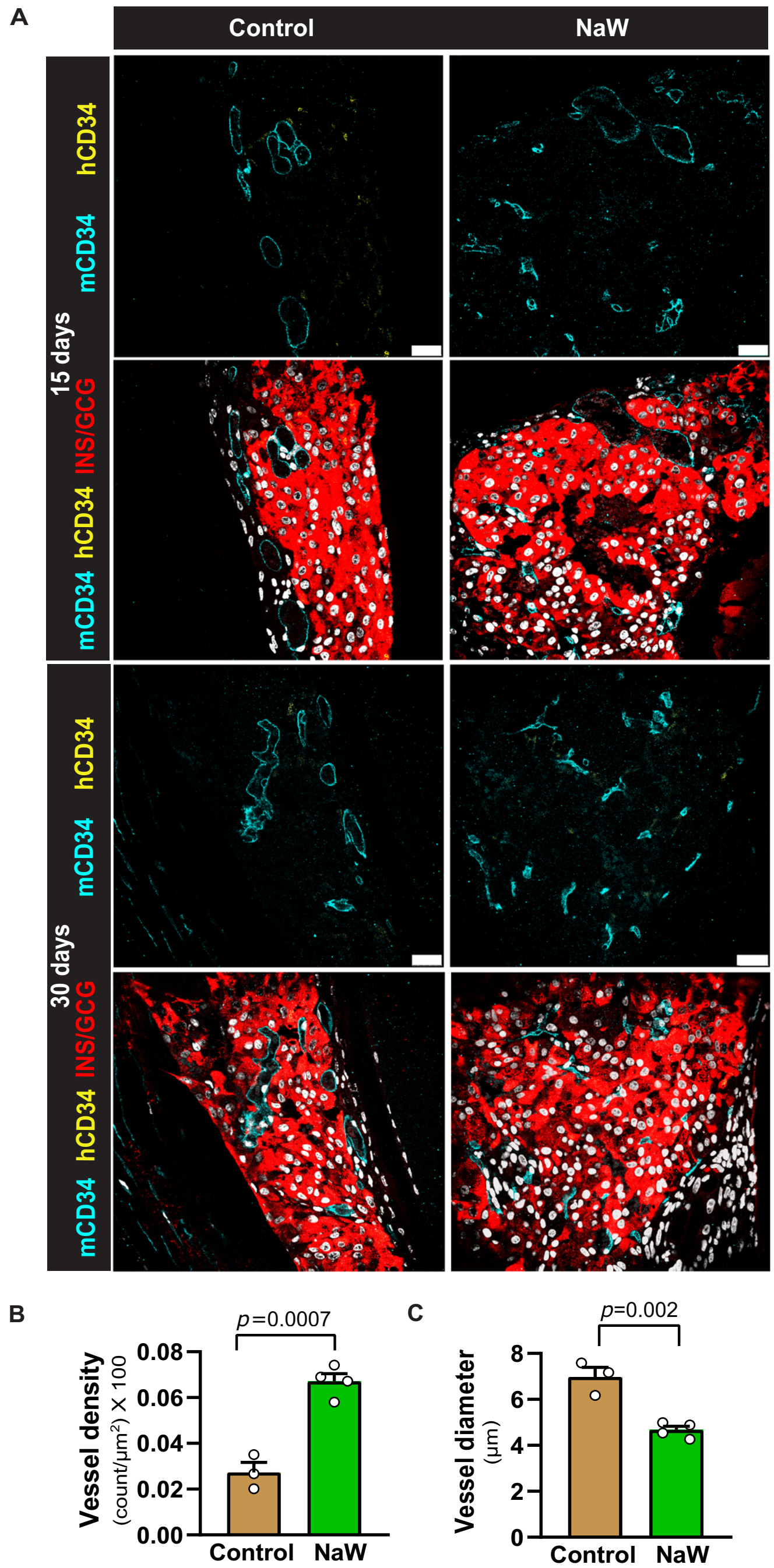

Figure EV3. Histological characterization of human and mouse ECs in SC-islet grafts at days 15 and 30 post-transplantation.

(**A**) Representative immunofluorescence images of SC-islet grafts at days 15 and 30 post-transplantation in control and NaW-treated mice. Grafts were stained for mouse CD34 (mCD34, cyan; mouse recipient endothelial cells), human CD34 (hCD34, yellow; human endothelial cells), INS + GCG (red; endocrine cells), and Hoechst (white; nuclei). Scale bar: 25 μm. (**B**) Number of $mCD34^+$ vessels relative to HLA^+ area at day 10 post-transplantation in grafts from control and NaW-treated mice. (**C**) Average lumen diameter of $mCD34^+$ vessels at day 10 post-transplantation in grafts from control and NaW-treated mice. In (**B**, **C**): data were presented as mean ± SEM, with n indicated by individual dots; each dot represents the mean value from an individual graft. Statistical analysis was performed using an unpaired Student's *t*-test.

 © The Author(s)

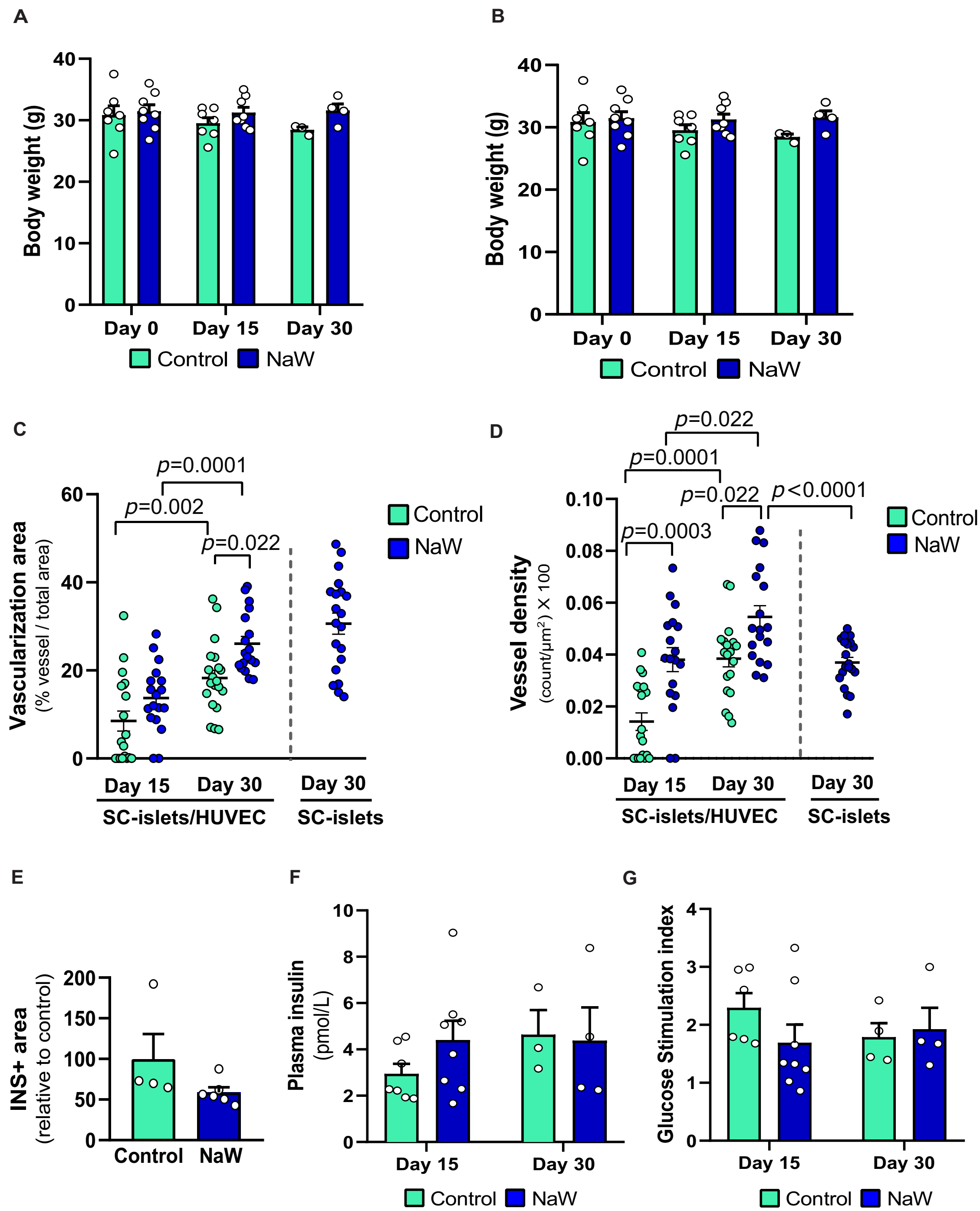

Figure EV4. Effects of NaW treatment on SC-islet/HUVEC grafts.

(**A**) Body weight at the day of transplantation (day 0) and on days 15 and 30 post-transplantation in control and NaW-treated mice. (**B**) Fasting glycemia at days 15 and 30 post-transplantation in control and NaW-treated mice. (**C**, **D**) Quantitative analysis of vascularized area (**C**) and vessel density (**D**) in grafts at days 15 and 30 post-transplantation. For comparative purposes, data obtained from SC-islet grafts (without HUVEC) in NaW-treated mice at day 30 (Fig. 5B,C) are presented on the right of each graft. (**E**) Quantification of insulin-positive area in grafts from control and NaW-treated mice at day 30 post-transplantation. Values are expressed relative to control grafts, with values set to 100%. (**F**) Fasting human plasma insulin in control mice and mice treated with NaW at days 15 and 30 post-transplantation. (**G**) Glucose-stimulated insulin secretion at days 15 and 30 post-transplantation in control and NaW-treated mice. Values are expressed as the fold increase in plasma insulin 20 min after intraperitoneal glucose administration relative to initial fasting insulin levels for each animal (glucose stimulation index). Data were presented as mean ± SEM for the number of *n* indicated by dots. In (**A**, **B**, **F**, **G**): each dot represents an individual mouse. In (**C**, **D**): each dot represents one measured area, from a total of $n = 5$–7 grafts. In (**E**): each dot corresponds to the mean value from an individual graft. Statistical analysis was performed using two-way ANOVA (**A**–**D**, **F**, **G**) and unpaired Student's *t*-test (**E** and in **C**, **D** for the comparison SC-islets and SC-islet/HUVEC at day 30).

© The Author(s)
